# Supplementary material for: Improved discovery of de novo mutations using TrioDNM and VRFS
Source: Gigascience. 2026 Jun 9;15:giag068. doi: 10.1093/gigascience/giag068 (PMC13289757; doi:10.1093/gigascience/giag068)
Supplement: giag068_Supplemental_Files [file giag068_supplemental_files.zip › supplement.pdf]

Supplementary Material

S1 DeNovoGear and TrioDNM implementation

The math notes in [Conrad *et al.*, 2011], Supplementary Notes, and the DeNovoGear code contain some minor errors and in some cases lack clarity.

1. The math notes state that conditional on a segregating site with 1 or 2 alternate alleles, genotypes with 1 alternate allele are found with frequency 3/5 and genotypes with 2 alternate alleles are found with frequency 2/5. This is in fact reversed, there are 4 configurations with a single alternate allele and 6 with two alternate alleles, giving frequencies 2/5 and 3/5, respectively:

```
00 01
00 10
01 00
10 00     .. 4 out of 10 = 2/5

00 11
01 01
10 01
01 10
10 10
11 00     .. 6 out of 10 = 3/5
```

2. It is not clear how the term 0.001 (as opposed to 0.002) has been derived in the math notes. The DeNovoGear code deviates from the math notes, setting for example the prior probability of a single alternate allele in parents as

```
g_priors[i] = 0.995 * 0.002 * (3.0 / 5.0) * (4.0 / 5.0) * 0.5;
```

   or triallelic cases as

```
g_priors[i] = 0.002 * 0.002 / 414;
```

   No explanation is provided as for where the terms 0.995, 0.5 or 414 come from.

3. The DNG code for setting chrX priors has bugs, for example, the transmission of chrX from mother to a son does not depend on the paternal genotype. For this reason bcftools/trio-dnm3 does not attempt to reimplement this part of the code.

Revised priors

Following [Conrad *et al.*, 2011], the prior probability $P(G_M, G_F)$ of drawing two parental genotypes from the population is loosely derived neutral coalescent model. Empirically we know that two human chromosomes in European populations differ at approximately 1/1000 sites. There are 4N generations in a tree of two chromosomes, and $N \cdot 100/12$ generations in a tree relating 5 chromosomes, so we assume approximately 2/1000 sites will be variable in a sample of 5 human chromosomes. Then the prior for sampling G_M and G_F as homozygous reference is 0.998. For the remaining, we consider the number of alternate alleles (up to 3) and the number of configurations the alleles can be distributed across parental chromosomes (for example, there are 4 out of the 15 possible biallelic combinations which have one alternate allele).

Label the reference allele 0 and consider at most three non-reference alleles $x \in \{1, 2, 3\}$. Set the prior probabilities of sampling reference and alternate genotypes as follows

$P_1 = 0.998$
: the probability of sampling two reference genotypes at sites with one alternate allele common in the population (biallelic sites)

$P_3 = (1 - P_1)^2$
: the probability of sampling a non-reference genotype at sites with two different alternate alleles common in the population (triallelic sites)

$P_2 = 1 - P_1 - P_3 \approx 1 - P_1$
: the probability of sampling a non-reference genotype at biallelic sites

$P_4 = 10^{-26}$
: the probability of observing tetra-allelic site

Autosomal chromosomes

The prior probability of sampling four chromosomes from the population for parental genotypes M and F is then as follows

$$P(M,F)=\begin{cases}\text{0 alternate alleles:} & \\ \quad P_1 & M,F=00,00\\ \text{1 alternate allele:} & \\ \quad P_2\cdot(1/15)\cdot(1/3) & M,F\in\{xx,xx\}\text{ where }x\in\{1,2,3\}\\ \quad P_2\cdot(2/15)\cdot(1/3) & M,F\in\{00,xx\ ;\ xx,00\}\\ \quad P_2\cdot(4/15)\cdot(1/3) & M,F\in\{0x,xx\ ;\ x0,xx\ ;\ xx,0x\ ;\ xx,x0\}\\ \quad P_2\cdot(4/15)\cdot(1/3) & M,F\in\{0x,0x\ ;\ 0x,x0\ ;\ x0,0x\ ;\ x0,x0\}\\ \quad P_2\cdot(4/15)\cdot(1/3) & M,F\in\{00,0x\ ;\ 00,x0\ ;\ 0x,00\ ;\ x0,00\}\\ \text{2 alternate alleles:} & \\ \quad P_3\cdot(1/19)\cdot(1/3) & M,F\in\{00,xy\ ;\ 0x,xy\ ;\ \dots\}\\ \text{3 or more alternate alleles:} & \\ \quad P_4 & \end{cases}$$

Chromosome X in males

For chromosome X inheritance pattern in males we define analogously

$$\begin{aligned}P_{X,1}&=0.999\\ P_{X,3}&=(1-P_{X,1})^2\\ P_{X,2}&=1-P_{X,1}-P_{X,3}\approx 1-P_{X,1}\end{aligned}$$

The priors for drawing the maternal genotype then become

$$P_X(M)=\begin{cases}\text{0 alternate alleles:} & \\ \quad P_{X,1} & M=00\\ \text{1 alternate allele:} & \\ \quad P_{X,2}\cdot(1/3)\cdot(1/3) & M=xx\text{ where }x\in\{1,2,3\}\\ \quad P_{X,2}\cdot(2/3)\cdot(1/3) & M\in\{0x\ ;\ x0\}\\ \text{2 alternate alleles:} & \\ \quad P_{X,3}\cdot(1/3) & M\in\{xy\ ;\ xz\ ;\ yz\}\end{cases}$$

Chromosome X in females

For chromosome X inheritance in females we approximate as

$$P_{XX}(M,F)=\begin{cases}\text{0 alternate alleles:} & \\ \quad P_1 & M,F=00,0\\ \text{1 alternate allele:} & \\ \quad P_2\cdot(3/7)\cdot(1/3) & M,F\in\{00,x\ ;\ 0x,0\ ;\ x0,0\}\text{ where }x\in\{1,2,3\}\\ \quad P_2\cdot(3/7)\cdot(1/3) & M,F\in\{0x,x\ ;\ 0x,x\ ;\ xx,0\}\\ \quad P_2\cdot(1/7)\cdot(1/3) & M,F=xx,x\\ \text{2 alternate alleles:} & \\ \quad P_3\cdot(1/9)\cdot(1/3) & M,F\in\{0x,y\ ;\ \dots\ ;\ xx,y\ ;\ \dots\ ;\ xy,y\ ;\ \dots\}\end{cases}$$

S2 Usage examples

All variants of the `+trio-dnm3` usage below annotate a VCF file with the computed score (stored by default in the FORMAT/DNM tag), the index of the variant allele (FORMAT/VA), and the frequency of the variant allele (FORMAT/VAF).

Naive *de novo* calling

Relies on FORMAT/GT and is not recommended due to its high false-positive rate. This mode is used for generating a candidate callset to be further refined by one of the DNG, ALM, or DMM models.

```
bcftools +trio-dnm3 -p proband,father,mother --use-NAIVE input.vcf
```

DNG, the DeNovoGear model

The original DeNovoGear model, uses FORMAT/PL and the original DNG priors

```
bcftools mpileup -f ref.fa proband.bam father.bam mother.bam |  # generate genotype likelihoods
bcftools call -mA |                                             # perform basic calling, gets rid of <*>
bcftools norm -f ref.fa |                                       # normalize indels
bcftools +trio-dnm3 -p proband,father,mother --use-DNG          # annotate with the DNG probability score
```

DeNovoGear with fixed priors

The DeNovoGear model used with FORMAT/PL with the revised priors. All commands of the pipeline are analogous to the above, note the use of `--use-ALM` and `--with-pPL`

```
bcftools mpileup -f ref.fa proband.bam father.bam mother.bam |
bcftools call -mA |
bcftools norm -f ref.fa |
bcftools +trio-dnm3 -p proband,father,mother --use-ALM --with-pPL
```

ALM, the allele-likelihood model

Basic allele-likelihood model. All commands of the pipeline are analogous to the above, note the use of `mpileup -a QS` and `--use-ALM`

```
bcftools mpileup -f ref.fa proband.bam father.bam mother.bam -a QS |
bcftools call -mA |
bcftools norm -f ref.fa |
bcftools +trio-dnm3 -p proband,father,mother --use-ALM
```

DMM, the Dirichlet–multinomial model

Basic allele-likelihood model. All commands of the pipeline are analogous to the above, note the use of `mpileup -a QM` and `--use-DMM`

```
bcftools mpileup -f ref.fa proband.bam father.bam mother.bam -a QM |
bcftools call -mA |
bcftools norm -f ref.fa |
bcftools +trio-dnm3 -p proband,father,mother --use-DMM
```

Variant Read Frequency Score

The variant read frequency score method was written with performance and ease of use in mind, and can easily be run in parallel. In order to run the program efficiently see below how to decide on two important aspects: whether to stream entire bams (many tested sites) or use the index (few sites); and how many bams to run in parallel. Below we show how to run the program in batches; for a single run just leave out the `--batch` option.

```
# First decide whether to use index or not. Run with -v in the verbose mode, the program prints
# the time required to process one alignment file. Kill with ctrl+c when done.
bcftools +vrfs -a bams-list.txt -f ref-genome.fa -s sites.txt -v       # stream the entire bam
bcftools +vrfs -a bams-list.txt -f ref-genome.fa -s sites.txt -v -i    # use index to jump

# Secondly, decide how many bams to run per batch. Say the output above suggests it is feasible
# to run 30 bams per batch, now find out how many batches are needed.
bcftools +vrfs -a bams-list.txt --batch k=30
```

```
# Now run the batches in parallel, say the above output suggested 100 batches are required.
bcftools +vrfs -f ref.fa -a bams.txt -s sites.txt -o scores1.txt --batch 1/100
bcftools +vrfs -f ref.fa -a bams.txt -s sites.txt -o scores1.txt --batch 2/100
bcftools +vrfs -f ref.fa -a bams.txt -s sites.txt -o scores1.txt --batch 3/100
...
bcftools +vrfs -f ref.fa -a bams.txt -s sites.txt -o scores100.txt --batch 100/100

# Finally, merge the results
bcftools +vrfs --merge-files scores*.txt -o merged.txt
```

S3 Variant Read Frequency Score

A

619-5-1-7-19-51-57-71-89-77-84-60-45-30-6-15-12-44-9-170

B

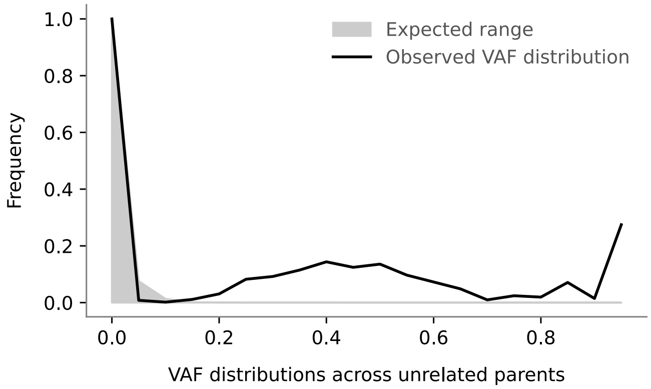

C

```
1981-0-0-0-0-0-0-0-0-0-0-0-0-0-0-0-0-0-0-0-0
1980-1-0-0-0-0-0-0-0-0-0-0-0-0-0-0-0-0-0-0-0
1975-4-0-0-0-0-0-0-0-0-0-0-0-0-0-0-0-0-0-0-0
1477-3-0-0-0-0-0-0-0-0-0-0-0-0-0-0-0-0-0-0-0
1350-3-0-0-0-0-0-0-0-0-0-0-0-0-0-0-0-0-0-0-0
 812-2-0-0-0-0-0-0-0-0-0-0-0-0-0-0-0-0-0-0-0
1980-1-0-0-0-0-0-0-0-0-0-0-0-0-0-0-0-0-0-0-0
1961-3-0-0-0-0-0-0-0-0-0-0-0-0-0-0-0-0-0-0-0
1947-6-0-0-0-0-0-0-0-0-0-0-0-0-0-0-0-0-0-0-0
 627-7-0-0-0-0-0-0-0-0-0-0-0-0-0-0-0-0-0-0-0
 586-0-0-0-0-0-0-0-0-0-0-0-0-0-0-0-0-0-0-0-0
1164-0-0-0-0-0-0-0-0-0-0-0-0-0-0-0-0-0-0-0-0
 932-0-0-0-0-0-0-0-0-0-0-0-0-0-0-0-0-0-0-0-0
 368-0-0-0-0-0-0-0-0-0-0-0-0-0-0-0-0-0-0-0-0
 835-0-0-0-0-0-0-0-0-0-0-0-0-0-0-0-0-0-0-0-0
1006-0-0-0-0-0-0-0-0-0-0-0-0-0-0-0-0-0-0-0-0
 ...
```

Supplementary Figure S3: A-B) An example of a VAF distribution collected across 1471 samples and 20 VAF bins. In this example, 619 samples showed no alternate allele (VAF=0) at this genomic position, 84 samples had approximately half of the reads supporting the alternate allele (VAF=0.5), and in 170 samples had only alternate allele (VAF=1). **C**) Example of VAF distributions collected at high-confidence sites, used to estimate the variance within each VAF bin (see also the script `misc/vrfs-variances` in the `bcftools` package).

S4 The sensitivity of VRFS to VAF bin size

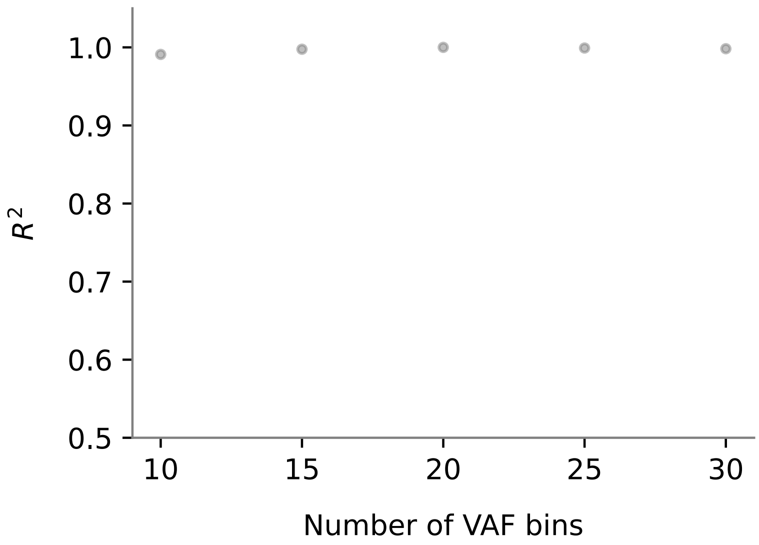

Supplementary Figure S4: The stability of VRFS calculation was tested on the full set of prefiltered candidate variants. The calculation proved to be stable with respect to the number of VAF bins.

S5 The sensitivity of VRFS to VAF variances

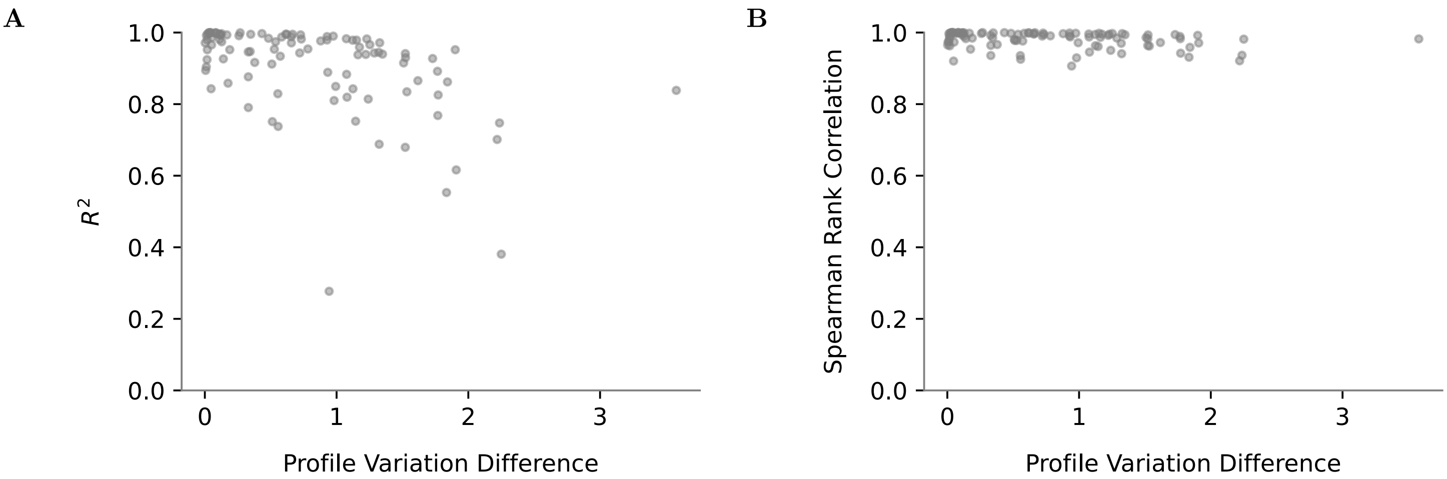

Supplementary Figure S5: The calculation of VRFS values is based on VAF profile variances $\{\sigma_i^2\}$, which are precomputed from a set of high-confidence sites. In these graphs, we assess the sensitivity of the VRFS scores to the choice of reference sites and to perturbations in the profile variances, and consequently, evaluate the transferability of these profiles across datasets. Each data point represents the difference betwen two VRFS calculations performed on the entire dataset using two distinct sets of profile variances, $\{\sigma_i^2\}$ and $\{\sigma_i'^2\}$, each derived from a different set of high-confidence sites using the `misc/vrfs-variances` script from the `bcftools` package.
The x-axis quantifies the difference between two profiles $\{\sigma_i^2\}$ and $\{\sigma_i'^2\}$. It is calculated as $\Delta = \sum_i (\sigma_i - \sigma_i') \cdot i$ and can be viewed as the expected difference in the variation of the total number of alternate reads across all reference samples. Lower values indicate more similar profile variances, suggesting the resulting VRFS values will also be closer. (**A**) Comparable profile variances result in similar VRFS values, suggesting that the choice of reference sites does not significantly alter the outcome when variances are alike. (**B**) Importantly, the relative rank order of these VRFS values remains consistent and stable.

S6 Variant read frequency score distribution

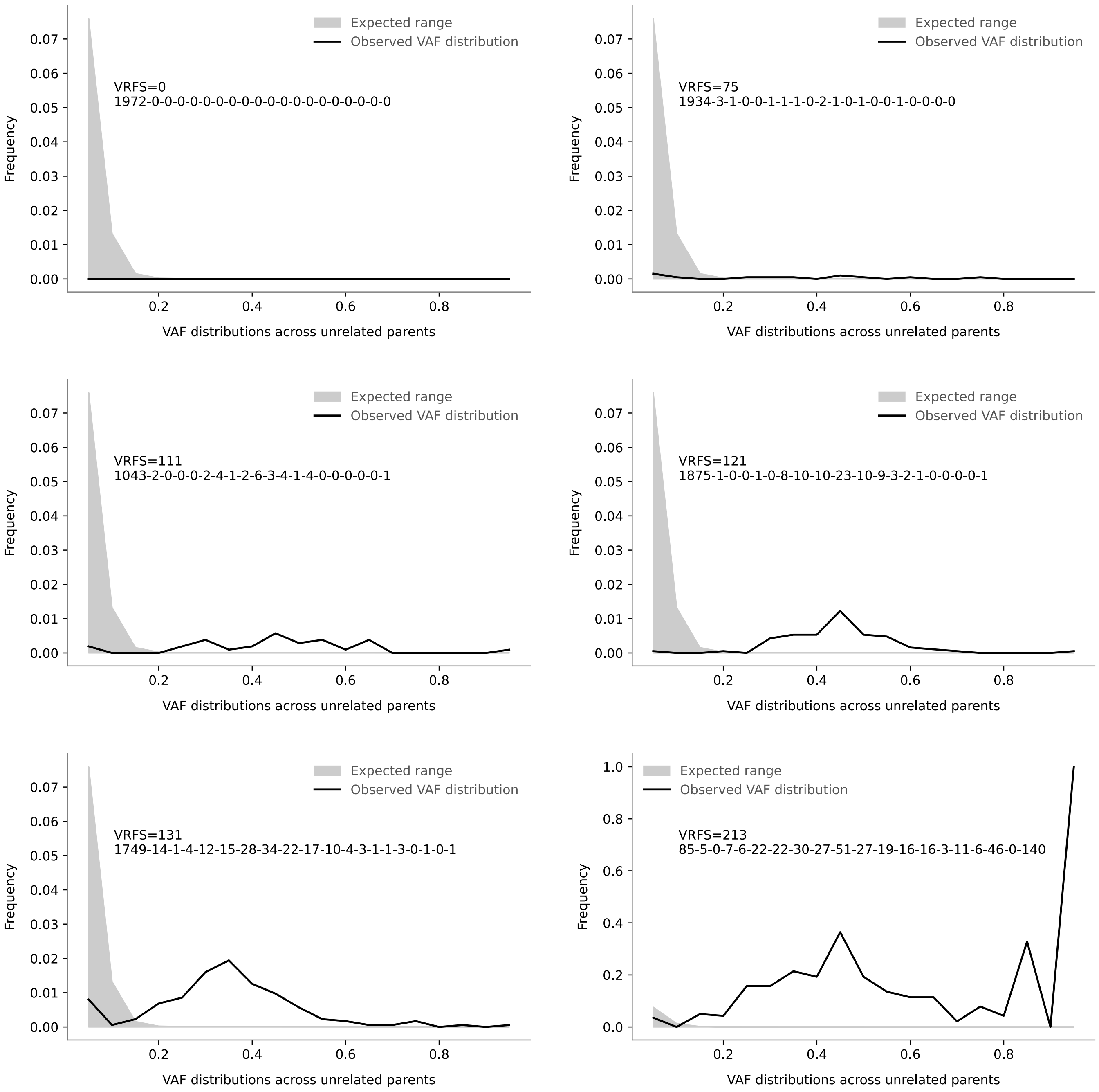

Supplementary Figure S6: Examples of parental VAF distributions at six sites and the corresponding variant read frequency scores across a range of values, from VRFS=0 (alternate allele not present in the samples) to VRFS=213 (alternate allele is prevalent in the samples, with 140 having alternate homozgyous genotype).

S7 Simulated data for DNG, ALM, and DMM evaluation

We constructed four parental haplotypes by generating two phased parental VCF files from randomly selected Genome Aggregation Database (gnomAD) variants common in individuals of non-Finnish European ancestry (NFE_AF > 0.001). Genotypes were simulated under Hardy–Weinberg equilibrium, with heterozygous variants assigned at random to one of the two parental haplotypes to achieve phasing.

For the child, 5,000 genuine *de novo* SNVs observed across samples in the studied cohort (BiB) were introduced into the transmitted haplotypes. Individual-specific reference sequences were then generated using BCFtools/consensus, producing six FASTA sequences (two per individual). Sequencing reads were simulated using Mason (150 bp paired-end reads; mean fragment size 350 bp, SD 50), aligned to the reference genome using BWA-MEM, and variants were called using BCFtools/mpileup followed by BCFtools/trio-dnm3.

The resulting callset comprised 4,987 variants classified as true positives, 1,039 as false positives, and 2 as false negatives. Receiver operating characteristic (ROC) curves were generated for each scoring method by varying the quality threshold. Both DMM and ALM demonstrated improved discrimination between true and false calls compared to DNG.

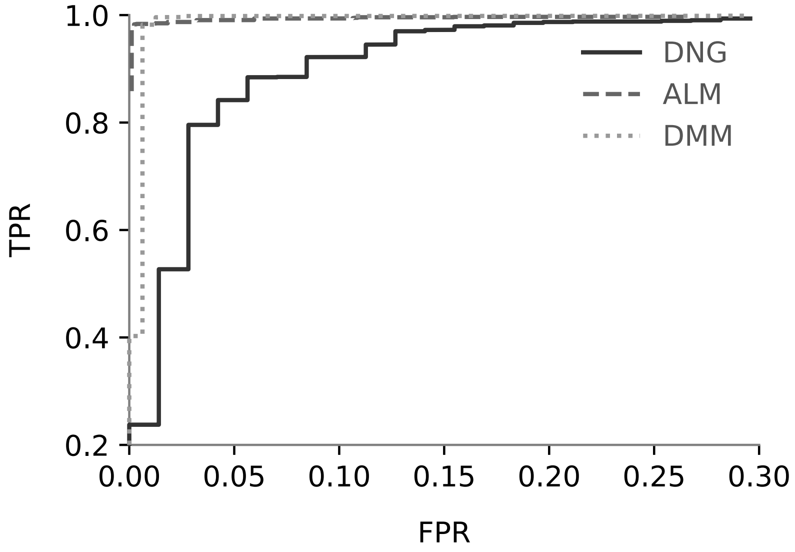

Supplementary Figure S7: Receiver operating characteristic (ROC) curves for DNG, ALM, and DMM evaluated on simulated trio data. True positive rate (TPR) is plotted against false positive rate (FPR) across varying score thresholds. Areas under the curve (AUC) were 0.967 for DNG, 0.995 for ALM, and 0.995 for DMM.

S8 Distribution of DNG, ALM, and DMM scores among curated calls

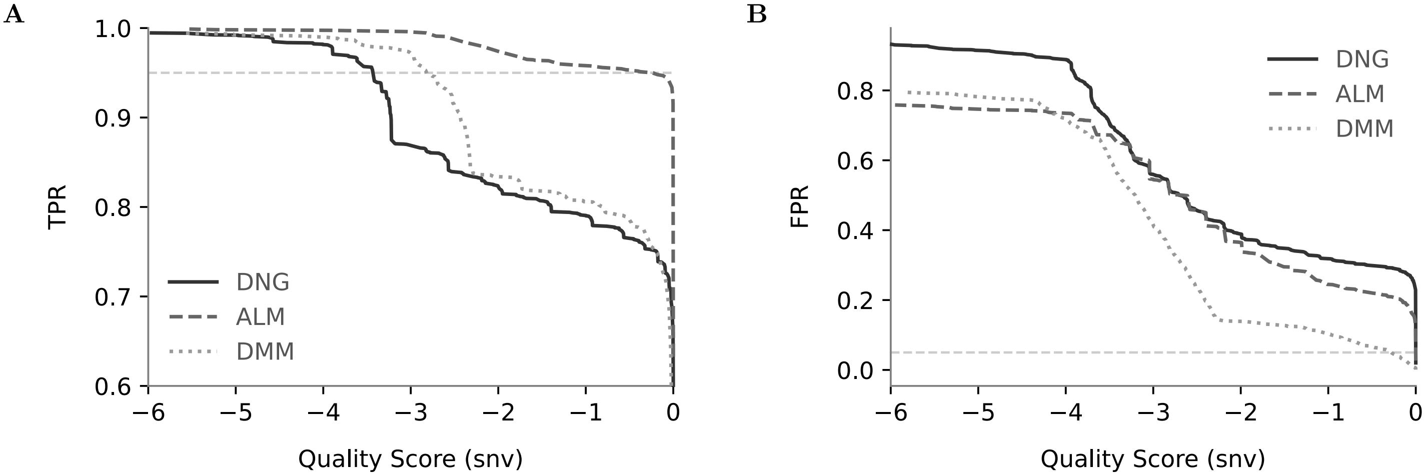

Supplementary Figure S8: (**A**) True positive rate (TPR) as a function of DNG, ALM, and DMM quality scores for manually curated candidate sites. The dashed horizontal line indicates 95% TPR used to define thresholds for construction of the clean callset for VRFS evaluation: -3.43691, -0.335119, and -2.81869 for DNG, ALM, and DMM, respectively. (**B**) False positive rate (FPR) as a function of DNG, ALM, and DMM quality scores for manually curated candidate sites. The dashed horizontal line marks 5% FPR, corresponding to thresholds of -3.8e-10, -3.2e-8, and -0.28 for DNG, ALM, and DMM, respectively.

S9 Ti/Tv by callset size

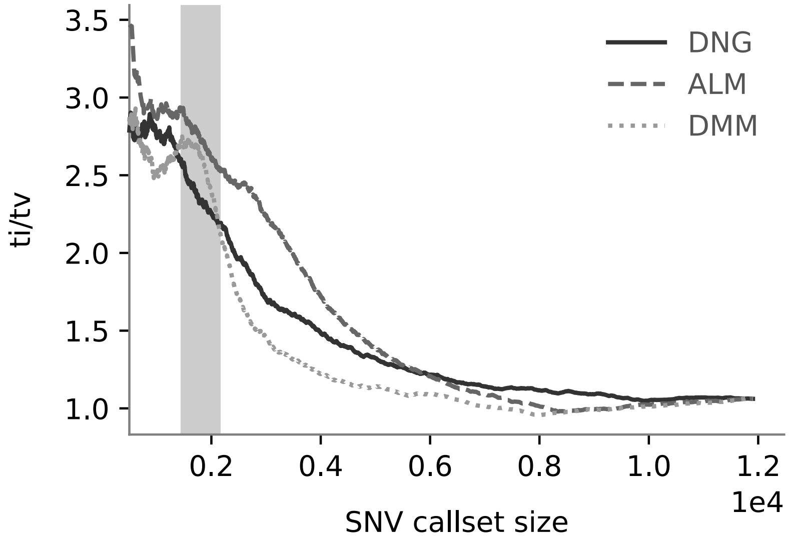

Supplementary Figure S9: Transition-to-transversion ratio (ti/tv) in the raw callset, shown as a function of SNV callset size. Variants were ranked in descending order by DNG, ALM, or DMM score, and ti/tv was computed cumulatively as increasingly larger sets of top-scoring candidates were included. The left side corresponds to the most stringent score thresholds; the right side includes all candidate sites. Higher ti/tv values reflect stronger enrichment for genuine biological variation, though not specifically for *de novo* events. The grey bar marks the expected approximate number of true *de novo* mutations.

S10 VAF25 by callset size

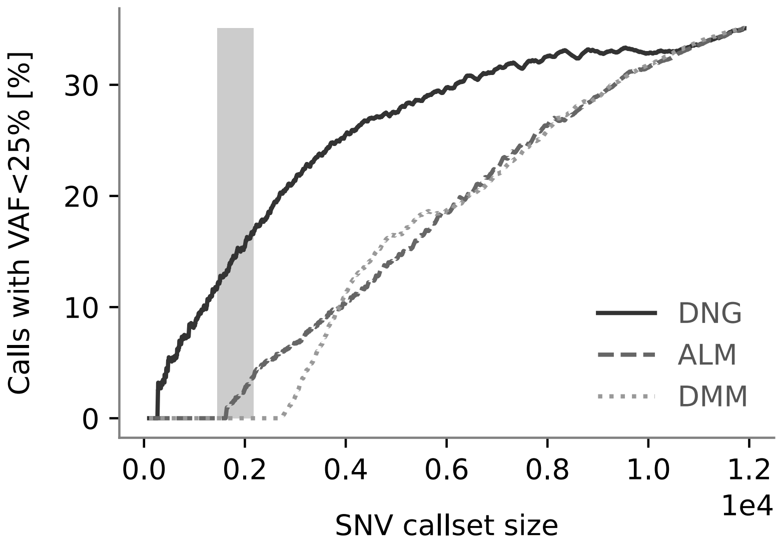

Supplementary Figure S10: Proportion of calls in the raw callset with variant allele fraction $< 25\%$ (VAF25), shown as a function of SNV callset size. Candidate variants were ranked in descending order by DNG, ALM, or DMM score, and the VAF25 metric was computed cumulatively as increasingly larger sets of top-scoring candidates were included. The left side corresponds to the most stringent score thresholds; the right side includes all candidate sites. Lower values indicate a cleaner callset with fewer low-VAF events, which are often enriched for sequencing artefacts. The grey bar denotes the expected approximate size of the true *de novo* callset.

S11 Modeling parental allele emission improves filtering performance

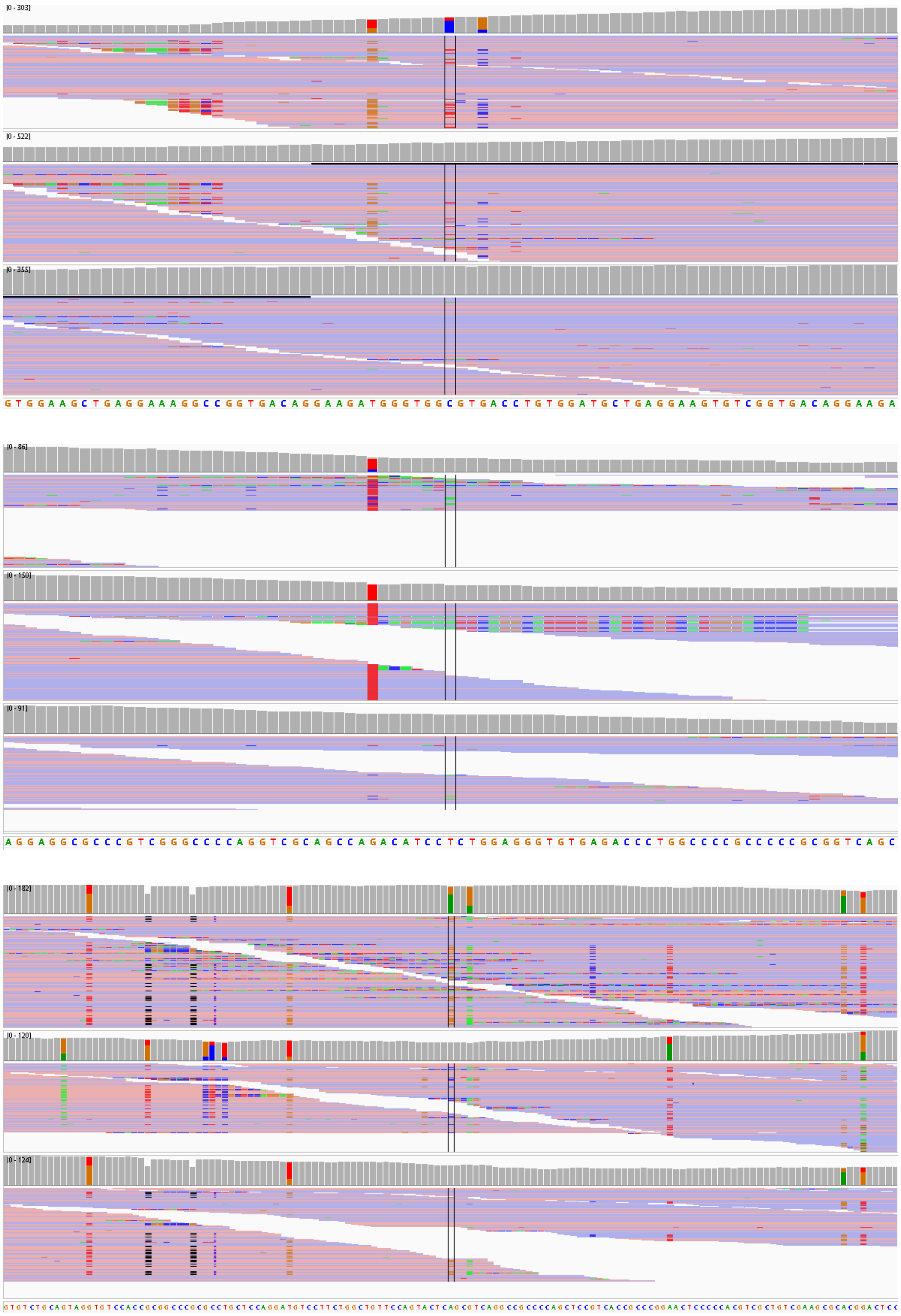

Supplementary Figure S11: Three examples of false *de novo* calls with high DNG scores but low ALM and DMM scores illustrate the importance of modeling parental allele emission. In the IGV snapshots, the proband is shown in the top track, followed by the father and mother. In all three cases, the candidate allele is consistent with a mapping artefact. Moreover, the alternate allele is detectable in one or both parents, indicating that–even if genuine–it is not a true *de novo* event.

S12 The impact of sequencing depth on performance

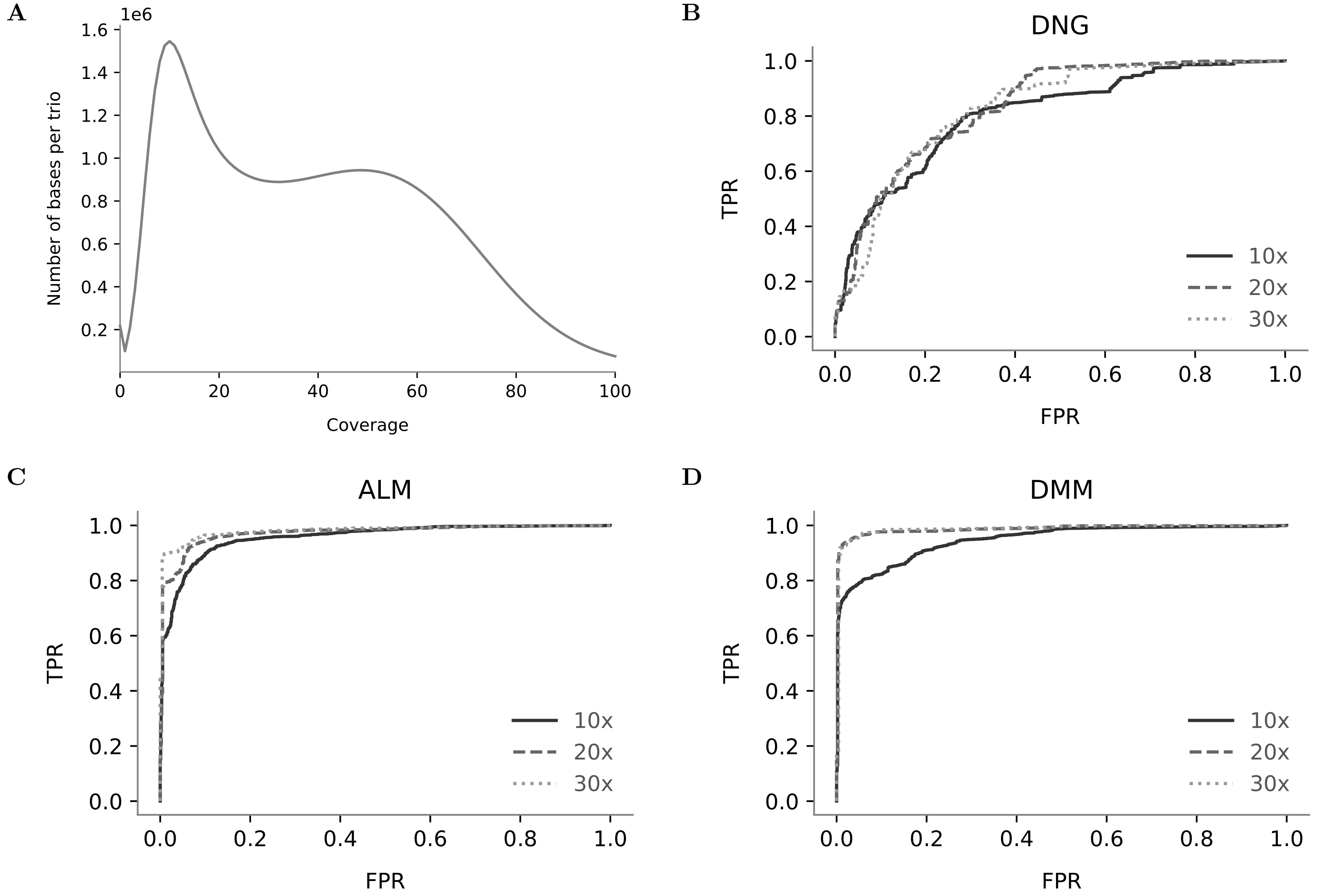

Supplementary Figure S12: **A**) The distribution of exome sequencing depth in the target regions of the BiB cohort, shown across all trios and sites. For each site, the minimum depth within the trio was used. The median depth was 38× and the average depth 41×. **B-D**) Receiver operating characteristic (ROC) curves for DNG, ALM, and DMM evaluated at 10×, 20×, and 30× coverage.

S13 Recurrence by callset size

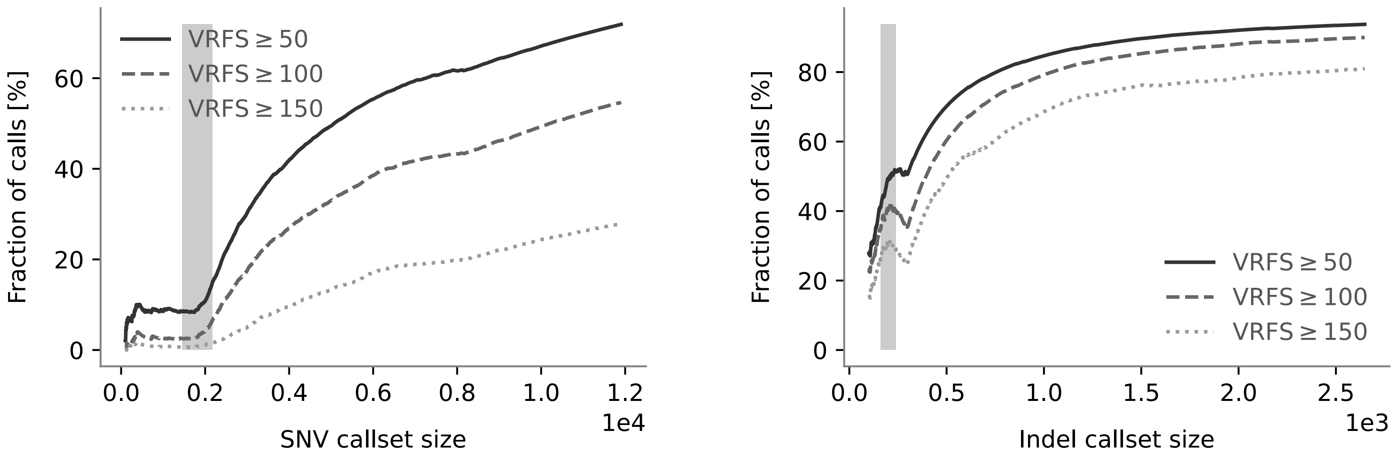

Supplementary Figure S13: The proportion of recurrent calls (VRFS≥50, VRFS≥100, and VRFS≥150) in the raw callset, shown as a function of callset size. Candidate variants were ranked in descending order by DMM score, and the fraction of recurrent variants was computed cumulatively as progressively larger sets of top-scoring candidates were included. The left side of the plot corresponds to the most stringent score thresholds, whereas the right side includes all candidate sites. Lower values indicate a cleaner callset with fewer highly recurrent events, which are often enriched for sequencing artefacts. The grey bar denotes the expected approximate size of the true *de novo* callset.

S14 Repeat content

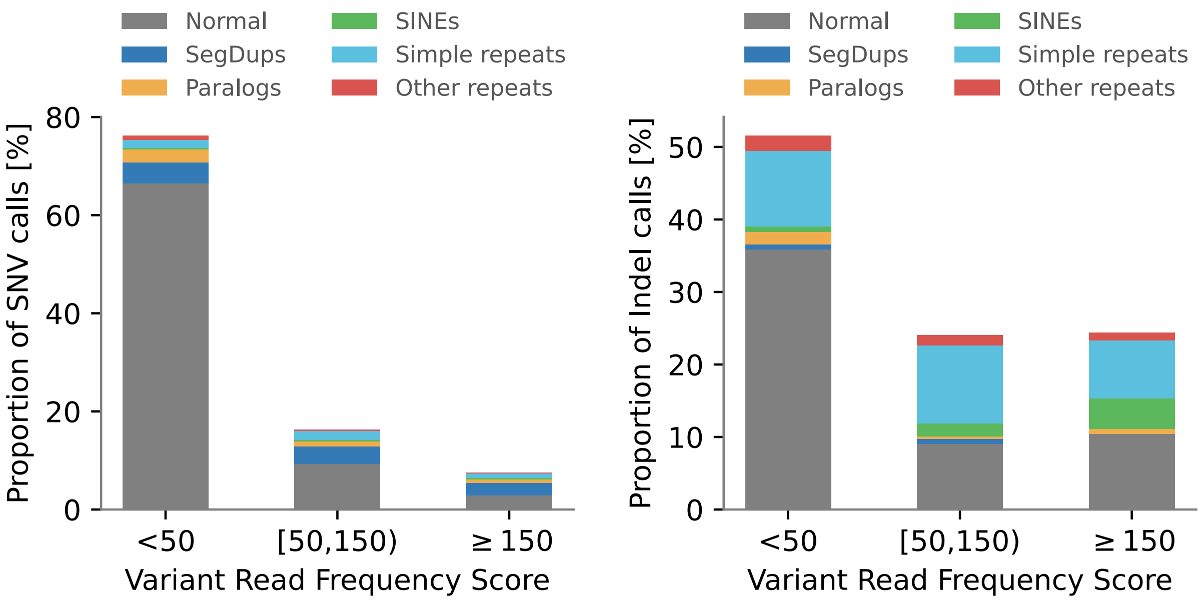

Supplementary Figure S14: Proportion of *de novo* SNVs (left) and indels (right) in the clean callset found in paralogs, segmental duplications and repetitive regions of the genome, split by the variant read frequency score.

S15 Missed inheritance

A potential source of recurrence at sites with apparent *de novo* calls is true polymorphism, where the paternal alternate allele in heterozygous genotypes is missed due to random sampling of reads during sequencing. The number of such misclassified inherited variants can be estimated from the variant read frequency scores by evaluating the probability of failing to sample the alternate allele

$$P(\text{inherited}) \approx (B_{\text{F}} + B_{\text{M}}) \cdot P_{\text{het}},$$

where B_{F} and B_{M} are the parental binomial probabilities of sampling the observed number of alternate reads given their sequencing depth, and the probability of heterozygous genotype P_{het} is estimated from the variant read frequency score distributions as the proportion of samples with VAF between $(0.25, 0.75)$.

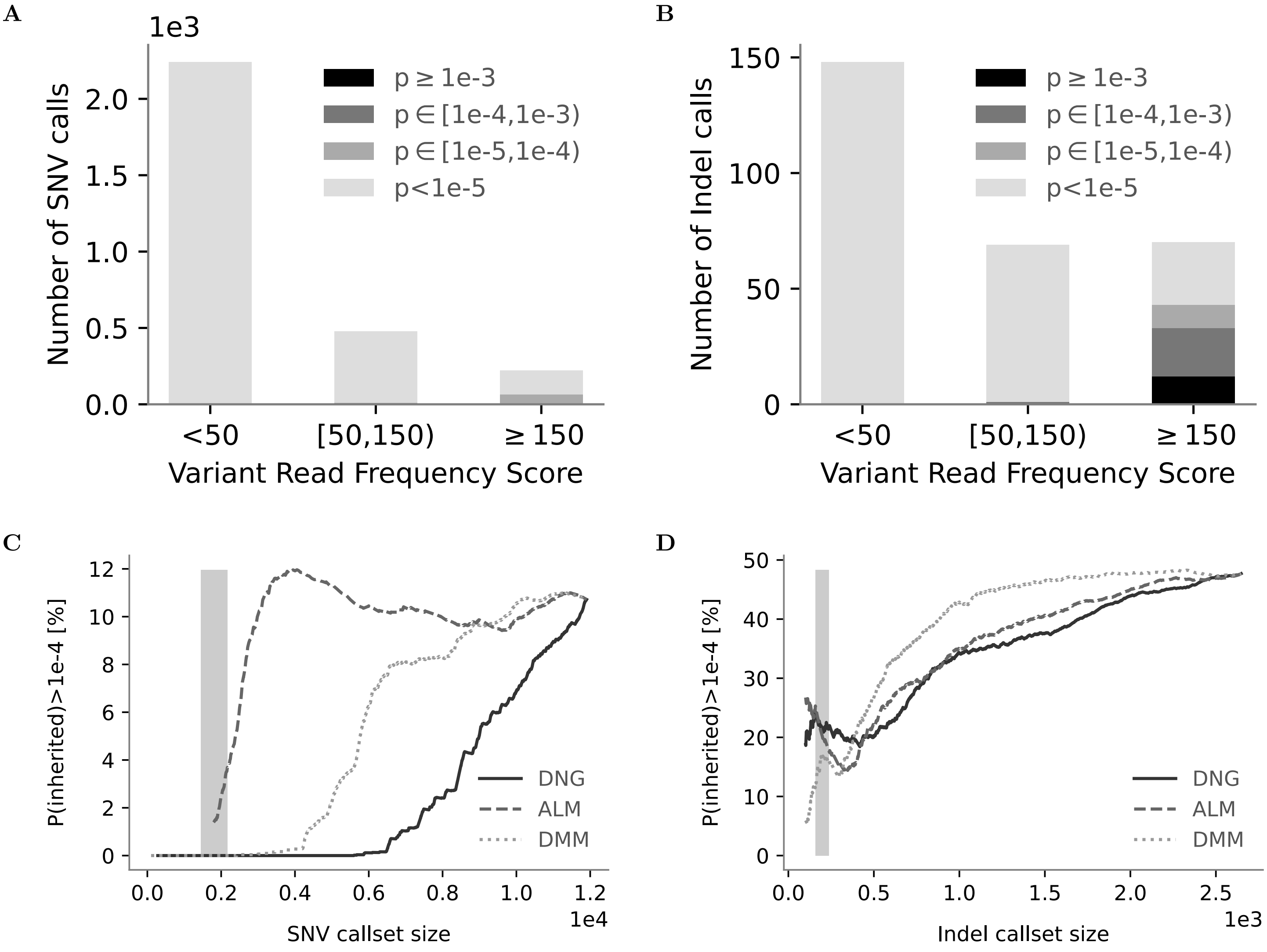

Supplementary Figure S15: A-B) Number of potentially inherited SNVs (left) and indels (right) in the clean callset misinterpeted as *de novo* variants with the probability of not sampling the alternate allele in parents indicated in the legend. **C-D**) Proportion of potentially inherited SNV and indel calls in the raw callset misinterpreted as *de novo*, with $P(\text{inherited}) > 10^{-4}$, shown as a function of callset size. Candidate variants were ranked in descending order by DNG, ALM, or DMM score, and the probability was computed cumulatively as increasingly larger sets of top-scoring candidates were included. The left side corresponds to the most stringent score thresholds; the right side includes all candidate sites. The grey bar denotes the expected approximate size of the true *de novo* callset.

S16 Presence in gnomAD

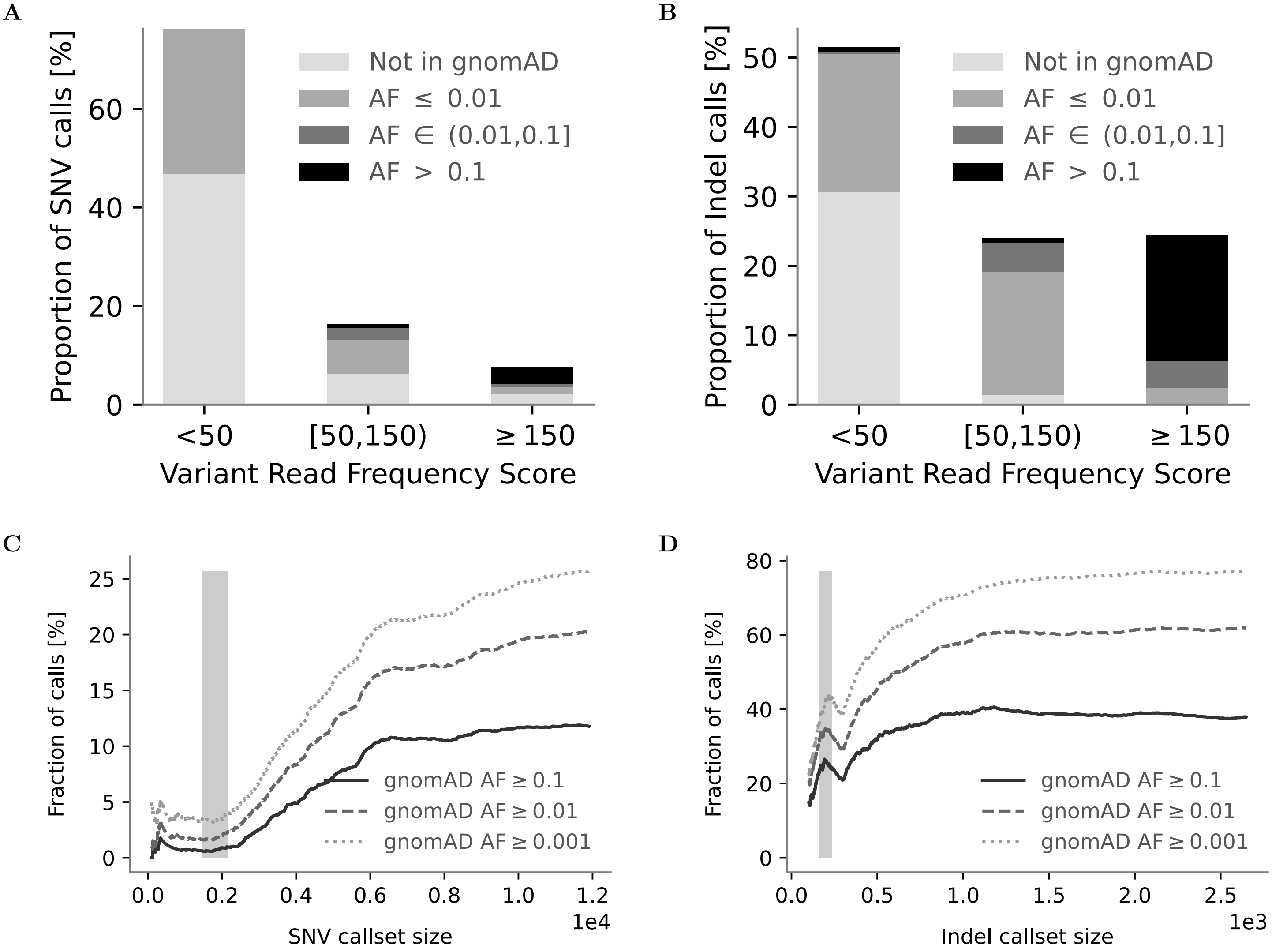

Supplementary Figure S16: A-B) Proportion of *de novo* SNV and indel sites in the clean callset observed at the indicated allele frequencies in gnomAD (see legend), stratified by Variant Read Frequency Score (VRFS). **C-D**) Fraction of sites in the raw callset observed at the indicated allele frequencies in gnomAD (AF≥0.1, AF≥0.01, and AF≥0.001), shown as a function of callset size. Candidate variants were ranked in descending order by DMM score, and the fraction of sites observed in gnomAD was computed cumulatively as progressively larger sets of top-scoring candidates were included. The grey bar denotes the expected approximate size of the true *de novo* callset. Sites were matched only by position, the specific alternate alleles were not taken into account.

S17 VRFS helps to identify misclassified inherited variation

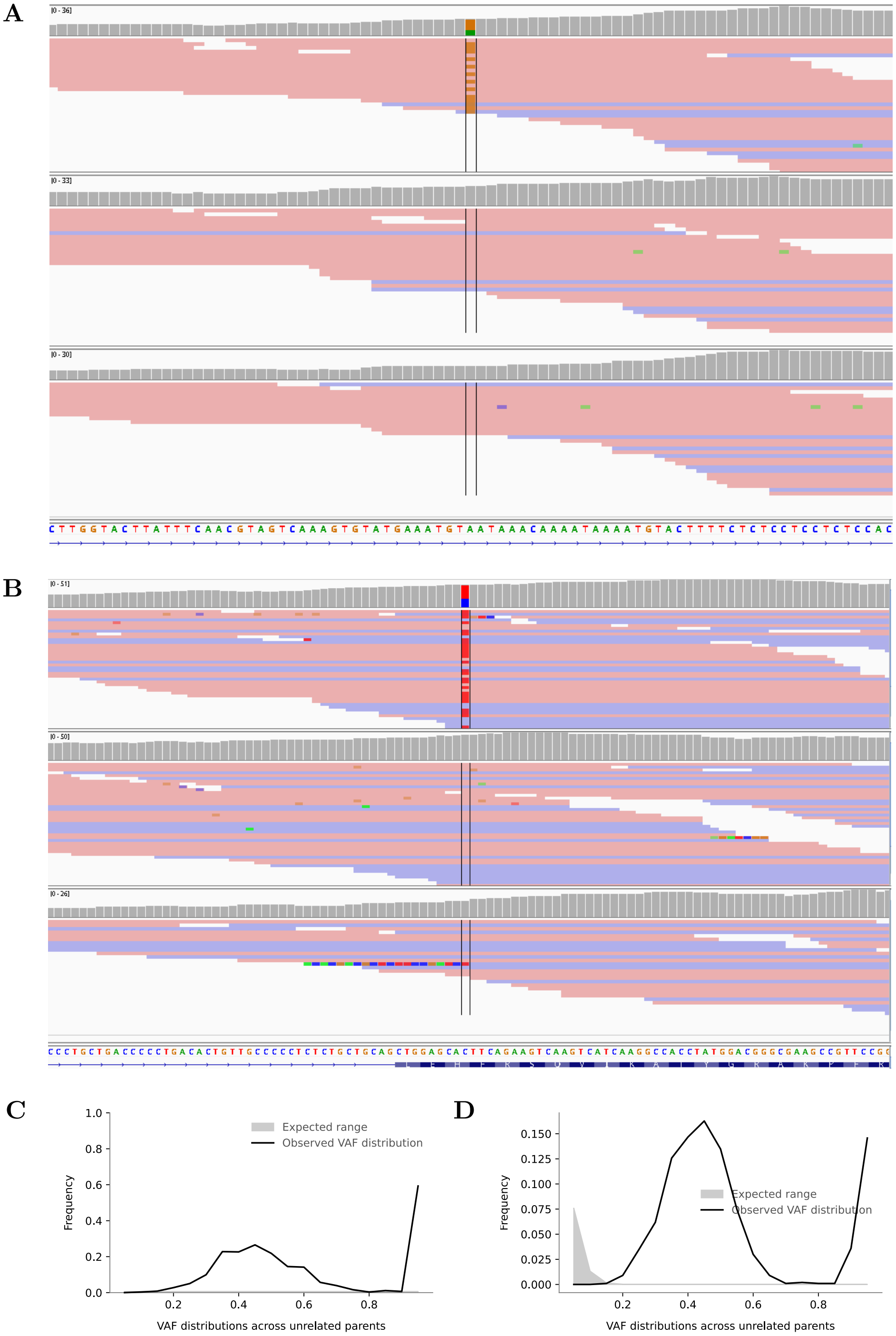

Supplementary Figure S17: Two examples of inherited variants, common in general population and in the BiB cohort, misclassified as *de novo* due to low sequencing depth in the parents. The IGV plots show the proband in the top lane, followed by father and mother. **A**,**C**) The A>G variant at chr4:118,255,548 is likely inherited from father (middle lane), the probability of missing the alternate allele with N=13 supporting reads is $p = 0.0002$. The variant is present in gnomAD with AF=0.41. **B**,**D**) The C>T variant at chr1:15,327,067 is likely inherited from mother (bottom lane). There are fewer usable reads than it seems, 10 out of the 15 reads form read-pair overlaps, reducing the effective sequencing depth to 10 reads. The probability of missing the alternate allele is $p = 0.002$ (N=10). The variant is present in gnomAD with AF=0.28.

S18 VRFS helps to identify false positives in difficult regions

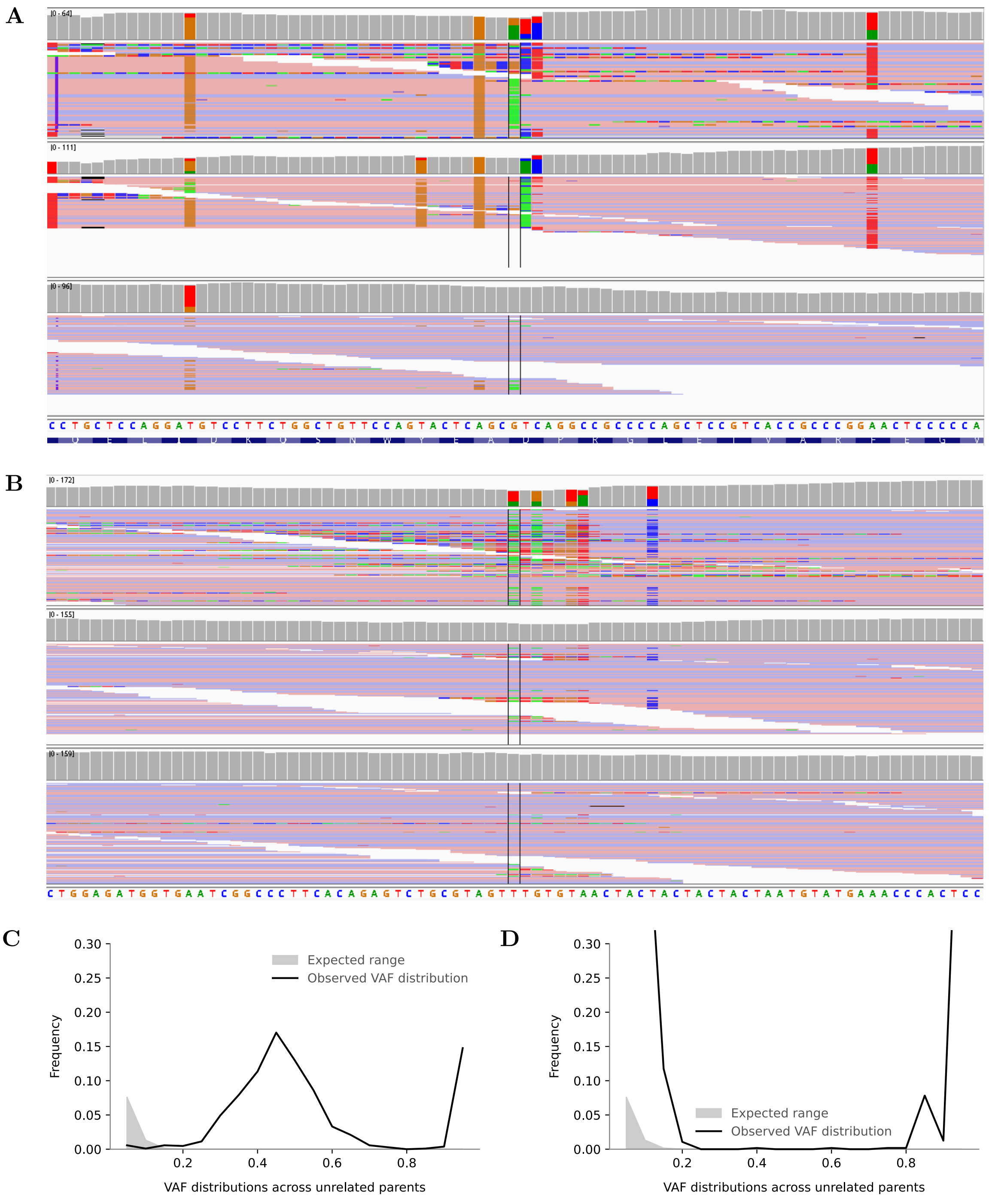

Supplementary Figure S18: Two examples of false positives, G>A at chr6:32,584,221 and T>A at chr14:106,116,756, with good TDNM scores, identified with the help of their variant read frequency score (**C-D**). The IGV plots show the proband in the top lane, followed by father and mother.

S19 List of recurrent *de novo* sites

List of recurrent sites (VRFS≥100) not observed in gnomAD v4.1, with coordinates on the GRCh38 reference build, ordered by decreasing VRFS. This set was derived from a single cohort and its generalizability to other datasets has not been evaluated. Moreover, some recurrent signals may reflect technical artefacts related to sequencing or data processing (e.g., sequencing chemistry, capture design, mapping, variant calling, batch effects, or sample contamination).

#[1]chr	[2]pos	[3]ref	[4]alt	[5]VRFS
chr8	12430555	G	A	226
chr20	45891601	G	C	218
chr17	64897339	T	A	206
chr4	109685238	C	T	204
chr2	95951446	A	G	203
chr3	10046558	G	A	203
chr3	10046555	G	A	202
chr7	26212223	C	T	202
chr7	99909729	G	T	199
chr3	75741517	G	T	197
chr7	112286841	C	T	194
chr5	132257643	C	G	193
chr11	4955538	C	T	192
chr16	55828678	G	T	191
chr7	142749631	C	T	191
chr17	15699636	G	T	189
chr2	95951291	A	C	189
chr14	106349187	G	A	187
chr21	10541004	A	G	187
chr8	100706893	G	A	187
chr3	75741432	G	A	186
chr6	31269347	T	C	185
chr1	161548424	T	C	183
chr21	37122970	A	T	182
chr3	75741403	C	T	182
chr7	142773602	G	A	182
chr7	150570017	T	G	181
chrX	136878071	C	T	180
chr17	15699717	G	T	179
chr14	55007231	T	A	178
chr16	55820561	T	C	177
chr22	23857075	G	T	176
chr15	23440317	A	T	174
chr21	10578264	T	C	174
chr5	180614276	C	T	174
chr1	19282943	T	C	173
chr1	161548546	C	T	172
chr7	142770925	C	G	171
chr8	97805331	T	G	170
chr9	33794929	T	C	170
chrX	154092116	T	A	170
chr3	75731964	A	G	168
chr7	56019781	A	G	168
chr5	177737043	G	A	166
chr1	10966324	T	A	165
chr3	10064924	T	C	162
chr9	133208307	G	C	162
chr2	90154560	T	C	161
chrX	147912081	A	C	161
chr2	232380380	A	C	160
chr16	89587172	C	T	159
chr19	327260	T	A	159
chr3	116444813	C	A	158
chr3	196803055	C	A	158
chr14	78810377	T	A	157
chr19	54596033	C	T	156
chr11	67991914	C	T	155
chr14	22947254	A	C	155
chrX	71132782	T	C	154
chr1	237742271	T	C	153
chr22	50267330	G	C	153
chr7	142770937	G	A	150
chr3	168037064	A	T	147
chr5	141194602	G	C	146
chr7	56021033	G	T	146
chr12	56612275	T	A	145
chr7	77167611	A	G	145
chr18	9122593	T	C	144
chrX	8465686	C	G	144
chrX	46879655	AT	A	144
chr17	41250240	T	C	143
chr19	54773430	A	G	143
chr13	19493000	T	G	141
chr1	35020471	T	A	140
chr5	134828616	G	T	140
chr6	32519367	C	T	140
chr2	240869074	T	A	139
chr5	79290304	T	G	139
chr6	10958939	A	C	138
chr6	31356518	C	A	137
chr1	21978560	C	T	136
chr10	29490860	C	T	136
chr11	46347752	C	G	136
chr1	32222058	A	C	133
chr2	232380151	G	C	133
chr9	70864542	G	A	133
chr13	24442715	C	T	131
chr9	135521625	A	C	130
chr18	9122613	T	C	129
chr19	54773575	A	C	129
chr12	80811419	G	A	128
chr6	17608351	A	G	128
chr9	135521622	G	C	128
chr16	20813351	T	C	126
chr22	23617565	G	A	125
chr6	29944621	A	G	125
chr17	68551122	T	G	123
chr19	21424723	A	G	122
chr19	21424724	A	G	121
chr1	12777650	C	T	120
chr10	32711802	T	G	118
chr9	107003503	T	G	118
chr1	153070875	A	G	116
chr12	42087790	T	C	116
chr11	56470088	A	T	114
chr11	95049392	T	G	114
chr2	213008132	A	T	114
chr9	4605516	T	G	114
chr19	4525883	C	T	113
chr4	108621975	A	C	112
chr17	58199826	T	G	109
chr13	19493005	T	C	108
chr17	74772363	A	C	107
chrX	70284229	T	G	107
chr10	29494927	C	T	106
chr6	27807455	A	C	105
chr12	109290043	A	C	104
chr14	105623000	T	C	104
chr19	54775527	G	T	104
chr3	10043954	C	T	104
chr20	13587244	A	C	102
chr3	170361464	T	G	102
chr12	103982830	G	A	101
chrX	141003743	G	C	101
chr10	71296789	G	A	100
chr2	27635298	C	CAATTTTTT	100

S20 The sensitivity of VRFS to sample size

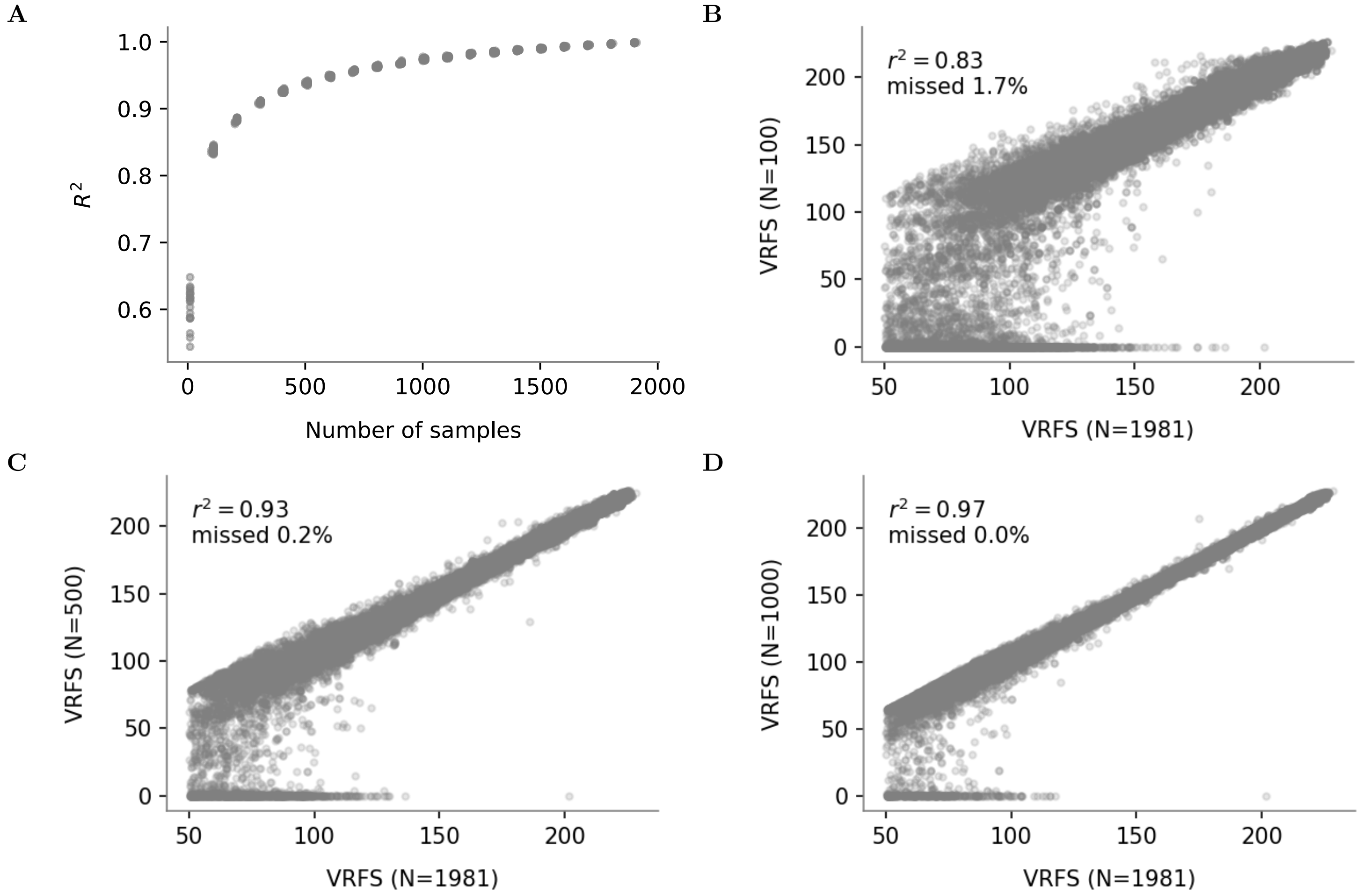

Supplementary Figure S20: A) The correlation of VRFS values calculated within the context of all samples (N=1981) and in random subsets of 0.5-96% samples (N=10 to 1901). **B-D**) The proportion of recurrent sites (VRFS≥100) reported as non-recurrent (VRFS<50) in a random subset of N=100, 500, and 991 samples.

S21 DNM filtering pipeline used for the BiB data

A real-world example of a DNM filtering pipeline follows. We used `bcftools` commands outlined in Supplement S2, as well as custom scripts accessible from `https://github.com/HurlesGroupSanger/trio-dnm-calling`.

Metrics used in filtering

CLUSTER_SIZE
: Number of variant sites within 60 bp window

DMM
: The TrioDNM3/DMM score

MQBZ
: Mann-Whitney U-Z test of Mapping Quality of supporting reads

NMBZ
: Mann-Whitney U-Z test of number of mismatches within supporting reads

nReads_proband, nrReads_mother, nReads_father
: The read coverage

SCBZ
: Mann-Whitney U-Z test of Soft-Clip Length Bias

VCF_QUAL
: The VCF QUAL column

VRFS
: The variant read frequency score

Filtering

The process of variant filtering is inherently somewhat arbitrary, representing a compromise between specificity and sensitivity. The selection of filtering thresholds is often guided by empirical observations and practical considerations rather than fixed rules, balancing the risk of excluding true variants against the inclusion of false positives. To aid in this filtering process, we developed the `run-filtering` pipeline that allows for the dynamic exploration of a range of thresholds simultaneously in an interactive manner. This enables us to identify the most informative predictors and determine optimal threshold values more effectively.

First, we pre-filtered the dataset with a lenient expression to reduce the size of the callset.

```
$DMM>-5 &&                       # A very lenient threshold, just to reduce the size of the callset
$VCF_QUAL>35 &&                  # Remove the least confident calls
$CLUSTER_SIZE<4 &&               # Remove calls with more than three variant sites within 60 bp window
$nReads_proband>=10 &&                             # Require at least 10x coverage in all trio samples,
$nReads_mother>=10 &&                              #   accounting for chrX in males which can be inherited
($nReads_father>=10 || $inheritance eq "mother")  #   only from mother
```

Next we used the `run-filtering` script to refine the filtering query and generate an interactive visualization of the callset to aid with finding the optimal thresholds

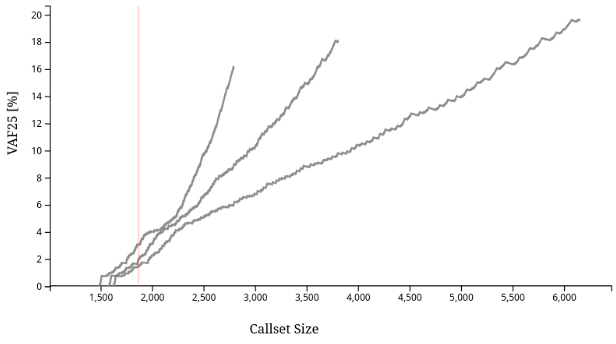

abs($MQBZ)<=4 && abs($NMBZ)<=4 && abs($SCBZ)<=4 && $VRFS<{ 50 , 100 , 200 }

Expression	Size	O/E Size	VAF25[%]	dup[%]	ti/tv	O/E syn	O/E mis	TPR[%]	Rank:DNM;VCF_QUAL
(1) abs($MQBZ)<=4 && abs($NMBZ)<=4 && abs($SCBZ)<=4 && $VRFS<50	1865	1.08	3.11	0.54	2.55	0.79_ 0.89 _1.00	1.24_ 1.33 _1.43	97.8	13904:-3.16227e-10;140.27
(2) abs($MQBZ)<=4 && abs($NMBZ)<=4 && abs($SCBZ)<=4 && $VRFS<100	1865	1.11	2.04	0.54	2.73	0.82_ 0.93 _1.04	1.28_ 1.37 _1.47	97.7	14144:-1.58451e-12;161.577
(3) abs($MQBZ)<=4 && abs($NMBZ)<=4 && abs($SCBZ)<=4 && $VRFS<200	1865	1.11	1.50	1.55	2.69	0.82_ 0.92 _1.04	1.28_ 1.37 _1.46	95.2	14276:-4.9738e-14;178.176

S22 Supporting data

Supplementary File `supporting-data.zip` contains the supporting data files used to generate the figures and supplementary analyses in this manuscript. These files do not include individual-level de novo mutation (DNM) data, which are subject to access controls under the project's data access agreement and are not publicly available. Access to individual-level DNM data may be requested through the Born in Bradford Executive, subject to approval.

`vrfs.txt.gz` .. VRFS output for 1,012,098 candidate DNMs identified from 1,981 BiB samples. This file was used to generate:

- Figure 2
- Supplementary Figure S3
- Supplementary Figure S6
- Supplement S19

`roc.trio-dnm.bib/` .. Data used to generate the ROC curves shown in Figure 3.

`roc.vrfs.bib/` .. Data used to generate the ROC curves shown in Figure 4.

`roc.trio-dnm.sim/` .. Data used to generate the ROC curves shown in Supplementary Figure S7.

`roc.trio-dnm.dp/` .. Data used to generate the ROC curves shown in Supplementary Figure S12.

`tpr-by-qual/` .. Data used to generate TPR and FPR by quality curves shown in Supplementary Figure S8.

`titv-by-size/` .. Data used to generate ti/tv by callset size shown in Supplementary Figure S9.

`vaf-by-size/` .. Data used to generate VAF25 by callset size shown in Supplementary Figure S10.

`vrfs-by-size/` .. Data used to generate recurrence by callset size shown in Supplementary Figure S13.

`vrfs-repeats/` .. Data used to generate repeat content by VRFS shown in Supplementary Figure S14.

`inherited/` .. Data used to generate missed inheritance graphs shown in Supplementary Figure S15.

`gnomad/` .. Data used to generate presence in gnomAD by AF and callset size shown in Supplementary Figure S16.

References

[Conrad *et al.*, 2011] Conrad D, *et al.* Variation in genome-wide mutation rates within and between human families *Nat Genet*, **43**, 712-714 (2011).

[Ramu *et al.*, 2013] Ramu A, *et al.* DeNovoGear: *de novo* indel and point mutation discovery and phasing, *Nat Methods*, **10(10)**, 985-987 (2013).

[Li, 2010] Li H, The revised MAQ model, (2010)
http://samtools.github.io/bcftools/samtools.pdf
