# Supplementary material for: Improved discovery of de novo mutations using TrioDNM and VRFS
Source: Gigascience. 2026 Jun 9;15:giag068. doi: 10.1093/gigascience/giag068 (PMC13289757; doi:10.1093/gigascience/giag068)
Supplement: giag068_GIGA-D-25-00258_original_submission [file giag068_giga-d-25-00258_original_submission.pdf]

GigaScience

TrioDNM and VRFS: de novo indel and point mutation discovery

--Manuscript Draft--

**Manuscript Number:**	GIGA-D-25-00258
**Full Title:**	TrioDNM and VRFS: de novo indel and point mutation discovery
**Article Type:**	Research
**Funding Information:**	Wellcome Trust (220540/Z/20/A) — Prof Matthew E Hurles<br>Wellcome Sanger Institute (Quinquennial Review 2021-2026) — Prof Matthew E Hurles
**Abstract:**	Background: Identifying de novo mutations (DNM) is an important component of both genetic research studies and clinical diagnostic workflows, but is complicated by distinguishing true mutations from sequencing errors. Likelihood-based error models are more accurate than inferring mutations from genotypes alone but the resulting callsets still have high false positive rates.<br>Results: We identify that the main source of false positive DNMs comes from the use of genotype likelihoods in an otherwise robust mutational model. To address this issue, we propose a method based on allelic likelihoods which builds on an existing DNM calling approach DeNovoGear, but with higher accuracy and no decrease in sensitivity. Furthermore, we explore a method which collects allele specific frequency profiles in the sequenced cohort from across many unrelated samples and identifies sites that either demonstrate high rates of sequencing and mapping errors, or are unlikely to be clinically significant due to their high recurrence rate.
**Corresponding Author:**	Petr Danecek<br>Wellcome Sanger Institute<br>Hinxton, UNITED KINGDOM
**Corresponding Author Secondary Information:**	
**Corresponding Author's Institution:**	Wellcome Sanger Institute
**Corresponding Author's Secondary Institution:**	
**First Author:**	Petr Danecek
**First Author Secondary Information:**	
**Order of Authors:**	Petr Danecek<br>Eugene J Gardner<br>Matthew E Hurles<br>Sarah J Lindsay
**Order of Authors Secondary Information:**	
**Additional Information:**	
**Question**	**Response**
Are you submitting this manuscript to a special series or article collection?	No
**Experimental design and statistics**<br><br>Full details of the experimental design and	Yes

Powered by Editorial Manager® and ProduXion Manager® from Aries Systems Corporation

statistical methods used should be given in the Methods section, as detailed in our Minimum Standards Reporting Checklist. Information essential to interpreting the data presented should be made available in the figure legends.<br><br>Have you included all the information requested in your manuscript?	
**Resources**<br><br>A description of all resources used, including antibodies, cell lines, animals and software tools, with enough information to allow them to be uniquely identified, should be included in the Methods section. Authors are strongly encouraged to cite Research Resource Identifiers (RRIDs) for antibodies, model organisms and tools, where possible.<br><br>Have you included the information requested as detailed in our Minimum Standards Reporting Checklist?	Yes
**Availability of data and materials**<br><br>All datasets and code on which the conclusions of the paper rely must be either included in your submission or deposited in publicly available repositories (where available and ethically appropriate), referencing such data using a unique identifier in the references and in the "Availability of Data and Materials" section of your manuscript.<br><br>Have you have met the above requirement as detailed in our Minimum Standards Reporting Checklist?	Yes
GigaScience has policies and guidelines in place for the use of generative AI-writing tools such as ChatGPT. If you have used such writing tools to assist with writing the manuscript this must be	No

declared and cited in the text. Authors should not list AI-writing tools and other AI-assisted technologies as an author or co-author and should acknowledge that they are fully responsible for text generated or refined by AI-writing tools.<p><br><br>A summary of use (particularly in the introduction or among methods) needs to be included at the end of the paper, and the outputs should also be included as a supplementary file hosted in GigaDB or other open repositories. Please read our guidelines for more information. <p><br><br>By submitting to GigaScience, you are aware of the journal's AI-writing tools policy, and if you have declared use of such tools below, you have acknowledged this where appropriate in your manuscript and have made a summary of use and outputs available. <p><br><b>AI-assisted writing tools have been used in the preparation of this manuscript?	

Manuscript Click here to access/download;Manuscript;main.pdf

Placeholder for OUP logo oup.pdf

Placeholder for journal logo gigascience-logo.pdf

GigaScience, 2024, 1–7

doi: xx.xxxx/xxxx
Manuscript in Preparation
Paper

PAPER

TrioDNM and VRFS: *de novo* indel and point mutation discovery

Petr Danecek[1]*, Eugene J Gardner[2], Matthew E Hurles[1] and Sarah J Lindsay[1]

[1]Wellcome Trust Sanger Institute, Wellcome Genome Campus, Hinxton CB10 1SA, UK and [2]MRC Epidemiology Unit, Wellcome-MRC Institute of Metabolic Science, University of Cambridge, Cambridge, UK

*pd3@sanger.ac.uk

Abstract

Background: Identifying *de novo* mutations (DNM) is an important component of both genetic research studies and clinical diagnostic workflows, but is complicated by distinguishing true mutations from sequencing errors. Likelihood-based error models are more accurate than inferring mutations from genotypes alone but the resulting callsets still have high false positive rates.
Results: We identify that the main source of false positive DNMs comes from the use of genotype likelihoods in an otherwise robust mutational model. To address this issue, we propose a method based on allelic likelihoods which builds on an existing DNM calling approach DeNovoGear, but with higher accuracy and no decrease in sensitivity.
Furthermore, we explore a method which collects allele specific frequency profiles in the sequenced cohort from across many unrelated samples and identifies sites that either demonstrate high rates of sequencing and mapping errors, or are unlikely to be clinically significant due to their high recurrence rate.

Key words: De novo mutation; DNM; bcftools

Introduction

De novo mutations (DNMs) are new genetic variants found only in the genome of the child and not in the genome of either biological parent. While a typical healthy human has ~60 DNMs of no known health consequence [1], DNMs are also an important source of morbidity among neurodevelopmental disorder (NDD) patients, with at least 31-40% of NDD patients having a DNM directly causing or contributing to their symptoms [2].

To identify DNMs from next generation sequencing data using parent-offspring trios, standard variant calling workflows are used [3]. In the most basic approach, resulting genotypes are then analysed to identify loci where only the child, and neither biological parent, have a heterozygous genotype. However, despite continuing advances in sequencing technology and variant calling protocols, genotyping errors occur at a much higher rate than true DNMs, which makes the detection of Mendelian inheritance violations at the genotype level impractical due to the high false positive rate. Therefore, sophisticated probabilistic models were developed which employed genotype likelihoods, transmission probabilities, and prior probability of observing a DNM [4, 5, 6, 7, 8]. Even though family-aware genotype likelihood-based methods are significantly more accurate than the basic genotype-based method [9], improvements can be made, which we highlight below.

There are two main modes of false positive DNM calls. First, true inherited variants in the child are falsely inferred to be DNMs due to being falsely called as being homozygous reference in the parents. Second, false variants in the child are called at sites that have unexpectedly high error rates.

The first issue is often linked to conflicting demands on sensitivity imposed on the input genotype information with respect to the presence of the alternate allele in the child and its absence in the parents. In the child, calling must be reliable yet appropriately selective, effectively preventing sporadic alternate reads caused by sequencing and mapping artifacts from being misinterpreted as genuine DNMs. Yet in the parents, calling must instead be oversensitive, and highlight the presence of alternate reads in the parents, to prevent an inherited variant from being mistaken for a DNM. These problems are frequently exacerbated by small variations in coverage, number of alternate reads, or mapping and base qualities,

Compiled on: July 1, 2025.
Draft manuscript prepared by the author.

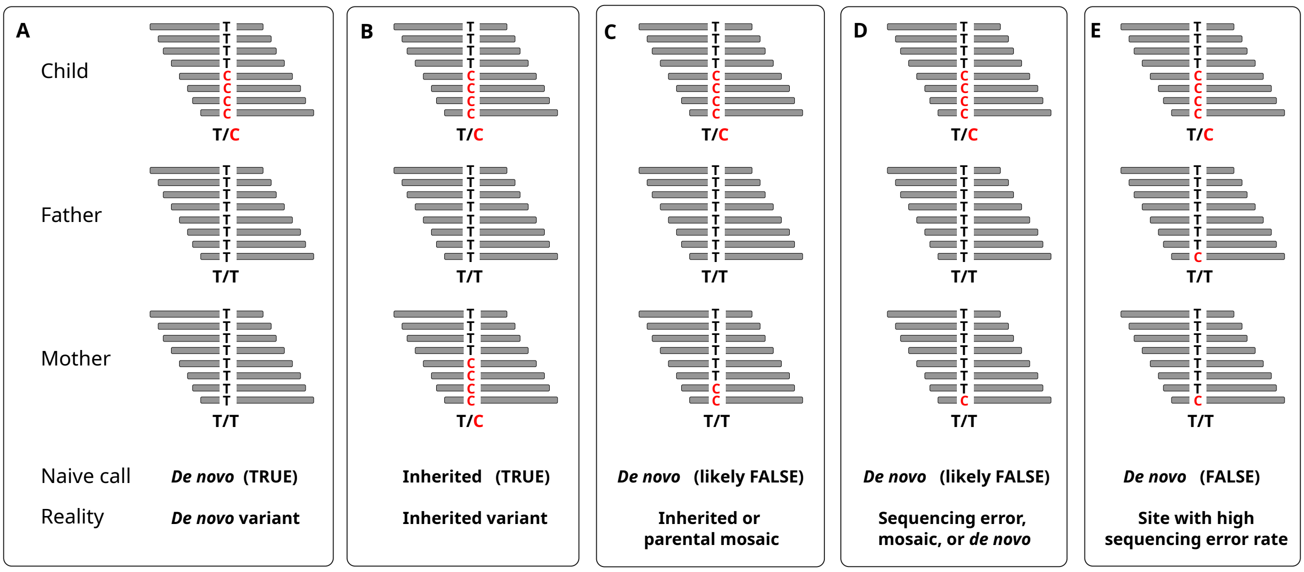

Figure 1. Schematic overview of the core problem of DNM calling. The candidate DNM has the same supporting evidence in the child, but different counts of alternate reads are observed in the parents. The genotypes below each case suggest the most likely genotype as determined by a germline variant caller. Panels **A** and **B** show cases where the ascertainment is trivial. Panels **C**-**E** show the problematic cases where one or both parents have the alternate allele, but the interpretation is uncertain. It can be a misclassified inherited variation **(C)**, sporadic sequencing errors **(D)**, or mapping artefacts **(E)**. Note that although the cases **C** and **D** can also have a valid biological reason, a mosaic post-zygotic parental mutation, the TrioDNM method is not able to distinguish between true parental mosaics and inherited sites where the proportion of the alternate allele in the parent deviates significantly from 50%.

and often lead to heterozygous call in the child but homozygous reference calls in both parents (Figure 1). Importantly, it is not sufficient to simply exclude sites which have an alternate read in the parents, because such an approach would not be able to distinguish between systematic errors, such as mapping and alignment artefacts, and random sequencing errors. As a result, it would lead to an elevated DNM false negative rate in the child.

Additional challenges include incorrectly classifying a site as a DNM due to an inherently noisy region with unusually high rate of base miscalls, or failing to detect the parental alternate allele due to insufficient sequencing depth. For example, assuming that reads with the alternate allele are sampled from the diploid genome following a binomial distribution, we estimate that approximately seven in a thousand heterozygous genotypes will appear as non-variant at 8x sequencing depth. Due to reference mapping bias, this estimate is conservative, particularly for indels, where the bias is more pronounced than for SNVs.

We introduce two methods which attempt to improve *de novo* calling: 1) we extended the genotype likelihood-based model implemented in DeNovoGear [9] to be more sensitive to alternate reads in parents by replacing genotype likelihoods with allelic likelihoods, and 2) we analyse the frequency of alternate reads at candidate *de novo* sites in healthy, unrelated parents from the same cohort which allow us to detect false variants at error-prone sites that are challenging to genotype, as well as true variants with high allele frequencies in the population.

Methods

The DeNovoGear model

The original DeNovoGear model [9] identifies DNMs by evaluating joint data likelihoods for all possible combinations of genotypes from mother, father and child (G_M, G_F, G_C) given the observed data (D) as follows

$$L(G_C, G_M, G_F|D) = P(D|G_C, G_M, G_F) \cdot P(P|G_M, G_F) \cdot P(G_M, G_F). \quad (1)$$

The first term in Equation 1 is the product of genotype likelihoods in the parent-offspring trio

$$P(D|G_C, G_M, G_F) = P(D|G_C) \cdot P(D|G_M) \cdot P(D|G_F) \quad (2)$$

and they are provided as input to the program. The second term in Equation 1 represents the transmission probability 0.25, 0.5, or 1 for genotypes compatible with Mendelian inheritance, modulated by a generic germline mutation rate $\mu = 10^{-8}$ for each novel allele. Finally, the third term is the prior probability of obtaining the two parental genotypes G_M and G_F from the population under the neutral coalescent model (Supplement S1).

The Equation 1 is evaluated for all possible combinations of genotypes and the most likely combination that is incompatible with Mendelian inheritance is selected. The posterior probability of the variant being a DNM is then calculated as

$$P(\mathrm{DNM}) = \frac{L(G_C, G_M, G_F|D)}{\sum_{c,m,f} L(c,m,f|D)}. \quad (3)$$

The summation in the denominator is over all possible genotype combinations, including combinations compatible with Mendelian inheritance.

This model has been reimplemented in the BCFtools/trio-dnm2 plugin and is accessible via the `-use-DNG` command line option (Supplements S1 and S2).

The TrioDNM model

The TrioDNM model aims to extend on DeNovoGear by increasing sensitivity to the presence of alternate reads in the parents. Specifically, genotype likelihoods were designed with robustness to sequencing and mapping errors in mind, but for the purpose of *de novo* calling, they are not sufficiently sensitive to rule out the presence of the alternate reads in the parents. If the proportion of alternate reads deviates significantly from 50%, the likelihood of a heterozygous genotype can become smaller than that of a homozygous genotype, even when the alternate allele is highly represented

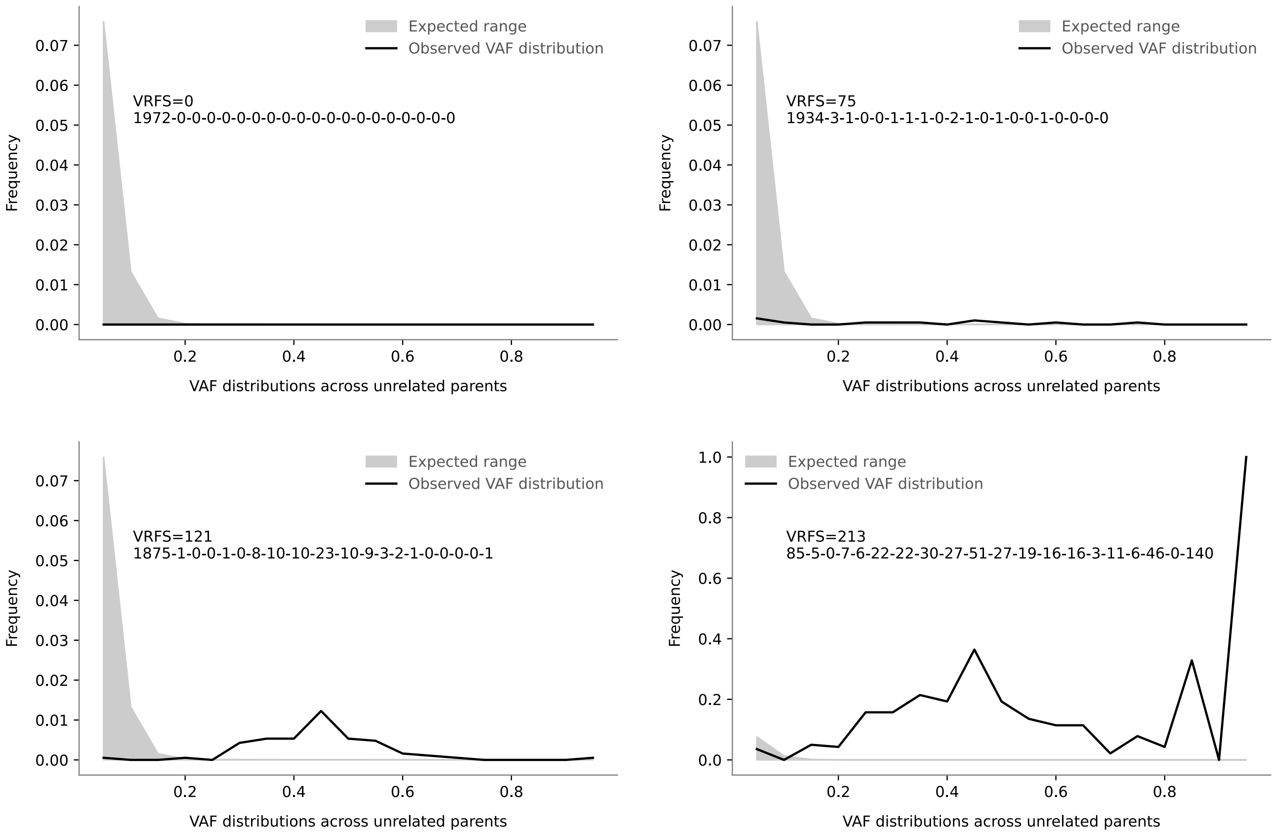

Figure 2. Examples of parental VAF distributions at four sites and the corresponding variant read frequency scores across a range of values, from VRFS=0 (alternate allele not present in the samples) to VRFS=215 (alternate allele is prevalent in the samples, with 140 having alternate homozgyous genotype).

in the parental reads. This can lead to misclassification of an inherited variant as a false DNM call.

In order to increase the sensitivity to alternate reads, we replace the parental genotype likelihoods in Equation 2 with parental allelic likelihoods:

$$P'(D|G_C, G_M, G_F) = P(D|G_C) \cdot A(D|G_{M,C}) \cdot A(D|G_{F,C}). \qquad (4)$$

By adding the subscript C, we emphasise that the parental likelihoods are no longer evaluated independently, but in the context of child genotype, as explained next.

For simplicity of notation, we limit the description to single nucleotide changes, but insertions and deletions are handled identically. We introduce a new variable Q_x which will represent the probability of all observed alleles $x \in \{A, C, G, T\}$ being genuine and not an artefact. Let $\epsilon_{x,i}$ denote the base error, defined here as the maximum of base and mapping error, of i-th read carrying the allele x. Then if the base x is present in reads covering a genomic position k_x times, the probability Q_x can be expressed as

$$Q_x = \begin{cases} 1 - \prod_{i=0}^{k_x} \epsilon_{x,i} & \text{if } k_x > 0, \\ 0 & \text{otherwise.} \end{cases} \qquad (5)$$

Note that the quantity Q_x is simply a sum of base/mapping qualities in log space and is calculated by BCFtools/mpileup when the `-annotate QS` option is present (Supplement S2). For true DNMs, the parental values Q_x should be small for the *de novo* allele x.

The allelic likelihood evaluated in the context of the parental base probabilities Q_x, paternal or maternal genotype $G = ab$ composed of alleles a and b, and the child genotype $G_C = cd$ is then calculated as

$$A(D|G_{ab,cd}) = \prod_x \begin{cases} Q_x & \text{if } x \in \{a, b\}, \\ 1 - Q_x & \text{if } x \in \{c, d\} \setminus \{a, b\}, \\ 1 - Q_x & \text{if } a = b \text{ and } x \notin \{a, b, c, d\}, \end{cases} \qquad (6)$$

where the multiplication is over all bases x observed at the position in the trio. The first case contributes with a large value when the alleles of the tested parental genotype ab have a strong presence in the parent; the second case penalises the presence of the child's alleles c, d in the parent when the tested combination is not compatible with Mendelian inheritance; the third case penalises the presence of multiple alleles when the tested parental genotype is homozygous.

The Variant Read Frequency Score

Studies of DNMs in large cohorts reveal that some candidate mutations, which initially appear genuine, are also found frequently in unrelated, healthy parents. To identify such sites, we developed a method that compares each candidate site against an expected noise profile. This expected profile is generated by collecting the proportions of alternate reads across a reference set of individuals (parental samples in our study) and across a set of high-confidence calls, as described below (Supplementary Figure S3). True DNMs are expected to have very few individuals in the reference set that have a high proportion of variant reads.

Let $(f_1, \ldots, f_k)$ be the distribution of the variant read proportions (also known as variant allele frequencies, VAFs) collected at a tested site across the reference set of individuals, using $k = 20$ bins by default (Supplementary Figure S4). We compute profile vari-

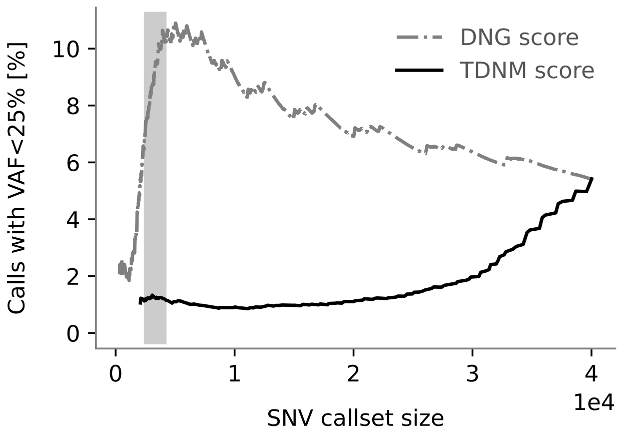

Figure 3. VAF25 by callset size. All candidate calls were sorted in descending order by one of the scores, DNG or TDNM, and at each threshold the VAF25 metric was calculated. On the left, the most strict filtering threshold was applied; on the right, all candidate sites were included. Lower values indicate a cleaner callset. The grey bar indicates the expected approximate size of the callset.

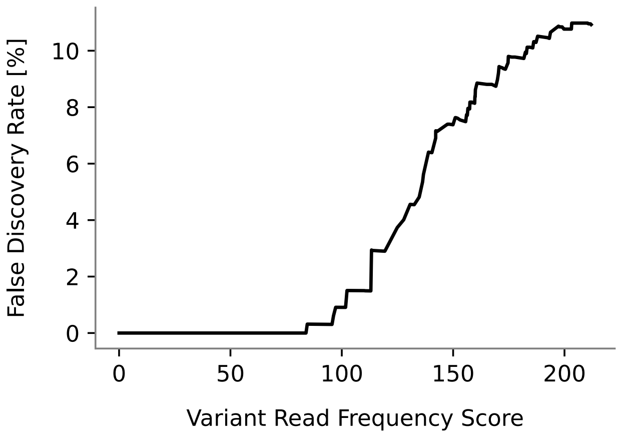

Figure 4. False discovery rate by VRFS. A clean set of 1,831 calls with the best TDNM score possible (TDNM=0) was sorted in ascending order by the VRFS value, and at each threshold the proportion of calls marked as false positive was calculated.

ances $\{\sigma_i\}$ for each distribution bin across a set of reference sites that have few individuals with a high proportion of variant reads (Supplementary Figures S3C and S5). The variant read frequency score (VRFS) of a site is then calculated as

$$\mathrm{VRFS}(f_1, \ldots, f_k) = 10 \log(1 + \sum_{i=2}^{k} f_i^2/\sigma_i^2). \qquad (7)$$

Note the first bin which corresponds to VAF=0 is not included in the summation. The calculation of the normal probability density function in the log-log space ensures scaling of VRFS values to the range of 0-250 and captures well the qualitative observation of recurrent (i.e. noisy) sites (Figure 2 and Supplementary Figure S6).

Putative DNMs with multiple alternate alleles pose a problem – while multiple alternate alleles observed frequently in unrelated samples is often a hallmark of mapping artefacts in difficult regions, there are also rare cases of genuine DNMs at polymorphic sites. We address this issue differently for SNVs and indels. For multiallelic SNVs, we create a VAF profile and calculate the VRFS value for each alternate allele x separately, then increase the scores towards the noisiest of the alleles m as follows

$$\mathrm{VRFS}'_x = \mathrm{VRFS}_x + 0.75(\mathrm{VRFS}_m - \mathrm{VRFS}_x). \qquad (8)$$

Because insertions and deletions are more difficult to genotype, prone to alignment errors and the read alignment often ambiguous, we treat all indels as biallelic regardless of alternate sequence(s).

A user-friendly, performative version of this model has been implemented in the BCFtools/vrfs plugin (Supplement S2).

Data Description

To compare DeNovoGear and TrioDNM, we used exome sequencing data generated for 1,094 trios (1,094 children and 1,981 parents) from the Born in Bradford study (BiB). See [10] for details on sample collection and exome sequencing. The Illumina NovaSeq 100bp paired-end reads were aligned to GRCh38 using BWA-MEM [11], the median of minimum sequencing depth per trio in target regions was 38x (Supplementary Figure S7).

Prefiltered candidate callset

Initial variant calls were made with GATK HaplotypeCaller v4.3.0.0 following GATK best practices [3] and raw candidate *de novo* callset was generated by selecting sites that do not conform to Mendelian inheritance using the naive function of the BCFtools/trio-dnm2 plugin (Supplement S2). The initial candidate callset consisted of 72,436 unique SNV and 38,285 indel sites. To reduce the size of the dataset for downstream processing, a lenient prefiltering step was applied, retaining only sites with a DeNovoGear or TrioDNM score (or both) greater than -8. The prefiltered candidate *de novo* callset consisted of 21,383 SNV and 9,379 indel sites.

Filtered callset

We next refined the prefiltered candidate set to eliminate the majority of false positives. It should be noted that filtering pipelines employed in real-world applications are typically more complex, incorporating additional criteria to optimize both specificity and sensitivity (Supplement S21). The callset described below is intended solely for the purpose of characterizing the properties of VRFS within a reasonably well-filtered dataset.

First we established a labelled truth set by inspecting in IGV [12] a random subset of 374 (1.2%) variants from the prefiltered candidate callset described above and marked 163 as true positive and 211 as false positive. Then, using these 374 manually curated sites we explored the typical TDNM and DNG scores of true and false calls: 93% of SNV calls marked as true had a TDNM score bigger than -2 and 89% had DNG score bigger than -4; further, 95% of indel calls had TDNM score bigger than -1e-4 and DNG score bigger than -4 (Supplementary Figure S8). Therefore, in the filtered set we required that both scores exceeded these thresholds. Additionally, in order to avoid inherited calls missed due to the stochastic nature of binomial sampling of reads from the diploid genome during sequencing, we required a minimum depth of 10x in all three samples, while accounting for inheritance patterns on the sex chromosomes.

The callset filtered in this manner contained a significant proportion of duplicate sites, with 16% of SNVs and 10% of indels appearing multiple times. These duplicates are likely artifacts rather than genuine DNMs; indeed, 97% of these calls were repeatedly observed among unrelated parents. To highlight the utility of the VRFS method after creating the cleanest possible callset using conventional approaches, we removed these duplicates as evident artifacts. This refinement resulted in a filtered callset comprising 3,474 SNVs and 285 indels, which we then used to further investigate the properties of VRFS.

Results

We evaluated DNM callsets generated by applying the methods to exome sequencing data from 1,094 parent-offspring trios, and explored the properties of VRFS.

TrioDNM enables more stringent filtering than DNG

The initial candidate callset made by GATK was annotated with the DeNovoGear (DNG) and TrioDNM (TDNM) scores using the commands shown in the Supplement S2. To compare the performance of the methods, we then used the VAF25 and ti/tv summary metrics. VAF25 is the proportion of calls with less than 25% reads supporting the alternate allele in the child and ti/tv is the ratio of nucleotide transitions to transversions. Both metrics show the TrioDNM method generates a score that has a greater potential to be used for filtering, and produces a cleaner callset at any given size, with more uniform VAF, fewer sequencing errors and approximating the expected size given the expected mutation rate (Figure 3 and Supplementary Figures S9-S11). To further evaluate each method, we annotated the set of 374 manually curated variants described above with TDNM and DNG scores, and calculated a false discovery rate per method. Overall, the false discovery rate (FDR) of TDNM was lower than that for DNG at comparable sensitivity, which suggests TDNM is a more effective approach (Supplementary Figure S12).

We note that the labelled truth set of variants used for FDR calculation was generated through manual inspection, which flagged as false positives the specific patterns that the TrioDNM method was designed to suppress, i.e., we could be labelling true DNMs as false due to our own implicit biases. Additionally, a favourable ti/tv only suggests true biological variation but does not specifically indicate *de novo* variation. On the other hand, the fraction of alternate reads in the child used by the VAF25 metric is applied by both DNG and TDNM methods identically; therefore, of the three metrics (FDR, ti/tv, and VAF25), only VAF25 can be considered a truly unbiased estimator of the *de novo* callset quality (Figure 3).

Compared to DNG, the TDNM score assigns a higher proportion of calls to the highest confidence bin. However, even though the TDNM score may be less nuanced in distinguishing between the highest quality calls, it produces a sufficiently small callset at its most stringent threshold, aligning with the estimated mutation rate of 2.0 - 3.9 $\cdot 10^{-8}$ calculated from data of 2,132 Icelandic families as published in the supplementary of [13]).

Confluence of TDNM and VRFS scores

Considering the size of the sequenced mutation target (~36.7 Mb across 1,094 individuals) and the *de novo* mutation rate (~10^{-8}), we do not expect mutations to occur at sites of common variation or to be shared among multiple individuals by chance, and these events are orders of magnitude less frequent than false positive *de novo* calls. The Allele Frequency Score (VRFS; Methods) offers a quantitative framework for identifying sites where the variant read is frequently observed in unrelated samples and thus, unlikely a true *de novo* event.

Such sites are likely to result from sequencing artifacts or, if they represent true recurrent DNMs that generate alleles common in the population, are unlikely to have clinical significance. It is reasonable to expect that calls identified as low quality by TDNM will often be also identified as low quality by VRFS.

After annotating all sites with VRFS, we observed that 91% of the prefiltered candidate SNV calls had VRFS>50, while in the filtered callset the fraction was 36% (Supplementary Figure S13). Consistent with our expectation, this indicates that low quality calls (as determined by the combination of the TDNM and DNG scores) are enriched for common variation and recurrent sequencing artefacts affecting many samples. A similar pattern was observed for indels; however, it was considerably less pronounced (98% vs. 81%), suggesting that TDNM and DNG scores are less effective at filtering indels and VRFS may have even greater potential to serve as a complementary filtering strategy for indels compared to SNVs.

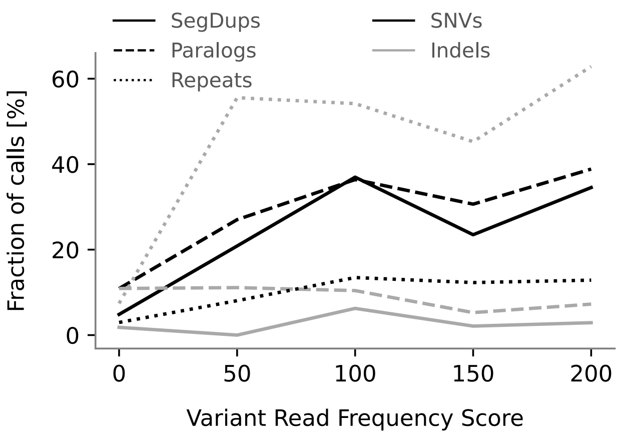

Figure 5. Repeat content by VRFS. The proportion of filtered calls found in segmental duplications, paralogs and various repeat types with 70% or higher sequence identity. The class denoted as "repeats" is predominantly populated with simple repeats (50%), SINE elements (25%) and low complexity regions (7%).

The same observation remains valid at a more granular level, within the filtered calls stratified by TDNM. Among the filtered SNV calls, sites we have labeled as low-recurrence based on VRFS value (VRFS≤50) were more likely to have high TDNM scores, forming 91% of the callset at the most stringent filtering TDNM threshold and 64% at its most lenient (Supplementary Figure S14). In contrast, low-recurrence sites comprised only 19% of the indel callset, while high-recurrence sites (VRFS≥150) accounted for 60% of the indel callset across all filtering thresholds (Supplementary Figures S14 and S15), suggesting again that TDNM is less effective at filtering indels and VRFS may be a useful filtering strategy.

Conversely, to assess accuracy across VRFS bins, we conducted manual inspection in IGV of 408 randomly selected candidate DNMs from the highest-quality bin (TDNM score = 0) (Figure 4). Even in this strictly filtered dataset we observed an increase of false positives with increasing VRFS values (VRFS≥100), up to FDR=11%.

Together, these results suggest that sites with large VRFS values or low TDNM scores are enriched for artefacts, but VRFS and TDNM scores are not correlated and provide largely complementary information. Therefore, filtering candidate DNMs using both scores is likely to yield a higher quality callset (see an example in Supplement S21).

Sources of recurrent candidate DNMs

We next sought to understand the genomic correlates of false positive DNMs. There are several potential reasons for observing apparent DNMs with high frequency. These include technical reasons such as instances of inherited variants that were mistakenly classified as *de novo*, or true biological effects (polymerase slippage, gene conversions, clonal expansions of mutant blood cells). We evaluate both here.

We anticipate that many recurrent *de novo* sites are within repetitive regions of the genome, leading to reduced alignment quality in short-read sequencing. Indeed, 50% of the highly recurrent SNVs and 58% of indels with VRFS≥150 from the filtered callset described above are found in paralogs, segmental duplications and repetitive regions (Supplementary Figure S15). SNVs and indels also differed in their error profiles. While recurrent *de novo* SNVs are observed predominantly in segmental duplications and paralogs, recurrent *de novo* indels more often occur in simple repeats and SINE/Alu elements (Figure 5).

We also investigated whether stochastic sampling bias in sequencing contributes to the misclassification of inherited variations

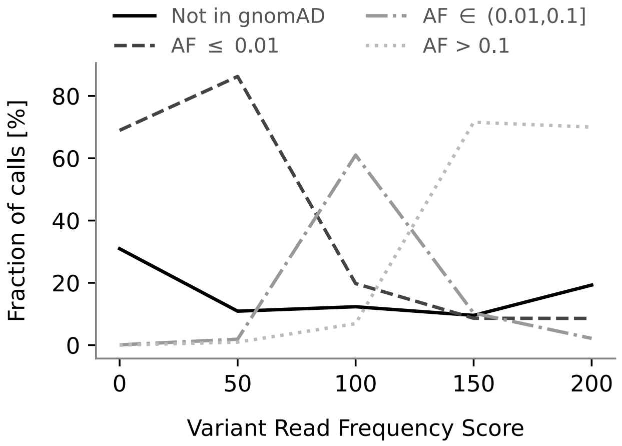

Figure 6. GnomAD sites by VRFS. The proportion of the filtered calls absent or present in gnomAD with allele frequencies as indicated in the legend. Sites were matched only by position, the specific alternate alleles were not taken into account.

as DNMs. We found that 14% of SNVs and 47% of indels have significantly higher probability of being misclassified as *de novo* because of not sampling the alternate allele in parents due to lower sequencing depth ($p \geq 10^{-4}$, binomial probability) and the prevalence of such sites strongly correlates with the VRFS value (Supplementary Section S16).

Regardless of the mechanism, it is reasonable to expect that both true common variants miscalled as reference allele in the reference samples and false variants miscalled due to high error rate sites, are also likely to be present in gnomAD [14]. Indeed, 77% of sites with a *de novo* SNV allele and 92% of sites with a *de novo* indel allele were found also in gnomAD as a polymorphic site, of which 30% and 76%, respectively, had allele frequency (AF) greater than 0.1 (Figure 6 and Supplementary Figure S17). The majority of these sites, with AF>0.1, were also observed in many unrelated samples in our study, i.e. had VRFS≥150.

While we observed that large VRFS values predominantly highlight misclassified inherited variation (such as in Supplementary Figure S18) and false positives in difficult-to-align regions (such as in Supplementary Figure S19), it is possible that highly recurrent somatic mutations could also be present among the candidate DNMs with high VRFS scores.

The stability of the VRFS method

A set of reference samples is required to generate VAF profile distributions. The sensitivity of the method to common variation and artefacts increases with the number of samples. Reducing the number of samples by 75% (from 1,981 to 500) reduces the sensitivity by 0.2% (i.e. 0.2% of recurrent sites with VRFS≥100 are newly reported as non-recurrent with VRFS<50) and limiting the number of samples by 95% (from 1,981 to 100) reduces the sensitivity by 1.7% (Supplementary Figure S20). This suggests the method can be used with relatively small cohorts – parental samples of as few as 50 trios can capture 98.7% of problematic sites.

Note that the method is also stable with respect to the bin size (by default $k = 20$) and the values remain highly correlated over a range of bins (Supplementary Figure S4).

Discussion

We developed two complementary methods to aid with calling and filtering of *de novo* SNVs and short indels. Both methods were implemented as standard BCFtools plugins with user-friendliness and performance in mind.

The first tool, TrioDNM, is designed for use with parent-offspring trios and addresses the limitations of existing methods. Specifically, prior methods rely on genotype likelihoods to assess the presence of the *de novo* allele in the parents. In contrast, our method leverages allelic likelihoods evaluated in the context of the child's genotype, achieving higher sensitivity while minimizing the false positive rate. We show that the TDNM score improves ranking of *de novo* variants and results in a more refined callset when compared to the DNG score, the previous version of the model.

The second method, VRFS, was developed for DNM calling but can have broader utility for other applications, e.g. generating high quality rare SNV/indel callsets in duos (a child plus one biological partent) and other non-trio data. The method collects allelic frequency profiles from many samples, enabling the filtering of sites with uncertain clinical relevance, such as misclassified inherited variants or sites that are difficult to genotype. Using the VRFS method, we found that 36% of apparent *de novo* SNVs and 81% of indels are also present as variants in unrelated samples. This observed recurrence is likely attributable to several factors, with distinct patterns for SNVs and short indels. Recurrent candidate *de novo* SNVs are more prevalent in segmental duplications and paralogous regions, while recurrent indels are significantly more common in simple repeats and SINE elements. It remains unclear whether these recurrences arise from technical artifacts–such as challenges in mapping short-read data to repetitive genome regions–or from true biological phenomena, such as polymerase slippage or gene conversions. Furthermore, the degree of recurrence strongly correlates with stochastic sampling bias, where lower sequencing depth in the parents increases the likelihood of missing the alternate allele.

The recommended approach for utilizing these methods is to rank candidate calls by the TDNM score, identifying variants that appear to be genuine DNMs within the parent-offspring trio. Subsequently, pooled information from multiple samples can be used to filter based on the VRFS value, effectively eliminating common artifacts and inherited variation. Note that a real-world filtering pipeline is typically more complex and incorporates additional filtering criteria (see an example in Supplement S21). The two methods presented here are specifically designed to address only a subset of the failure modes observed in sequencing data.

While public genomic resources like gnomAD could serve the same purpose for filtering out common variants, they cannot account for cohort-specific sequencing artifacts, or may not be available for the studied organism or reference build. Our findings show that relatively small sample sizes are sufficient to capture the majority variation–for instance, 100 samples could identify 98.7% of recurrent sites. Also, while the calculation of VRFS values is sensitive to VAF profile variances $\{\sigma_i\}$, which are precomputed from a set of high-confidence sites, we show that these error profiles can be reused between studies (Supplementary Figure S5).

The accuracy of the TrioDNM method is contingent upon the quality of its input data, namely genotype and allelic likelihoods. These inputs are frequently subject to inaccuracies, particularly in the case of indels, which are notoriously challenging to genotype accurately. However, the model could be improved–in theory both DeNovoGear and TrioDNM models could include allele specific mutation rates to account for known effects such as 5-Methylcytosine at a CpG being more prone to transition than unmethylated cytosine due to spontaneous deamination. Furthermore, while the VRFS method takes into account multiallelic SNVs individually, its treatment of indels is limited to a site-level assessment.

In general, the methods are not suitable for all forms of genetic variation, such as copy number variation (CNVs). Even though the genotype and allelic likelihoods, which serve as input data to the model, could be in principle provided for any variant type, in practice their accuracy is not sufficient to produce reliable results. Furthermore, while TrioDNM can detect DNMs at common polymorphic sites in the population which modify the alternate allele to reference (i.e. 0/1 to 0/0), the VRFS method will recognize such

sites as common polymorphism and a site that falls in this category will receive a high VRFS score.

Availability of Supporting Source Code

The software is available free of charge under the MIT license and can be accessed at `https://github.com/samtools/bcftools` and `https://github.com/HurlesGroupSanger/trio-dnm-calling`. See also Supplements S2 and S21.

Acknowledgements

This work was supported by Wellcome Grant reference number 220540/Z/20/A, Wellcome Sanger Institute Quinquennial Review 2021-2026. For the purpose of Open Access, the author has applied a CC BY public copyright license to any Author Accepted Manuscript version arising from this submission

This study makes use of data from the Born in Bradford programme, which is only possible because of the enthusiasm and commitment of the children and parents in BiB. We are grateful to all the participants, health professionals, schools and researchers who have made Born in Bradford happen.

EJG is an employee of and holds shares in Insmed, Inc.

References

1. Kaplanis J, *et al.* Genetic and chemotherapeutic influences on germline hypermutation. Nature 2022;605:503–508.
2. Wright C, *et al.* Genomic Diagnosis of Rare Pediatric Disease in the United Kingdom and Ireland. N Engl J Med 2023;388(17):1559–1571.
3. van der Auwera G, O'Connor BD. Genomics in the Cloud: Using Docker, GATK, and WDL in Terra. O'Reilly Media, Incorporated; 2020.
4. Ramu A, *et al.* DeNovoGear: de novo indel and point mutation discovery and phasing. Nat Methods 2013;10(10):985–7.
5. Wei Q, Zhan X, Zhong X, Liu Y, Han Y, Chen W, et al. A Bayesian framework for de novo mutation calling in parents-offspring trios. Bioinformatics 2014 12;31(9):1375–1381.
6. Francioli LC, *et al.* A framework for the detection of *de novo* mutations in family-based sequencing data. Eur J Hum Genet 2017;25:227–233.
7. Kolesnikov A, Goel S, Nattestad M, Yun T, Baid G, Yang H, et al. DeepTrio: Variant Calling in Families Using Deep Learning. bioRxiv 2021;.
8. Khazeeva G, Sablauskas K, van der Sanden B, Steyaert W, Kwint M, Rots D, et al. DeNovoCNN: a deep learning approach to de novo variant calling in next generation sequencing data. Nucleic Acids Research 2022 06;50(17):e97–e97.
9. Conrad DF, *et al.* Variation in genome-wide mutation rates within and between human families. Nat Genet 2011;43(7):712–714.
10. Koko M, Fabian L, *et al.* Exome sequencing of UK birth cohorts. Wellcome Open Res 2024;9:390.
11. Li H. Aligning sequence reads, clone sequences and assembly contigs with BWA-MEM. ArXiv 2013;26 May.
12. Thorvaldsdóttir H, *et al.* Integrative Genomics Viewer (IGV): high-performance genomics data visualization and exploration. Briefings in bioinformatics 2013;14,2:178–192.
13. Gunnar Palsson HJ*et al* Marteinn T Hardarson. Complete human recombination maps. Nature 2025;639:700–707.
14. Chen S, Francioli LC, Goodrich JK, *et al.* A genomic mutational constraint map using variation in 76,156 human genomes. Nature 2024;625:92–100.

Click here to access/download
Supplementary Material
supplement.pdf
