# Supplementary material for: Improved discovery of de novo mutations using TrioDNM and VRFS
Source: Gigascience. 2026 Jun 9;15:giag068. doi: 10.1093/gigascience/giag068 (PMC13289757; doi:10.1093/gigascience/giag068)
Supplement: giag068_GIGA-D-25-00258_revision_1 [file giag068_giga-d-25-00258_revision_1.pdf]

GigaScience

Improved discovery of de novo mutations using TrioDNM and VRFS

--Manuscript Draft--

**Manuscript Number:**	GIGA-D-25-00258R1
**Full Title:**	Improved discovery of de novo mutations using TrioDNM and VRFS
**Article Type:**	Research
**Funding Information:**	Wellcome Trust (220540/Z/20/A) — Prof Matthew E Hurles<br>Wellcome Sanger Institute (Quinquennial Review 2021-2026) — Prof Matthew E Hurles
**Abstract:**	Background: Identifying de novo mutations (DNM) is an important component of both genetic research studies and clinical<br><br>diagnostic workflows, but is complicated by distinguishing true mutations from sequencing errors. Likelihood-based error models<br><br>are more accurate than inferring mutations from genotypes alone but the resulting callsets still have high false positive rates.<br><br>Results: We identify that the main source of false positive DNMs comes from the use of genotype likelihoods in an otherwise<br><br>robust mutational model. To address this issue, we propose two alternative methods which build on an existing DNM calling<br><br>approach DeNovoGear, but with higher accuracy and no decrease in sensitivity.<br><br>Furthermore, we developed a method which collects allele specific frequency profiles in the sequenced cohort from across many<br><br>unrelated samples and identifies sites that either demonstrate high rates of sequencing and mapping errors, or are unlikely to be<br><br>clinically significant due to their high recurrence rate.
**Corresponding Author:**	Petr Danecek<br>Wellcome Sanger Institute<br>Hinxton, UNITED KINGDOM
**Corresponding Author Secondary Information:**	
**Corresponding Author's Institution:**	Wellcome Sanger Institute
**Corresponding Author's Secondary Institution:**	
**First Author:**	Petr Danecek
**First Author Secondary Information:**	
**Order of Authors:**	Petr Danecek<br>Eugene J Gardner<br>Joanna Kaplanis<br>Matthew E Hurles<br>Sarah J Lindsay
**Order of Authors Secondary Information:**	

Powered by Editorial Manager® and ProduXion Manager® from Aries Systems Corporation

Response to Reviewers:	We thank the reviewers for their helpful comments and suggestions. In response, we have revised and significantly extended the methods, analyses, and the manuscript. Detailed responses to each comment are provided below.<br><br>Reviewer #1: De novo mutations play a crucial role in genetic and trait evolution. Danecek et al. introduced two methods to enhance de novo mutation (DNM) calling and filtering. Generally, this paper is well-organized and written, presenting valuable methods for improving DNM calling.<br>I have a few suggestions or concerns that may help strengthen the work:<br><br>1.It was observed that the sequencing depth distribution in the BiB cohort exhibits two peaks (~13-fold, ~53-fold; Supplementary Fig. S7). Therefore, it is recommended that the authors evaluate the impact of sequencing depth on filtering performance.<br><br>We evaluated the impact of sequencing depth on filtering performance and added a new section, "The impact of sequencing depth on performance," where this analysis is presented.<br><br>2.All analyses were based on exome sequencing data (~37-Mb target regions). However, whole-genome sequencing (WGS), typically using next-generation sequencing (NGS), has become a common approach for profiling de novo mutations. If high-quality public trio NGS data are available, it is suggested that the performance of TrioDNM and VRFS in non-coding regions be assessed using WGS data.<br><br>Evaluating performance using WGS data would indeed be valuable. While we don't expect the methods to perform differently in coding and non-coding regions, we extended our analysis to include simulated WGS data, where the status of each variant is known and method performance therefore can be assessed in a controlled manner across the genome.<br><br>3.There is confusion regarding the call sets evaluated in different figures and supplementary figures. In the methods section, an initial call set (72k SNVs and 38k InDels), a prefiltered call set (21k SNVs and 9k InDels), and a manually curated call set were mentioned. It remains unclear whether the call sets used in Fig. 3 and Fig. S9 are the initial call sets. Additionally, Fig. S10 displays 60k InDels, which is inconsistent with the initial call set containing only 38k InDels. Thus, the authors are advised to supplement more detailed legend information for the figures.<br><br>Thank you for noticing this inconsistency which was indeed confusing. To resolve this, we simplified the analysis and regenerated the callsets from scratch, which also enabled evaluation of an updated version of the method motivated by comments from Reviewer #2. All affected figures and legends have been revised to clearly indicate the call sets used.<br><br>4.This study evaluated recurrent de novo sites with high frequency, which may be misclassified. These site coordinates are of great significance for research on human de novo mutations in genetics and medical diagnosis. It is suggested that the authors deposit this dataset as a supplementary table or in a public repository.<br><br>We have included a subset of highly recurrent sites identified in this study as a supplementary table for reference.<br><br>5.It is recommended that the authors add usage examples, such as those shown in Supplementary S2, to the GitHub homepage.<br><br>We have added a link to the documentation, which includes usage examples, to the GitHub homepage.

Powered by Editorial Manager® and ProduXion Manager® from Aries Systems Corporation

minor:
1.Regarding the title: The core of this study lies in the development of two complementary approaches for de novo mutation filtering. I think current title can not reflect the key points Alternative titles could be considered for reference, such as "Quality control of de novo mutation discovery using TrioDNM and VRFS" or "Refining de novo mutations using TrioDNM and VRFS".

Thank you for your suggestion. We have changed the title to “Improved discovery of de novo mutations using TrioDNM and VRFS”, which we agree is more informative and accurately reflects the scope of the manuscript.

2.In the data description: the notation "38x" should be standardized to "38×" or "38-fold". This correction should also be applied to Supplementary Fig. S7 and other related statements.

This is corrected, thank you.

3.Maximum value displayed in Fig 2 is 213, while the caption indicates 215.

This is corrected, thank you.

Reviewer #2: Danecek et al. propose two new methods to improve de novo mutation calling. The first method, allelic likelihoods, makes calling more sensitive to alternative reads in the parents. The second method, VRFS, attempts to identifying sites that are challenging to genotype, reducing false positive calls.

While I think that both proposed methods are important developments for the field, I often found the paper hard to follow as there were many sections that were missing details and justifications. I often found myself struggling to understand how results were generated and how I should understand them. The paper would be improved if the authors spent a bit more time clarifying its technical aspects both in the text and in the figures and figure legends.

STRENGTHS

Calling denovo mutations (DNMs) is a genomic and bioinformatic challenge because many genomic error rates are often at the same or greater magnitude as the rate at which DNMs occur. The authors correctly explain that the biggest sources of false positive DNM calls originate from calling a heterozygous parent as homozygous due to low coverage and calling a homozygous child as heterozygous due to contamination or difficult to align locations.

In response to these challenges, the authors have developed two novel methods to identify denovo mutations and filter out potentially problematic sites. The new methods are statistical in nature which is important because statistical approaches for finding denovo mutations have long been superior to more commonly used ad hoc and heuristic approaches.

The new methods were tested on genomic data from the Born-in-Bradford study using both a manually curated truth dataset as well as biological signals to demonstrate the quality of their approaches. The results clearly show that the new methods are an important contribution to the field.

Since generating large, high-quality truth datasets using real data is difficult and time consuming, the authors also looked at whether the output of their methods made sense biologically. This is a powerful technique for validating methods that is often overlooked.

WEAKNESSES

As mentioned above, the biggest weakness in this paper is the lack of clarity about the

technical aspects. I noticed several typos and omissions in the equations. Since this is a methods paper, the description of the methods should be well polished and unfortunately they are not at this time.

We have carefully revised and extended the methodological description throughout the manuscript to improve clarity and completeness. In particular, we corrected typographical errors in the equations and clarified several technical details.

The methods are validated on a small truth set of 374 manually curated variants. This seems to me to be a really small number and as the authors admit, potentially biased. For reasons such as these, most new methods are evaluated on both simulated and empirical datasets. While no simulated data is as messy as real data, simulations are still useful for evaluating methods. The paper would be improved if the authors tested their methods on a simulated dataset in which the truth is known. This could be accomplished by completely simulating data or introducing simulated mutations into their dataset. They could also down-sample high-quality validated datasets like Platinum Genomes to validate their methods.

We have extended the curated truth set to more than 5,000 sites, substantially increasing the number of manually evaluated variants. In addition, we now include analyses based on simulated data to further assess method performance when the ground truth is known. We also considered using Platinum Genomes; however, this dataset contains only a small number of trios and the variants were not called jointly within families, which limits its suitability for evaluating trio-based de novo calling methods.

TrioDNM model

The quantity Qx where `x` is {A, C, G, or T} estimates the probability that at **least one base call of `x` is not an error**. This is then used in various ways to calculate allelic likelihoods. This is a big change from using a multinomial or Dirichlet-multinomial distribution for genotype likelihoods, and I think it deserves more discussion and justification than is currently in the paper.

We have revised this section to clarify the description of the model and to provide additional intuition for its formulation. We also simplified the formulation of the model. Furthermore, inspired by the reviewer’s comment, in addition we implemented a Dirichlet–multinomial (DM) model and included it in the analyses. While this model required additional considerations in implementation, it addresses an important limitation of the original TrioDNM model—its overconfidence at low parental depth. In fact, it is now used as the default calling method.

Equation (6). This equation does not explain what happens when a != b and x is not in {a,b,c,d}. From looking at the source code it appears that it uses `1` for this final case. This should produce some weird likelihoods such that the probability of Dm = {A, A, A, A} given Gm = {C, G} and Gc = {G, G} is higher than Dm = {A, A, A, A} given Gm = {A, A} and Gc = {G, G}. While this may not matter when identifying the most likely mutation pattern, it should definitely affect how the calculation of equation (3). If the authors are using a trick to avoid this, they should clarify that in the paper.

Thank you for noticing. We have revised and fixed this issue.

VFRS

I have no statistical institution about what VRFS represents. I don't understand how the f_i's or sigma_i's were calculated or why they are squared. I also don't understand why there is a `1 +` in the calculation as well. As such, I have a hard time understanding how VRFS should work. The authors should include more motivation and explanation for the VRFS statistic.

We have expanded the description of the VRFS statistic to provide additional motivation and intuition for the formulation. In particular, we clarified how the f_i and sigma_i values are computed and added an explanation of the rationale for the squared terms and the 1+ term in the formula.

Other Weaknesses

The DNG and TDNM scores are not defined, which makes interpreting the results difficult. They appear to be log-probabilities, but the paper also describes them as bins. The authors need to clarify how the scores are calculated and used.

The program allows selecting how these scores are reported using the --dnm-tag option: as probabilities (0–1; float), log-scaled values (−∞ to 0; float), or Phred-scaled values (0–255; integer). By default, log-scaled values are used, as they are the most practical representation for the typical range of scores.

The manuscript does not describe how DNG and TDNM models were parameterized. The authors should discuss whether changing the values of parameters used in DNG and TDNM models would change the results of the model.

We added description of parameters and their values where appropriate.

The paper claims that VRFS and TDNM scores are not correlated and provide complementary information. I am not sure how this conclusion was made. The authors could create a scatter plot of TDNM vs VRFS to justify this claim.

We agree that the original wording was unclear. Our intention was not to make a claim about statistical correlation of VRFS and TrioDNM, but rather that they capture different types of signal: VRFS reflects the recurrence of variant reads across unrelated samples, while TrioDNM evaluates the likelihood of a variant under a trio inheritance model. Because they rely on different information, they can flag artefacts through distinct mechanisms and therefore provide complementary information. We have revised the text to clarify this point.

OTHER COMMENTS

Equation (1). This equation contains a typo. It should be

`L(Gc, Gm, Gf | D) = P(D | Gc, Gm, Gf) x P(Gc | Gm, Gf) x P(Gm, Gf)`

where the second term on the right has the child's genotype in it.

The reviewer is correct; this was a typographical error. Thank you for noticing. The equation has now been corrected in the manuscript.

Equation (3). The authors describe this as the posterior probability of the variant being a denovo mutation. This is not accurate. Equation (3) is the probability of the most likely genotype combination that is incompatible with Mendelian inheritance (given observed data). The probability that there is a denovo mutation in the child given data is
P(DNM | D) = sum(P(DNM | D, Gc, Gm, Gf) * P(D, Gc, Gm, Gf)) / sum(P(D, Gc, Gm, Gf))
where the numerator is also calculated by summing over possible genotype combinations. For example, DeNovoGear's `call` algorithm calculates this statistical accurately while it's `dnm` algorithm generates one like in this paper.
And finally, I think that the probability of a variant being a DNM (as used in the text)

	would be calculated by fixing the genotype of the child and summing over the parents.<br>P(DNM | D, Gc = g) = sum(P(DNM | D, Gc = g, Gm, Gf) * P(D, Gc = g, Gm, Gf)) / sum(P(D, Gc = g, Gm, Gf))<br>To be clear, there is nothing wrong about Equation (3); however, the text doesn't accurately describe it.<br><br>We agree that the original description was imprecise. The text has been revised to clarify that the probability of a variant being a de novo mutation is approximated by the posterior probability of the most likely genotype combination that is incompatible with Mendelian inheritance. The revised text now reads:<br>The probability of a variant being a de novo mutation is approximated by the posterior probability of the most likely genotype combination that is incompatible with Mendelian inheritance, as follows: [...]<br><br><br><br>Equation (5). The quantity Qx is described as the "sum of base/mapping qualities in log space". This is not correct. The relationship between Qx and the sum of base/mapping qualities (QS[x]) is Qx = 1 - 10^(-QS[x]/10). This should be corrected, or replaced by a statement that Qx can be calculated from QS.<br><br>We agree that our verbal description was confusing. We have revised the text to clarify the definition. In our formulation, ε_i denotes the probability of an error for the i-th read. In practice, we obtain ε_i from phred-scaled base or mapping quality as ε = 10^(-BQ/10). The Equation 5 now correctly states Qx = 1 - \prod ε_i. Intuitively, even a single high-quality supporting reads (small ε_i) is sufficient to make Qx large.<br>We have clarified in the text that although the practical calculation is performed in log space to prevent numerical underflow, the formulation presented in the manuscript uses plain probabilities for clarity.<br><br>Equation (6). I believe that only the reads from a mother or father are used to calculate Qx. The authors should clarify this by using notation for individual specific datasets in the paper. For example, if D = {Dm, Df, Dc}, then equation (6) could be written as A(Dm | Gm = ab, Gc = cd) for the mother's allelic likelihood.<br><br>During the revision, we realized that the original formulation was unnecessarily complicated. We therefore simplified and clarified the text.
**Additional Information:**	
**Question**	**Response**
Are you submitting this manuscript to a special series or article collection?	No
**Experimental design and statistics**<br><br><br>Full details of the experimental design and statistical methods used should be given in the Methods section, as detailed in our Minimum Standards Reporting Checklist. Information essential to interpreting the data presented should be made available in the figure legends.<br><br><br>Have you included all the information requested in your manuscript?	Yes
**Resources**	Yes

A description of all resources used, including antibodies, cell lines, animals and software tools, with enough information to allow them to be uniquely identified, should be included in the Methods section. Authors are strongly encouraged to cite Research Resource Identifiers (RRIDs) for antibodies, model organisms and tools, where possible.<br><br>Have you included the information requested as detailed in our Minimum Standards Reporting Checklist?	
**Availability of data and materials**<br><br>All datasets and code on which the conclusions of the paper rely must be either included in your submission or deposited in publicly available repositories (where available and ethically appropriate), referencing such data using a unique identifier in the references and in the "Availability of Data and Materials" section of your manuscript.<br><br>Have you have met the above requirement as detailed in our Minimum Standards Reporting Checklist?	Yes
GigaScience has policies and guidelines in place for the use of generative AI-writing tools such as ChatGPT. If you have used such writing tools to assist with writing the manuscript this must be declared and cited in the text. Authors should not list AI-writing tools and other AI-assisted technologies as an author or co-author and should acknowledge that they are fully responsible for text generated or refined by AI-writing tools.<p><br><br>A summary of use (particularly in the introduction or among methods) needs to be included at the end of the paper, and the outputs should also be included as a supplementary file hosted in GigaDB or other open repositories. Please <a href=https://academic.oup.com/gigascienc	No

e/pages/editorial_policies_and_reporting_standards target="_new" > read our guidelines for more information. <p><br><br>By submitting to GigaScience, you are aware of the journal's AI-writing tools policy, and if you have declared use of such tools below, you have acknowledged this where appropriate in your manuscript and have made a summary of use and outputs available. <p><br><b>AI-assisted writing tools have been used in the preparation of this manuscript?	

Powered by Editorial Manager® and ProduXion Manager® from Aries Systems Corporation

Manuscript Click here to access/download;Manuscript;main.pdf

Placeholder for OUP logo oup.pdf

Placeholder for journal logo gigascience-logo.pdf

GigaScience, 2024, 1–8

doi: xx.xxxx/xxxx
Manuscript in Preparation
Paper

PAPER

Improved discovery of *de novo* mutations using TrioDNM and VRFS

Petr Danecek[1*], Eugene J Gardner[2], Joanna Kaplanis[1], Matthew E Hurles[1] and Sarah J Lindsay[1]

[1]Wellcome Trust Sanger Institute, Wellcome Genome Campus, Hinxton CB10 1SA, UK and [2]MRC Epidemiology Unit, Wellcome-MRC Institute of Metabolic Science, University of Cambridge, Cambridge, UK

*pd3@sanger.ac.uk

Abstract

Background: Identifying *de novo* mutations (DNM) is an important component of both genetic research studies and clinical diagnostic workflows, but is complicated by distinguishing true mutations from sequencing errors. Likelihood-based error models are more accurate than inferring mutations from genotypes alone but the resulting callsets still have high false positive rates.
Results: We identify that the main source of false positive DNMs comes from the use of genotype likelihoods in an otherwise robust mutational model. To address this issue, we propose two alternative methods which build on an existing DNM calling approach DeNovoGear, but with higher accuracy and no decrease in sensitivity.
Furthermore, we developed a method which collects allele specific frequency profiles in the sequenced cohort from across many unrelated samples and identifies sites that either demonstrate high rates of sequencing and mapping errors, or are unlikely to be clinically significant due to their high recurrence rate.

Key words: De novo mutation; DNM; bcftools

Introduction

De novo mutations (DNMs) are new genetic variants found only in the genome of the child and not in the genome of either biological parent. While a typical healthy human has ~60 DNMs of no known health consequence [1], DNMs are also an important source of morbidity among neurodevelopmental disorder (NDD) patients, with at least 31-40% of NDD patients having a DNM directly causing or contributing to their symptoms [2].

To identify DNMs from next generation sequencing data using parent-offspring trios, standard variant calling workflows are used [3]. In the most basic approach, resulting genotypes are then analysed to identify loci where only the child, and neither biological parent, have a heterozygous genotype. However, despite continuing advances in sequencing technology and variant calling protocols, genotyping errors occur at a much higher rate than true DNMs, which makes the detection of Mendelian inheritance violations at the genotype level impractical due to the high false positive rate. Therefore, sophisticated probabilistic models were developed which employed genotype likelihoods, transmission probabilities, and prior probability of observing a DNM [4, 5, 6, 7, 8]. Even though family-aware genotype likelihood-based methods are significantly more accurate than the basic genotype-based method [9], improvements can be made, which we highlight below.

There are three main modes of false-positive DNM calls. First, inherited variants in the child may be falsely inferred as *de novo* because the variant allele is incorrectly called as homozygous reference in both parents. Second, false variants may be called in the child at sites with unexpectedly high sequencing error rates. Third, true parental alternate alleles may go undetected due to insufficient sequencing depth.

The first issue is often linked to conflicting demands on sensitivity imposed on the input genotype information with respect to the presence of the alternate allele in the child and its absence in the parents. In the child, calling must be reliable yet appropriately selective, effectively preventing sporadic alternate reads caused by sequencing and mapping artifacts from being misinterpreted as genuine DNMs. Yet in the parents, calling must instead be oversen-

Compiled on: March 11, 2026.
Draft manuscript prepared by the author.

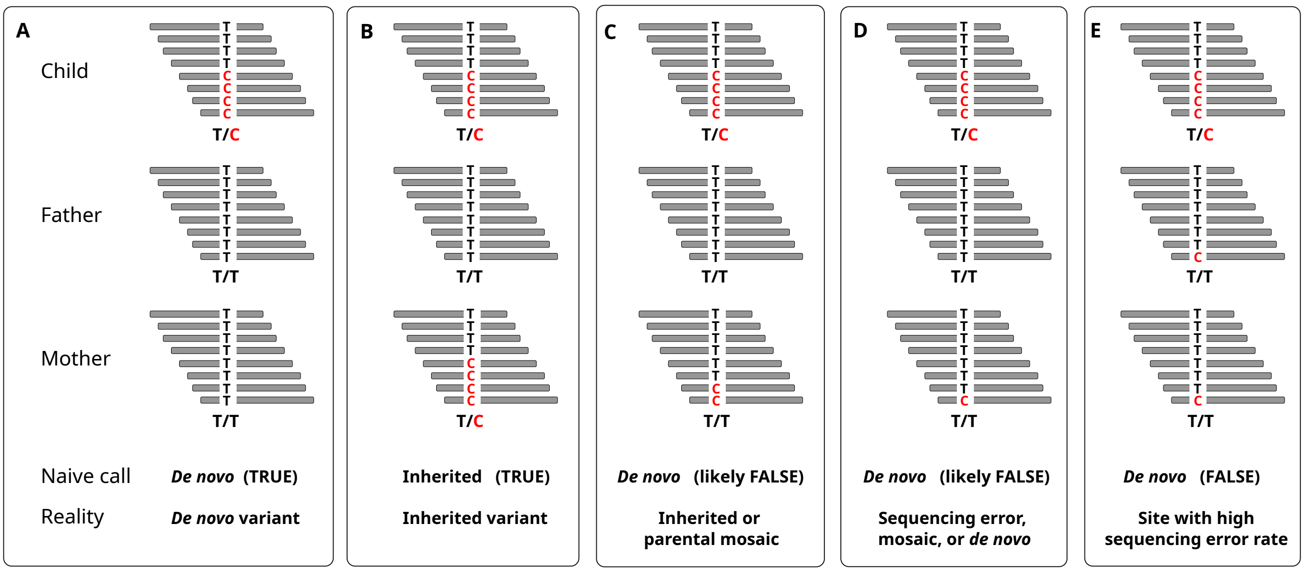

Figure 1. Schematic overview of the core problem of DNM calling. The candidate DNM has the same supporting evidence in the child, but different counts of alternate reads are observed in the parents. The genotypes below each case suggest the most likely genotype as determined by a germline variant caller. Panels **A** and **B** show cases where the ascertainment is trivial. Panels **C**-**E** show the problematic cases where one or both parents have the alternate allele, but the interpretation is uncertain. It can be a misclassified inherited variation **(C)**, sporadic sequencing errors **(D)**, or mapping artefacts **(E)**. Note that although the cases **C** and **D** can also have a valid biological reason, a mosaic post-zygotic parental mutation, the TrioDNM method is not able to distinguish between true parental mosaics and inherited sites where the proportion of the alternate allele in the parent deviates significantly from 50%.

sitive, and highlight the presence of alternate reads in the parents, to prevent an inherited variant from being mistaken for a DNM. These problems are frequently exacerbated by small variations in coverage, number of alternate reads, or mapping and base qualities, and often lead to heterozygous call in the child but homozygous reference calls in both parents (Figure 1). Importantly, the presence of alternate reads in the parents alone is not sufficient evidence for exclusion, because systematic errors (e.g., mapping or alignment artefacts) produce correlated signals across samples, including the child, whereas random sequencing errors are largely independent across individuals. Consequently, excluding all sites with alternate reads in the parents would inflate the false-negative rate for DNMs in the child.

Additional challenges include incorrectly classifying a site as a DNM due to an inherently noisy region with unusually high rate of base miscalls, or failing to detect the parental alternate allele due to insufficient sequencing depth. For example, under a binomial sampling model for reads drawn from a diploid genome, we estimate that approximately seven per thousand heterozygous genotypes will be observed as non-variant sites covered by eight reads. Due to reference mapping bias, this estimate is conservative, particularly for indels, where the bias is more pronounced than for SNVs.

We introduce three methods to improve *de novo* calling and filtering. Methods 1 and 2 extend the genotype likelihood-based model implemented in DeNovoGear [9] by replacing genotype likelihoods with allelic likelihoods, thereby increasing sensitivity to alternate reads in parents. These methods are implemented in the BCFtools/trio-dnm3 plugin and are collectively referred to as TrioDNM. Method 3 analyses the frequency of alternate reads at candidate *de novo* sites in healthy, unrelated parents from the same cohort, enabling detection of false variants at error-prone sites that are difficult to genotype, as well as true variants with high population allele frequencies. This method is implemented in the BCFtools/vrfs plugin and is referred to as VRFS.

Methods

DNG: the DeNovoGear model

The original DeNovoGear model [9], briefly discussed here for reference, identifies DNMs by evaluating joint data likelihoods for all possible combinations of genotypes from mother, father and child (G_M, G_F, G_C) given the observed data (D) as follows

$$L(G_C, G_M, G_F|D) = P(D|G_C, G_M, G_F) \cdot P(G_C|G_M, G_F) \cdot P(G_M, G_F). \quad (1)$$

The first term in Equation 1 is the product of genotype likelihoods in the parent-offspring trio

$$P(D|G_C, G_M, G_F) = P(D_C|G_C) \cdot P(D_M|G_M) \cdot P(D_F|G_F) \qquad (2)$$

and they are provided as input to the program. The second term in Equation 1 represents the transmission probability 0.25, 0.5, or 1 for genotypes compatible with Mendelian inheritance, modulated by a generic germline mutation rate $\mu = 10^{-8}$ for each novel allele. Finally, the third term is the prior probability of obtaining the two parental genotypes G_M and G_F from the population under the neutral coalescent model (Supplement S1).

The Equation 1 is evaluated for all possible combinations of genotypes and the most likely combination that is incompatible with Mendelian inheritance is selected. The probability of a variant being a *de novo* mutation is approximated by the posterior probability of the most likely genotype combination that is incompatible with Mendelian inheritance, as follows:

$$P(\text{DNM}) = \frac{L(G_C, G_M, G_F|D)}{\sum_{c,m,f} L(c, m, f|D)}. \qquad (3)$$

The summation in the denominator is over all possible genotype combinations, including combinations compatible with Mendelian inheritance.

This model has been reimplemented in the BCFtools/trio-dnm3 plugin and is accessible via the `--use-DNG` command line option (Supplements S1 and S2).

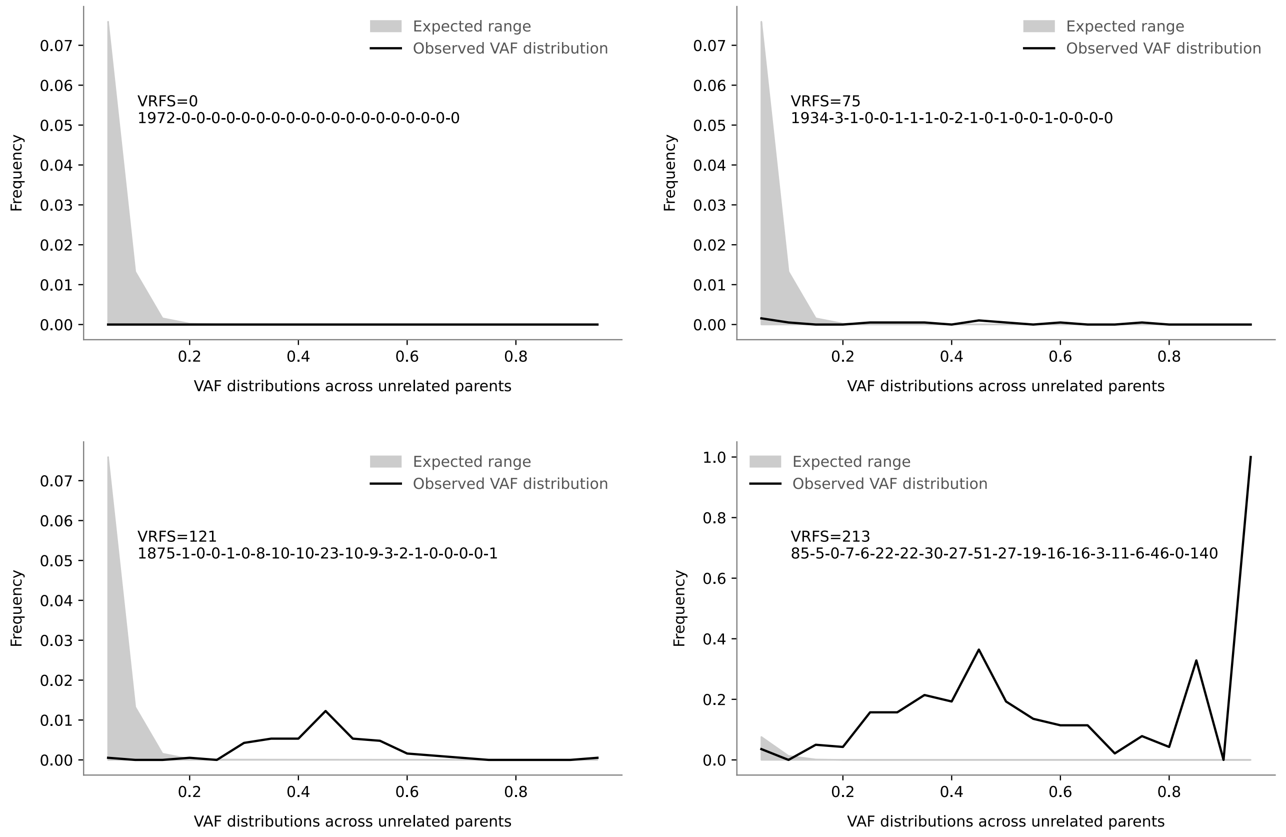

Figure 2. Examples of parental VAF distributions at four sites and the corresponding variant read frequency scores across a range of values, from VRFS=0 (alternate allele not present in the samples) to VRFS=213 (alternate allele is prevalent in the samples, with 140 having alternate homozgyous genotype).

ALM: the allele–likelihood model

The first TrioDNM method, the ALM model, extends DeNovoGear by increasing sensitivity to low-level alternate reads in the parents. This is motivated by the observation that when the proportion of alternate reads deviates substantially from 50%, a heterozygous genotype may appear less likely than a homozygous genotype, even though alternate alleles are clearly present. This occurs because genotype likelihoods are, by design, robust to independent sequencing errors for germline calling, but are less suitable for *de novo* mutation detection, which requires sensitivity to systematic artifacts and low-level mosaicism.

Thus in order to increase the sensitivity to alternate reads, we replace the parental genotype likelihoods in Equation 2 with parental allelic likelihoods:

$$P_{\mathrm{ALM}}(D|G_C, G_M, G_F) = P(D_C|G_C) \cdot A(D_M|G_M) \cdot A(D_F|G_F). \quad (4)$$

For simplicity of notation, we limit the description to single nucleotide changes, but insertions and deletions are handled identically. We introduce a new variable Q_x which will represent the probability that the allele $x \in \{A, C, G, T\}$ observed in a given sample reflects a true signal, rather than a sequencing or mapping artefact. Let $\epsilon_{x,i}$ denote the error probability for the i-th read carrying allele x, defined as the maximum of the corresponding base-calling and mapping error probabilities. Then if the base x is present in reads covering a genomic position k_x times, the probability Q_x can be expressed as

$$Q_x = \begin{cases} 1 - \prod_{i=0}^{k_x} \epsilon_{x,i} & \text{if } k_x > 0, \\ 0 & \text{otherwise.} \end{cases} \quad (5)$$

For a true *de novo* allele x, parental probabilities Q_x are expected to be small; otherwise, the allele likely reflects inheritance or systematic mapping bias. The values Q_x are computed during the the BCFtools/mpileup step when the option `--annotate QS` is enabled (Supplement S2). In practice, the calculation is performed in log space to prevent numerical underflow, although this implementation detail is not reflected in the simplified formulation presented here for clarity.

The parental allelic likelihoods $A(D|G)$ in Equation 4 are then calculated separately for the mother and the father in the context of the parental genotype G with alleles a and b as follows:

$$A(D|G = ab) = \prod_x \begin{cases} Q_x & \text{if } x \in \{a, b\}, \\ 1 - Q_x & \text{otherwise.} \end{cases} \quad (6)$$

where the multiplication is over all bases x observed at the position in the trio. The first case contributes with a large value when the alleles of the tested parental genotype ab have a strong presence in the parent, the second case penalises the presence of unexpected alleles. In this sense, the model shifts the focus from genotype inference to evidence evaluation, asking whether allele x is supported by the data rather than how it was generated.

Although ALM is formulated within a principled statistical framework, it combines child genotype likelihoods with parental allele-level likelihoods, resulting in a hybrid model that departs from a fully joint generative formulation of the trio. This model has practical limitations. In particular, the parental emission model for the candidate *de novo* allele is overly stringent in the presence of random sequencing errors. To mitigate this, we introduce a tolerance parameter allowing a user-controlled fraction of unexpected reads in the parental data. The default tolerance (4.5% when the signal is present in one parent; 1.1% when present in both) were selected

empirically from inspection of random set of *de novo* candidates and typical background artefact levels in parental samples. We apply a more stringent threshold when both parents show evidence of the alternate allele, because independent sequencing errors are unlikely to recur at the same site in two separate samples; thus, concordant parental signal more plausibly reflects a systematic site-specific noise than a true *de novo* event.

Another limitation of this approach is that uncertainty arising from low parental sequencing depth is not propagated into the final probability. As a result, sites with limited parental coverage may receive excessively confident *de novo* score despite weak supporting evidence.

Motivated by these considerations, we investigated an alternative model in which parental evidence is modeled directly using a Dirichlet–multinomial distribution over allele counts, replacing the parental allelic likelihood terms $A(D|G)$.

DMM: the Dirichlet–multinomial model

The second TrioDNM method, DMM, was motivated by the observation that although ALM markedly improves upon DNG, it exhibits overconfidence at low parental depth and reduced discriminatory resolution among high-scoring variants, limiting the ability to stratify calls at the upper end of the score distribution. Sensitivity to alternate reads observed in the parents can also be modelled using count-based statistical models. In this approach, we model parental evidence using a three-component Dirichlet–multinomial distribution.

Specifically, for a given parental genotype hypothesis $G = ab$, we summarise the sequencing reads as a count vector (k_a, k_b, k_x), where k_a and k_b are the numbers of reads supporting alleles a and b, and k_x counts all other bases. The corresponding probability vector (p_a, p_b, p_x) is computed from mean per-read base-error probabilities, or from user-specified maximum quality threshold, and subsequently normalized to sum to one. Parental allele counts are then modeled using a Dirichlet–multinomial distribution with concentration parameter ϕ, allowing additional variability in allele counts beyond that expected under multinomial sampling (i.e., overdispersion). The parental allelic likelihood is therefore replaced by

$$A'(D|G = ab) = DM(k_a, k_b, k_x \mid p_a, p_b, p_x; \phi). \qquad (7)$$

Like the original DNG model, this approach assumes a complete generative error model in which base and mapping quality scores fully capture sequencing and alignment uncertainty. In practice, this assumption is only partially satisfied: while mapping qualities reflect read uniqueness and alignment ambiguity, they do not account for sample contamination or mismatches arising from structural variation. Moreover, the framework evaluates only the maximum-likelihood combination of *diploid* genotypes and therefore cannot flag cases in which *none* of the genotype configurations adequately explains the observed data. To address this, we extend Equation 3 by introducing additional terms to define an augmented probability score as follows:

$$S_{\mathrm{DMM}} = \frac{P_{\mathrm{DMM}}(G_C, G_M, G_F|D)}{\sum_{c,m,f} P_{\mathrm{DMM}}(c, m, f|D)} \cdot P_{\mathrm{noise}} \cdot P_{\mathrm{parent}} \cdot P_{\mathrm{mosaic}}. \qquad (8)$$

Here, the noise term P_{noise} and the parental-emission term P_{parent} are defined as upper binomial tail probabilities for observing at least k unexpected observations among n reads under the selected genotype,

$$P(K \geq k), \quad K \sim \mathrm{Binomial}(n, \epsilon),$$

where ϵ denotes the mean per-read error rate. In the case of P_{noise}, unexpected reads support alleles incompatible with the selected genotype, whereas for P_{parent} they correspond to parental reads supporting the candidate *de novo* allele. To account for random sequencing and mapping errors, we introduce a user-controlled tolerance on parental alternate read counts. Up to a specified absolute or fractional number of alternate reads is treated as background noise and excluded from the test; the binomial tail probability is then evaluated on the remaining excess reads only. This treatment and the default threshold values are identical to those used in the ALM method.

The mosaicity term P_{mosaic} is defined as the posterior probability that the underlying variant allele fraction (VAF) exceeds a user-controlled mosaicity threshold under a Beta–Binomial model, given the observed allele counts

$$P_{\mathrm{mosaic}} = P(m \geq m_0 \mid D), \quad m \mid D \sim \mathrm{Beta}(a, b).$$

The effective read depth is capped at 50× (i.e., depths exceeding 50 are downscaled) to prevent overconfidence at high coverage.

VRFS: the variant read frequency score

Studies of DNMs in large cohorts reveal that some candidate mutations, which initially appear genuine, are also found frequently in unrelated, healthy parents. Such recurrence is unlikely under a true *de novo* model and instead points to systematic technical effects, including dataset-specific artefacts, misalignment, common polymorphisms, or genomic complexity (e.g., paralogous regions). Identifying and downweighting these sites is therefore essential for controlling false discovery rates.

To detect recurrent loci, we developed a method that compares each candidate site against an expected noise profile. This expected profile is generated by collecting the proportions of alternate reads across a reference set of individuals (parental samples in our study) and across a set of high-confidence calls, as described below and in Supplementary Figure S3. True DNMs are expected to have very few individuals in the reference set that have a high proportion of variant reads.

For a tested site, we collect the variant allele fraction (VAF) observed across a reference set of individuals and summarize this distribution using a histogram with k bins (by default $k = 20$; see Supplementary Figure S4). Let $(f_1, \ldots, f_k)$ denote the empirical VAF distribution, where $f_i = n_i/n$, with n_i denoting the number of samples whose VAF falls in bin i, and n the total number of samples.

Using a set of m high-confidence reference sites $\{s_1, \ldots, s_m\}$ in which only a small number of samples exhibit high VAF values (Supplementary Figures S3C and S5), we estimate a variance parameter σ_i^2 for each histogram bin i. Specifically, if $f_{i,s}$ denotes the frequency in bin i at site s, then

$$\sigma_i^2 = \frac{1}{m} \sum_{s=1}^{m} \left(f_{i,s} - \bar{f_i}\right)^2,$$

where

$$\bar{f_i} = \frac{1}{m} \sum_{s=1}^{m} f_{i,s}.$$

We then define the variant read frequency score (VRFS) to quantify how strongly the empirical VAF profile at a site deviates from the reference profiles observed at high-confidence sites. For simplicity, we assume normally distributed noise and $\bar{f_i} = 0$ for $i > 1$ at *de novo* sites. Under this assumption, the log-likelihood contribution of

each bin is proportional to f_i^2/σ_i^2, and we define the VRFS score as

$$\mathrm{VRFS}(f_1, \ldots, f_k) = 10 \log \left(1 + \sum_{i=2}^{k} \frac{f_i^2}{\sigma_i^2}\right) \tag{9}$$

This formulation can be interpreted as a variance-normalized squared deviation of the observed VAF distribution from the reference profile, analogous to a χ^2-type statistic, followed by logarithmic scaling. To ensure numerical stability, each σ_i is bounded by a small constant, preventing division by zero. The first bin (VAF = 0) is excluded because invariant sites dominate this bin and provide no information about recurrent sequencing noise, given that frequencies are normalized. Applying a logarithmic transformation compresses extreme values, stabilizing the statistic's range and preventing outliers from dominating, while adding a constant ensures that sites matching the reference profile exactly receive a score of zero (Figure 2 and Supplementary Figure S6).

Putative DNMs with multiple alternate alleles pose a problem – while multiple alternate alleles observed frequently in unrelated samples is often a hallmark of mapping artefacts in difficult regions, there are also rare cases of genuine DNMs at polymorphic sites. We address this issue differently for SNVs and indels. For multiallelic SNVs, we construct a VAF profile and compute the VRFS value for each alternate allele x separately. The scores are then shifted toward the noisiest allele m according to

$$\mathrm{VRFS}'_x = \mathrm{VRFS}_x + 0.75(\mathrm{VRFS}_m - \mathrm{VRFS}_x). \tag{10}$$

The coefficient 0.75 was chosen heuristically to ensure that the score of a multiallelic site is largely driven by the noisiest allele while still retaining some contribution from the allele-specific signal. Because insertions and deletions are more difficult to genotype, prone to alignment errors, and often ambiguously aligned, we treat all indels as biallelic regardless of the number of alternate sequences.

A user-friendly, performative version of this model has been implemented in the BCFtools/vrfs plugin (Supplement S2).

Data description

To evaluate the performance of the methods, we used both simulated and exome sequencing data.

Simulated data for evaluation of filtering performance

The initial set comprised 5,000 simulated *de novo* variants; 11 overlapped variants present in parental genomes and were therefore excluded. Reads were then simulated from an artificially constructed trio with the remaining 4,989 variants using Mason [10], and simulated reads were aligned to GRCh38 using BWA-MEM [11]. Subsequent variant calling produced an initial candidate callset with 4,987 variants classified as true positives, 1,039 as false positives, and 2 as false negatives (Supplement S7).

Raw exome callset for evaluation of filtering performance

The wider exome sequencing data generated for 1,094 trios (1,094 children and 1,981 parents) from the Born in Bradford study (BiB). See [12] for details on sample collection and exome sequencing. The Illumina NovaSeq 100bp paired-end reads were aligned to GRCh38 using BWA-MEM. Initial variant calls were made with GATK HaplotypeCaller v4.3.0.0 following GATK best practices [3] and a raw candidate *de novo* callset was generated by selecting sites that do not conform to Mendelian inheritance using the naive function of the BCFtools/trio-dnm3 plugin (Supplement S2).

The initial callset comprised 279,410 candidate variants. To reduce its size prior to downstream analyses, we applied a lenient prefiltering procedure. To avoid inherited variants misclassified as *de novo* due to stochastic binomial sampling of reads from the diploid genome during sequencing, we required a minimum sequencing depth of 10× in all three samples, accounting for difference in inheritance patterns on the sex chromosomes. We further excluded calls located in dense variant clusters, with four or more variants within a 60 bp window. Finally, candidates were ranked separately by each quality score (DNG, ALM, and DMM) using the commands shown in the Supplement S2, and the callset was truncated to guarantee at least five times the expected number of *de novo* calls under any ranking. The expected number was calculated using a mutation rate of $4 \cdot 10^{-8}$ estimated from 2,132 Icelandic families (Supplementary Materials of [13]). The prefiltered candidate *de novo* callset consisted of 11,904 SNVs, 1,781 deletions, and 867 insertions.

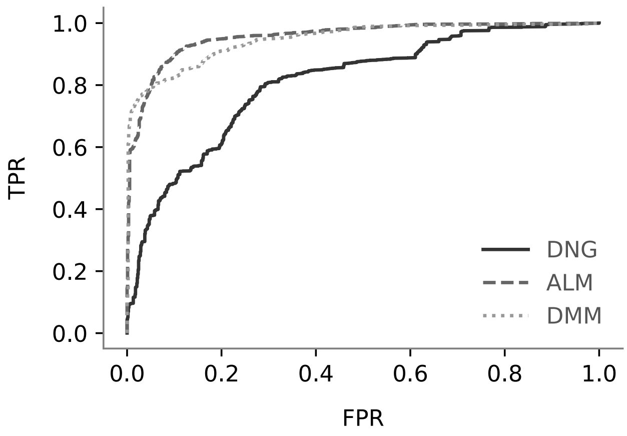

Figure 3. Peformance of DNG, ALM, and DMM evaluated on exome sequencing data. Receiver operating characteristic (ROC) curves showing true positive rate (TPR) versus false positive rate (FPR) across varying score thresholds. Areas under the curve (AUC) were 0.808 for DNG, 0.957 for ALM, and 0.947 for DMM. See also Supplementary Figure S8.

Next, to evaluate methods performance, we established a labelled truth set by manual inspection in IGV [14]. Variants were selected by taking the top-ranked calls from each method, ensuring that the highest-scoring candidates under every method were included in the evaluation set. In total, we reviewed 5,958 variants and classified 3,037 (51%) as true positive, 1,562 (26%) as false positive, and 1,359 (23%) as uncertain.

Clean callset for VRFS evaluation

To evaluate the VRFS method, we refined the candidate callset by applying stringent thresholds to all three quality metrics (DNG, ALM, and DMM). Threshold values were determined empirically using the manually curated sites by inspecting the distribution of true positives (Supplementary Figure S8) and selecting cutoffs that retained 95% of validated true calls. Variants exceeding all three thresholds were retained, subsequently ranked by the selected score, and truncated to a fixed number of top-scoring candidates corresponding to twice the number of expected *de novo* mutations for downstream evaluation.

The callset filtered in this manner contained a significant proportion of duplicate sites, with 19% of SNVs and 5% of indel sites appearing multiple times. These duplicates are likely artifacts rather than genuine DNMs; indeed, 95% of these calls were repeatedly observed among unrelated parents. To highlight the utility of the VRFS method after creating a clean callset using conventional approaches, we removed these duplicates as evident artifacts. This refinement resulted in a filtered callset comprising 2,940 SNVs and 287 indels, which we then used to further investigate the properties of VRFS.

It should be noted that filtering pipelines employed in real-world applications are typically more complex, incorporating additional criteria to optimize both specificity and sensitivity (Supple-

ment S21).

Results

We evaluated DNM callsets generated by applying the proposed methods to both simulated data and exome sequencing data from 1,094 parent–offspring trios. We then investigated the properties and behaviour of the VRFS metric.

Modeling parental allele emission improves filtering performance

We evaluated method performance using three complementary metrics: Ti/Tv, VAF25, and classification performance summarized by the area under the receiver operating characteristic curve (AUC). Ti/Tv, the ratio of nucleotide transitions to transversions, serves as a proxy for biological plausibility, with higher values indicating a cleaner callset (Supplementary Figure S9). VAF25, defined as the proportion of calls with fewer than 25% of reads supporting the alternate allele in the child, captures enrichment of low-allelic-fraction events; lower values indicate a cleaner callset with fewer artefacts (Supplementary Figure S10). ROC curves plot true positive rate against false positive rate and directly quantify trade-offs between sensitivity and specificity (Figure 3).

All three metrics consistently indicate that the TrioDNM methods, ALM and DMM, achieve lower false discovery rates than DNG at comparable levels of sensitivity, suggesting that explicitly modeling parental emission of the *de novo* allele substantially improves filtering performance. Results on simulated trio data as well as manual review of outliers further support this conclusion (Supplementary Figures S7 and S11).

The impact of sequencing depth on performance

One concern when extending the DNG framework was a potential sensitivity to sequencing depth. Although the median minimum depth across trio members in the target regions was 38×, sequencing coverage varies substantially across loci. We therefore assessed how sequencing depth influences method performance by evaluating ROC curves at minimum depth thresholds of 10×, 20×, and 30× (Supplementary Figure S12).

Across all depth cutoffs, the relative ranking of methods remained consistent, with ALM and DMM outperforming DNG. For DNG, the AUC ranged from 0.808 to 0.837, whereas ALM achieved AUCs between 0.957 and 0.981. DMM performed comparably to ALM overall, although at the lowest depth (10×) its performance was slightly reduced relative to ALM (AUC 0.947 vs. 0.957). At 20× and 30×, however, DMM reached the highest accuracy (AUC 0.987) and showed earlier saturation of the ROC curve.

These results indicate that, as expected, sequencing depth influences performance. At low depth (10×), DMM is more conservative method than ALM, likely reflecting its explicit accommodation of potentially missed alternate reads in parents due to binomial sampling. However, DMM reaches ROC saturation earlier than the other methods, indicating more efficient discrimination once sufficient depth is available.

Confluence of TrioDNM and VRFS scores

Considering the size of the sequenced mutational target (~36.7 Mb across 1,094 individuals) and the expected *de novo* mutation rate (~10^{-8} per base per generation), true *de novo* mutations are unlikely to occur at common variant sites or to arise independently in multiple individuals. Genuine recurrent *de novo* events are therefore expected to be orders of magnitude rarer than false positive calls.

The Variant Read Frequency Score (VRFS; Methods) quantifies recurrence of the alternate allele across unrelated samples, enabling identification of loci that most likely reflect sequencing artefacts or, if truly recurrent, are unlikely to be clinically relevant. It is reasonable to expect that calls identified as low quality by TrioDNM methods will often be also identified as low quality by VRFS.

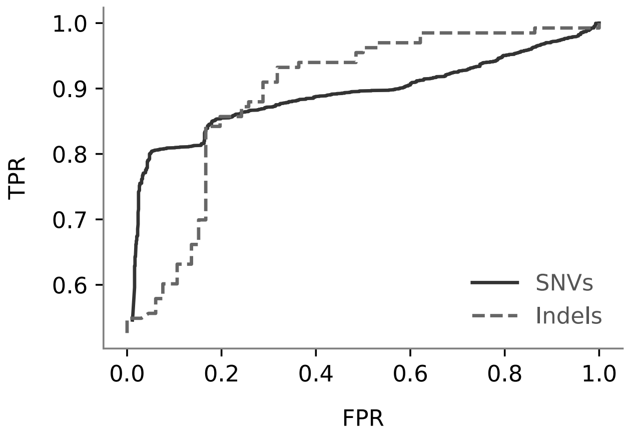

Figure 4. Performance of VRFS. ROC curve for VRFS, true positive rate (TPR) is plotted against false positive rate (FPR) across varying VRFS thresholds. Areas under the curve (AUC) were 0.884 for SNVs and 0.894 for indels.

The rankings by DMM and VRFS showed a moderate negative monotonic association in both the broader exome callset (11,904 SNVs) and the refined callset (2,940 SNVs) (Kendall's τ_b = -0.37 and -0.42, respectively). ROC analysis further revealed that VRFS can also distinguish true from false positive calls, although less effectively than the TrioDNM scores, as expected (Figure 4).

The proportion of recurrent and highly recurrent sites (VRFS≥100 and VRFS≥150, respectively), which can be viewed as a proxy for artefactual burden, increases with callset size for both SNVs and indels. However, the magnitude of this effect differs markedly between variant classes. At the expected size of the true *de novo* callset, only 2.9% of SNVs are recurrent (VRFS≥100), whereas approximately 40% of indel sites are recurrent (Supplementary Figure S13). This confirms that indel calls are disproportionately affected by systematic recurrence, consistent with technical challenges associated with repetitive sequence contexts and alignment ambiguity.

Together, these results indicate that sites with large VRFS values and low TrioDNM scores are enriched for artefacts. Both capture different aspects of sequencing noise: VRFS reflects the recurrence of variant reads across unrelated samples, whereas TrioDNM evaluates the likelihood of the variant under a trio inheritance model. Because these metrics rely on distinct signals and data patterns, they provide complementary information. Consequently, filtering candidate *de novo* mutations using both metrics is expected to improve overall callset quality.

Sources of recurrent candidate DNMs

We next examined the genomic correlates of recurrent DNMs. Apparent recurrence may arise from technical artefacts, such as misclassified inherited variants, or from genuine biological processes, including polymerase slippage, gene conversion, and clonal expansion of mutant blood cells. We evaluate both possibilities here.

We anticipate that many recurrent *de novo* sites are within repetitive regions of the genome, leading to reduced alignment quality in short-read sequencing. Indeed, 59% of the highly recurrent SNVs (VRFS≥150) and 57% of the highly recurrent indels in the clean callset are found in paralogs, segmental duplications and repetitive regions (Supplementary Figure S14). The genomic context of recurrent calls differed markedly between SNVs and indels. Among non-recurrent sites (VRFS<50) SNVs were predominantly located in unique sequence, whereas indels already showed substantial enrichment in repetitive regions. With increasing VRFS, the proportion of calls in repetitive regions increased for both variant types, but the pattern of enrichment diverged. Recurrent and highly re-

current SNVs (VRFS≥50 and VRFS≥150) were preferentially located in segmental duplications and paralogous regions, consistent with mapping ambiguity in duplicated sequence. In contrast, recurrent indels were most strongly enriched in simple repeats, with comparatively smaller contributions from segmental duplications. These results suggest distinct mechanisms underlying recurrence: duplicated genomic architecture appears to drive recurrent SNV calls, whereas local sequence repetitiveness plays a dominant role for indels, likely reflecting a combination of polymerase slippage and alignment ambiguity in short-read sequencing.

We also investigated whether stochastic sampling bias in sequencing contributes to the misclassification of inherited variations as DNMs. We found that 0.3% of SNVs and 12% of indels in the clean callset have a significantly higher probability of being misclassified as *de novo* due to failure to sample the alternate allele in the parents at low sequencing depth ($p \geq 10^{-4}$, binomial probability). The prevalence of such sites strongly correlates with the VRFS value. Notably, this effect is dependent on the scoring method used, with ALM substantially over-scoring sites with low parental depth (Supplementary Figure S15).

Regardless of the mechanism, it is reasonable to expect that both true common variants miscalled as DNM and false variants miscalled due to high error rate sites, are also likely to be present in gnomAD [15]. Indeed, in the clean callset, 45% of sites with a *de novo* SNV allele and 68% of sites with a *de novo* indel allele were found also in gnomAD as a polymorphic site, of which 9% and 29%, respectively, had allele frequency (AF) greater than 0.1 (Supplementary Figure S16). The majority of these sites, with AF>0.1, were also highly recurrent in unrelated samples in our study, i.e. 82% of these SNVs and 91% of indels had VRFS≥150.

While we observed that large VRFS values predominantly highlight misclassified inherited variation (such as in Supplementary Figure S17) and false positives in difficult-to-align regions (such as in Supplementary Figure S18), it is possible that genuine highly recurrent somatic mutations could also be present among the candidate DNMs with high VRFS scores. For completeness, we provide the list of recurrent sites identified in this study, although it was derived from a single cohort and its generalizability to other datasets has not been evaluated (Supplement S19).

The stability of the VRFS method

A set of reference samples is required to generate VAF profile distributions. The sensitivity of the method to common variation and artefacts increases with the number of samples. Reducing the number of samples by 75% (from 1,981 to 500) reduces the sensitivity by 0.2% (i.e. 0.2% of recurrent sites with VRFS≥100 are newly reported as non-recurrent with VRFS<50) and limiting the number of samples by 95% (from 1,981 to 100) reduces the sensitivity by 1.7% (Supplementary Figure S20). This suggests the method can be used with relatively small cohorts – parental samples of as few as 50 trios can capture 98.7% of problematic sites.

Note that the method is also stable with respect to the bin size (by default $k = 20$) and the values remain highly correlated over a range of bins (Supplementary Figure S4).

Discussion

We developed three related methods to assist in the calling and filtering of *de novo* SNVs and short indels. Two of these approaches are complementary in their modeling assumptions, and one is implemented in two alternative formulations. All methods were implemented as standard BCFtools plugins with usability and computational efficiency in mind.

The first tool, TrioDNM, is designed for use in parent–offspring trios and addresses key limitations of existing approaches. Prior methods rely primarily on parental genotype likelihoods to evaluate the presence of the putative *de novo* allele. In contrast, we implemented two alternative strategies that leverage allelic likelihoods evaluated conditional on the child's genotype. We demonstrate that both ALM and DMM scores substantially improve the ranking of *de novo* candidates and yield a more refined callset compared to DNG, the previous formulation of the model. Overall, DMM provides the most accurate discrimination and, relative to ALM, appropriately accounts for uncertainty arising from low parental sequencing depth.

The second method, VRFS, was developed for DNM calling but can have broader utility for other applications, e.g. generating high quality rare SNV/indel callsets in duos (a child plus one biological parent) and other non-trio data. The method collects allelic frequency profiles from many samples, enabling the filtering of sites with uncertain clinical relevance, such as misclassified inherited variants or sites that are difficult to genotype. Using the VRFS method, we found that 3% of apparent *de novo* SNVs and 40% of indels are also present as variants in unrelated samples. This observed recurrence is likely attributable to several factors, with distinct patterns for SNVs and short indels. Recurrent candidate *de novo* SNVs were more prevalent in segmental duplications and paralogous regions, whereas recurrent indels occurred predominantly in simple repeats and SINE elements. These contrasting patterns likely reflect different underlying mechanisms. In duplicated and paralogous regions, recurrent SNV calls may arise from mapping ambiguity. In contrast, the enrichment of indels in simple repeats is consistent with polymerase slippage, which may occur both biologically during DNA replication and technically during PCR amplification and sequencing. Furthermore, the degree of recurrence strongly correlates with stochastic sampling bias, where lower sequencing depth in the parents increases the likelihood of missing the alternate allele.

The recommended approach for utilizing these methods is to rank candidate calls by the DMM score, identifying variants that appear to be genuine DNMs within the parent-offspring trio. Subsequently, pooled information from multiple samples can be used to filter based on the VRFS value, effectively eliminating common artifacts and inherited variation. Note that a real-world filtering pipeline is typically more complex and incorporates additional filtering criteria (see an example in Supplement S21). The two methods presented here are specifically designed to address only a subset of the failure modes observed in sequencing data.

While public genomic resources like gnomAD could serve the same purpose for filtering out common variants, they cannot account for cohort-specific sequencing artifacts, or may not be available for the studied organism or reference build. Our findings show that relatively small sample sizes are sufficient to capture the majority variation—for instance, VRFS applied on 100 samples could identify 98.7% of recurrent sites. Also, while the calculation of VRFS values is sensitive to VAF profile variances $\{\sigma_i^2\}$, which are precomputed from a set of high-confidence sites, we show that these error profiles can be reused between studies (Supplementary Figure S5).

The accuracy of the TrioDNM method is contingent upon the quality of its input data, namely genotype and allelic likelihoods. These inputs are frequently subject to inaccuracies, particularly in the case of indels, which are notoriously challenging to genotype accurately. However, the model could be improved—in theory both DeNovoGear and TrioDNM models could include allele specific mutation rates to account for known effects such as 5-Methylcytosine at a CpG being more prone to transition than unmethylated cytosine due to spontaneous deamination. Furthermore, while the VRFS method takes into account multiallelic SNVs individually, its treatment of indels is limited to a site-level assessment.

In general, the methods are not suitable for all forms of genetic variation, such as copy number variation (CNVs). Even though the genotype and allelic likelihoods, which serve as input data to the model, could be in principle provided for any variant type, in practice their accuracy is not sufficient to produce reliable results. Furthermore, while TrioDNM can detect DNMs at common poly-

morphic sites in the population which modify the alternate allele to reference (i.e. 0/1 to 0/0), the VRFS method will recognize such sites as common polymorphism and a site that falls in this category will receive a high VRFS score.

Availability of Supporting Source Code

The software is available free of charge under the MIT license and can be accessed at https://github.com/samtools/bcftools and https://github.com/HurlesGroupSanger/trio-dnm-calling. See also Supplements S2 and S21.

Acknowledgements

We thank the reviewers for their comments and suggestions that helped improve the manuscript and motivated the development of additional methodological extensions presented in this work.

This work was supported by Wellcome Grant reference number 220540/Z/20/A, Wellcome Sanger Institute Quinquennial Review 2021-2026. For the purpose of Open Access, the author has applied a CC BY public copyright license to any Author Accepted Manuscript version arising from this submission

This study makes use of data from the Born in Bradford programme, which is only possible because of the enthusiasm and commitment of the children and parents in BiB. We are grateful to all the participants, health professionals, schools and researchers who have made Born in Bradford happen.

EJG is an employee of and holds shares in Insmed, Inc.

References

1. Kaplanis J, *et al*. Genetic and chemotherapeutic influences on germline hypermutation. Nature 2022;605:503–508.
2. Wright C, *et al*. Genomic Diagnosis of Rare Pediatric Disease in the United Kingdom and Ireland. N Engl J Med 2023;388(17):1559–1571.
3. van der Auwera G, O'Connor BD. Genomics in the Cloud: Using Docker, GATK, and WDL in Terra. O'Reilly Media, Incorporated; 2020.
4. Ramu A, *et al*. DeNovoGear: de novo indel and point mutation discovery and phasing. Nat Methods 2013;10(10):985–7.
5. Wei Q, Zhan X, Zhong X, Liu Y, Han Y, Chen W, et al. A Bayesian framework for de novo mutation calling in parents-offspring trios. Bioinformatics 2014 12;31(9):1375–1381.
6. Francioli LC, *et al*. A framework for the detection of *de novo* mutations in family-based sequencing data. Eur J Hum Genet 2017;25:227–233.
7. Kolesnikov A, Goel S, Nattestad M, Yun T, Baid G, Yang H, et al. DeepTrio: Variant Calling in Families Using Deep Learning. bioRxiv 2021;.
8. Khazeeva G, Sablauskas K, van der Sanden B, Steyaert W, Kwint M, Rots D, et al. DeNovoCNN: a deep learning approach to de novo variant calling in next generation sequencing data. Nucleic Acids Research 2022 06;50(17):e97–e97.
9. Conrad DF, *et al*. Variation in genome-wide mutation rates within and between human families. Nat Genet 2011;43(7):712–714.
10. Holtgrewe M. Mason – A Read Simulator for Second Generation Sequencing Data. Technical Report FU Berlin 2010 October;.
11. Li H. Aligning sequence reads, clone sequences and assembly contigs with BWA-MEM. ArXiv 2013;26 May.
12. Koko M, Fabian L, *et al*. Exome sequencing of UK birth cohorts. Wellcome Open Res 2024;9:390.
13. Gunnar Palsson HJ*et al* Marteinn T Hardarson. Complete human recombination maps. Nature 2025;639:700–707.
14. Thorvaldsdóttir H, *et al*. Integrative Genomics Viewer (IGV): high-performance genomics data visualization and exploration. Briefings in bioinformatics 2013;14,2:178–192.
15. Chen S, Francioli LC, Goodrich JK, *et al*. A genomic mutational constraint map using variation in 76,156 human genomes. Nature 2024;625:92–100.

Click here to access/download
Supplementary Material
supplement.pdf
