# Supplementary material for: Improved discovery of de novo mutations using TrioDNM and VRFS
Source: Gigascience. 2026 Jun 9;15:giag068. doi: 10.1093/gigascience/giag068 (PMC13289757; doi:10.1093/gigascience/giag068)
Supplement: giag068_GIGA-D-25-00258_revision_2 [file giag068_giga-d-25-00258_revision_2.pdf]

GigaScience

Improved discovery of de novo mutations using TrioDNM and VRFS

--Manuscript Draft--

**Manuscript Number:**	GIGA-D-25-00258R2
**Full Title:**	Improved discovery of de novo mutations using TrioDNM and VRFS
**Article Type:**	Research
**Funding Information:**	Wellcome Trust (220540/Z/20/A) — Prof Matthew E Hurles<br>Wellcome Sanger Institute (Quinquennial Review 2021-2026) — Prof Matthew E Hurles
**Abstract:**	Background: Identifying de novo mutations (DNM) is an important component of both genetic research studies and clinical diagnostic workflows, but is complicated by distinguishing true mutations from sequencing errors. Likelihood-based error models are more accurate than inferring mutations from genotypes alone but the resulting callsets still have high false positive rates.<br>Results: We identify that the main source of false positive DNMs comes from the use of genotype likelihoods in an otherwise robust mutational model. To address this issue, we propose two alternative methods which build on an existing DNM calling approach DeNovoGear, but with higher accuracy and no decrease in sensitivity. Furthermore, we developed a method which collects allele specific frequency profiles in the sequenced cohort from across many unrelated samples and identifies sites that either demonstrate high rates of sequencing and mapping errors, or are unlikely to be clinically significant due to their high recurrence rate.
**Corresponding Author:**	Petr Danecek<br>Wellcome Sanger Institute<br>Hinxton, UNITED KINGDOM
**Corresponding Author Secondary Information:**	
**Corresponding Author's Institution:**	Wellcome Sanger Institute
**Corresponding Author's Secondary Institution:**	
**First Author:**	Petr Danecek
**First Author Secondary Information:**	
**Order of Authors:**	Petr Danecek
	Eugene J Gardner
	Joanna Kaplanis
	Matthew E Hurles
	Sarah J Lindsay
**Order of Authors Secondary Information:**	
**Response to Reviewers:**	Response to the editor:<br><br>Thank you for your comments and suggestions.<br><br>For the SciCrunch RRID identifier, our past publication with GigaScience used RRID:SCR_002105.<br><br>We checked the suggested reference and it is not directly relevant to our submission.

Powered by Editorial Manager® and ProduXion Manager® from Aries Systems Corporation

Response to the reviewers:

Thank you for your comments and suggestions. We have further revised the manuscript and expanded sections where additional clarification was needed.

Reviewer reports:
Reviewer #1: The authors have addressed my previous concerns, and the manuscript has been significantly improved. I have no further comments and recommend the paper for publication.

Thank you

Reviewer #2: I would like to thank the authors for revising their manuscript according to the
our comments. This version is significantly improved. It is more clear and includes more details about the methods. They've also added some new methods which has given me new things to think about. I also appreciate the results now include more manually curated sites and simulated data as well.

Qx statistic and allelic likelihoods.

I think that I better understand the Qx statistic in this version. Roughly, it is a statistic whose value is governed by estimates of sequencing and mapping error when coverage is low but approaches 1 when coverage is high. In turn, allelic likelihoods are near 1 if there is a lot of coverage and no reads disagree with the genotype, and near 0 if some reads disagree with the genotype. If my understanding is correct, I recommend adding a few more sentences in the ALM description to make this intuition more clear to the reader.

Your understanding is correct and we added a few sentences to explain this better:

The product term corresponds to the probability that all reads car- rying allele x can be explained as errors. Thus, Qx is the comple- mentary probability that the observed support for allele x is not entirely attributable to error, and approaches one when x is sup- ported by high-quality reads. For a true de novo allele x, parental Qx values should be low; elevated values indicate parental support for the allele, consistent with inheritance or systematic mapping bias.
...
This gives high likelihood when the parental reads strongly support alleles compatible with genotype ab. Conversely, a high-confidence allele inconsistent with ab yields a large Q_x, making $1-Q_x$ small and penalizing the likelihood.

Unlike genotype likelihoods which measure P(the set of base calls | genotype), allelic likelihoods seem to measure P(the alleles in the genotype are supported and no other alleles are supported | genotype). If the DNG model approximates the P(de novo mutation | data), would the ALM model approximate P(de novo mutation & parent data contains no systematic artifacts | data)?

Yes, that is the correct interpretation of the model

The Dirichlet-multinomial model.

Powered by Editorial Manager® and ProduXion Manager® from Aries Systems Corporation

I appreciate the authors using a Dirichlet-multinomial model for representing genotype likelihoods as I have found it superior to standard multinomial models for calculating genotype likelihoods from allele depths. However, the authors appear to only use it for parental genotype-likelihoods, but I see no reason why this it should not also be used for child genotype-likelihoods. I would appreciate additional justification for this choice.

The reason is that the mpileup likelihoods are more robust to systematic errors. However, DMM can still be used for the child's genotype. We have extended the text to clarify this point:

By default, this replacement is limited to the parental likelihoods. For the child, the original BCFtools/mpileup model is used as before, because it accounts for correlated read errors. In contrast, the Dirichlet--multinomial model summarizes the data as allele counts and does not explicitly model read-level error dependencies. The latter can also be applied to the child likelihood using the \texttt{--with-cAD} option.

The authors do not use the Dirichlet-multinomial model directly to identify candidate sites for de novo mutations. Instead they augment their estimate of the probability that a site is a de novo mutation with three additional probability calculations: P_noise, P_parent, and P_mosaic. I am not completely clear how these three probabilities are calculated. How are the `k`s for P_noise and P_parent calculated? And is the `m` in P_mosaic the VAF (fraction of non-reference base calls?) for the site? More clarity would improve this part of the paper.

In addition, I think the paper would benefit from an analysis justifying the use of these augmenting factors. My intuition is that some of these values may be double counting things already taken care of in the DMM model, and a comparison of unaugmented and augmented scores would help motivate the additional complexity.

We have revised this section to make the formulas more explicit and to provide additional intuition for the penalty terms.

In brief:

- the noise term is important at noisy or apparently multiallelic sites, where none of the diploid genotype configurations provides a good description of the observed reads. Because the DMM probability is normalized over the set of candidate diploid genotypes, the best-supported genotype may still receive a high relative probability even when its absolute fit to the data is poor. The noise term addresses this by downscaling sites with substantial read support for alleles that are incompatible with the selected genotype.

- the parental alternate-allele support term downscales candidates for which the putative de novo allele is supported by reads in either parent, and does so more strongly when such support is present in both parents.

- the mosaicity term allows to control the baseline variant allele fraction in the child and helps to reduce noise when set stricter.

These additional terms can be disabled via the command line if desired.

VRFS.

Powered by Editorial Manager® and ProduXion Manager® from Aries Systems Corporation

	My understanding of VRFS is that there is a reference histogram constructed from "good" sites that describes how often an individual's VAF falls into a frequency bin. If a de novo is called at site X, then the VRFS measures how well the VAF pattern of site X fits into the reference histogram.<br><br>VRFS is approximately a transformed log-likelihood of the observed bin proportions under the assumption that the proportions of empirical VAFs are independent normal variables with mean 0, and sd sigma_i. It took me a while to fully understand this formation. I think that this is because of the use of \`f\` to represent the fraction of VAFs in a bin, and I kept interpreting it as representing a VAF frequency. Maybe using \`p\` or \`q\` for proportion would be better here? The calculation is also a bit confusing because \`bar(f)_i\` is calculated for the reference and used when estimating sigma^2, but is also assumed to be zero when calculating VRFS. I recommend the authors tweak this description to improve clarity.<br><br>However, wouldn't using the reference bin proportions directly in a goodness-of-fit test be more straight forward than the current VRFS statistic? I think that a Pearson's chi-square test, a G-test, or similar test would be appropriate here to test the hypotheses that the observed frequency bins have the same distribution as a reference set. Is there any reason to suspect that VRFS would perform better or be easier to calculate than a more well known GOF tests?<br><br>Thank you for pointing out that the original explanation was unclear. Your understanding is correct, and we have revised the section to distinguish more clearly between individual-level VAFs and the site-level proportions of samples falling into each VAF bin.<br><br>Regarding the suggested Pearson's chi-square, G-test, or related goodness-of-fit approaches, we agree that these would be reasonable alternative ways to summarize departures from an expected reference distribution. However, VRFS was intended as a simple recurrence score rather than as a calibrated hypothesis test. Specifically, our aim was to penalize candidate sites at which unrelated reference individuals show recurrent non-zero VAF, with each VAF bin weighted by its empirical variability estimated from high-confidence sites.<br><br>A standard goodness-of-fit test would require expected counts for all VAF bins. In this setting, the expected counts in non-zero VAF bins are close to zero, so the test would either be degenerate or would require arbitrary pseudocounts. VRFS avoids this by treating non-zero-bin signal as a recurrence score scaled by empirical bin-specific variability.
**Additional Information:**	
**Question**	**Response**
Are you submitting this manuscript to a special series or article collection?	No
**Experimental design and statistics**<br><br>Full details of the experimental design and statistical methods used should be given in the Methods section, as detailed in our Minimum Standards Reporting Checklist. Information essential to interpreting the data presented should be made available in the figure legends.	Yes

Have you included all the information requested in your manuscript?	
**Resources**<br><br>A description of all resources used, including antibodies, cell lines, animals and software tools, with enough information to allow them to be uniquely identified, should be included in the Methods section. Authors are strongly encouraged to cite Research Resource Identifiers (RRIDs) for antibodies, model organisms and tools, where possible.<br><br>Have you included the information requested as detailed in our Minimum Standards Reporting Checklist?	Yes
**Availability of data and materials**<br><br>All datasets and code on which the conclusions of the paper rely must be either included in your submission or deposited in publicly available repositories (where available and ethically appropriate), referencing such data using a unique identifier in the references and in the “Availability of Data and Materials” section of your manuscript.<br><br>Have you have met the above requirement as detailed in our Minimum Standards Reporting Checklist?	Yes
GigaScience has policies and guidelines in place for the use of generative AI-writing tools such as ChatGPT. If you have used such writing tools to assist with writing the manuscript this must be declared and cited in the text. Authors should not list AI-writing tools and other AI-assisted technologies as an author or co-author and should acknowledge that they are fully responsible for text generated or refined by AI-writing tools.<p>	No

A summary of use (particularly in the introduction or among methods) needs to be included at the end of the paper, and the outputs should also be included as a supplementary file hosted in GigaDB or other open repositories. Please read our guidelines for more information. <p><br><br>By submitting to GigaScience, you are aware of the journal's AI-writing tools policy, and if you have declared use of such tools below, you have acknowledged this where appropriate in your manuscript and have made a summary of use and outputs available. <p><br><b>AI-assisted writing tools have been used in the preparation of this manuscript?	

Click here to access/download;Manuscript;main.pdf

Placeholder for OUP logo oup.pdf

Placeholder for journal logo gigascience-logo.pdf

GigaScience, 2024, 1–9

doi: xx.xxxx/xxxx
Manuscript in Preparation
Paper

PAPER

Improved discovery of *de novo* mutations using TrioDNM and VRFS

Petr Danecek[1*], Eugene J Gardner[2], Joanna Kaplanis[1], Matthew E Hurles[1] and Sarah J Lindsay[1]

[1]Wellcome Trust Sanger Institute, Wellcome Genome Campus, Hinxton CB10 1SA, UK and [2]MRC Epidemiology Unit, Wellcome-MRC Institute of Metabolic Science, University of Cambridge, Cambridge, UK

*pd3@sanger.ac.uk

Abstract

Background: Identifying *de novo* mutations (DNM) is an important component of both genetic research studies and clinical diagnostic workflows, but is complicated by distinguishing true mutations from sequencing errors. Likelihood-based error models are more accurate than inferring mutations from genotypes alone but the resulting callsets still have high false positive rates.
Results: We identify that the main source of false positive DNMs comes from the use of genotype likelihoods in an otherwise robust mutational model. To address this issue, we propose two alternative methods which build on an existing DNM calling approach DeNovoGear, but with higher accuracy and no decrease in sensitivity.
Furthermore, we developed a method which collects allele specific frequency profiles in the sequenced cohort from across many unrelated samples and identifies sites that either demonstrate high rates of sequencing and mapping errors, or are unlikely to be clinically significant due to their high recurrence rate.

Key words: De novo mutation; DNM; bcftools

Introduction

De novo mutations (DNMs) are new genetic variants found only in the genome of the child and not in the genome of either biological parent. While a typical healthy human has ~60 DNMs of no known health consequence [1], DNMs are also an important source of morbidity among neurodevelopmental disorder (NDD) patients, with at least 31-40% of NDD patients having a DNM directly causing or contributing to their symptoms [2].

To identify DNMs from next generation sequencing data using parent-offspring trios, standard variant calling workflows are used [3]. In the most basic approach, resulting genotypes are then analysed to identify loci where only the child, and neither biological parent, have a heterozygous genotype. However, despite continuing advances in sequencing technology and variant calling protocols, genotyping errors occur at a much higher rate than true DNMs, which makes the detection of Mendelian inheritance violations at the genotype level impractical due to the high false positive rate. Therefore, sophisticated probabilistic models were developed which employed genotype likelihoods, transmission probabilities, and prior probability of observing a DNM [4, 5, 6, 7, 8]. Even though family-aware genotype likelihood-based methods are significantly more accurate than the basic genotype-based method [9], improvements can be made, which we highlight below.

There are three main modes of false-positive DNM calls. First, inherited variants in the child may be falsely inferred as *de novo* because the variant allele is incorrectly called as homozygous reference in both parents. Second, false variants may be called in the child at sites with unexpectedly high sequencing error rates. Third, true parental alternate alleles may go undetected due to insufficient sequencing depth.

The first issue is often linked to conflicting demands on sensitivity imposed on the input genotype information with respect to the presence of the alternate allele in the child and its absence in the parents. In the child, calling must be reliable yet appropriately selective, effectively preventing sporadic alternate reads caused by sequencing and mapping artifacts from being misinterpreted as genuine DNMs. Yet in the parents, calling must instead be oversen-

Compiled on: May 28, 2026.
Draft manuscript prepared by the author.

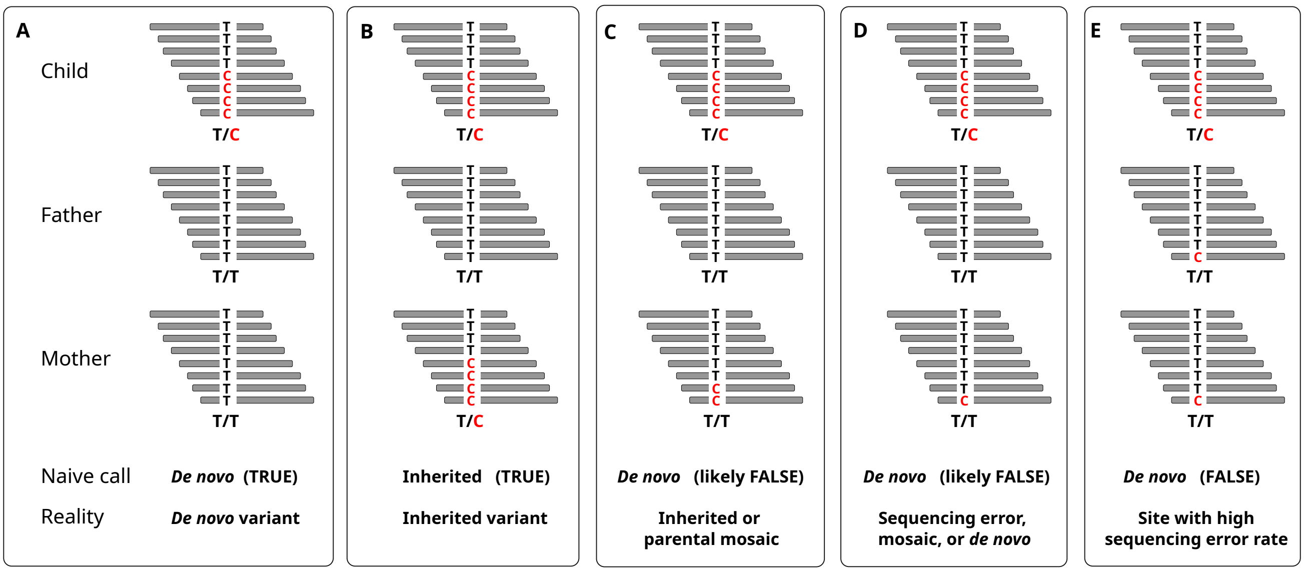

Figure 1. Schematic overview of the core problem of DNM calling. The candidate DNM has the same supporting evidence in the child, but different counts of alternate reads are observed in the parents. The genotypes below each case suggest the most likely genotype as determined by a germline variant caller. Panels **A** and **B** show cases where the ascertainment is trivial. Panels **C**-**E** show the problematic cases where one or both parents have the alternate allele, but the interpretation is uncertain. It can be a misclassified inherited variation **(C)**, sporadic sequencing errors **(D)**, or mapping artefacts **(E)**. Note that although the cases **C** and **D** can also have a valid biological reason, a mosaic post-zygotic parental mutation, the TrioDNM method is not able to distinguish between true parental mosaics and inherited sites where the proportion of the alternate allele in the parent deviates significantly from 50%.

sitive, and highlight the presence of alternate reads in the parents, to prevent an inherited variant from being mistaken for a DNM. These problems are frequently exacerbated by small variations in coverage, number of alternate reads, or mapping and base qualities, and often lead to heterozygous call in the child but homozygous reference calls in both parents (Figure 1). Importantly, the presence of alternate reads in the parents alone is not sufficient evidence for exclusion, because systematic errors (e.g., mapping or alignment artefacts) produce correlated signals across samples, including the child, whereas random sequencing errors are largely independent across individuals. Consequently, excluding all sites with alternate reads in the parents would inflate the false-negative rate for DNMs in the child.

Additional challenges include incorrectly classifying a site as a DNM due to an inherently noisy region with unusually high rate of base miscalls, or failing to detect the parental alternate allele due to insufficient sequencing depth. For example, under a binomial sampling model for reads drawn from a diploid genome, we estimate that approximately seven per thousand heterozygous genotypes will be observed as non-variant sites covered by eight reads. Due to reference mapping bias, this estimate is conservative, particularly for indels, where the bias is more pronounced than for SNVs.

We introduce three methods to improve *de novo* calling and filtering. Methods 1 and 2 extend the genotype likelihood-based model implemented in DeNovoGear [9] by replacing genotype likelihoods with allelic likelihoods, thereby increasing sensitivity to alternate reads in parents. These methods are implemented in the BCFtools/trio-dnm3 plugin and are collectively referred to as TrioDNM. Method 3 analyses the frequency of alternate reads at candidate *de novo* sites in healthy, unrelated parents from the same cohort, enabling detection of false variants at error-prone sites that are difficult to genotype, as well as true variants with high population allele frequencies. This method is implemented in the BCFtools/vrfs plugin and is referred to as VRFS.

Methods

DNG: the DeNovoGear model

The original DeNovoGear model [9], briefly discussed here for reference, identifies DNMs by evaluating joint data likelihoods for all possible combinations of genotypes from mother, father and child (G_M, G_F, G_C) given the observed data (D) as follows

$$L(G_C, G_M, G_F|D) = P(D|G_C, G_M, G_F) \cdot P(G_C|G_M, G_F) \cdot P(G_M, G_F). \quad (1)$$

The first term in Equation 1 is the product of genotype likelihoods in the parent-offspring trio

$$P(D|G_C, G_M, G_F) = P(D_C|G_C) \cdot P(D_M|G_M) \cdot P(D_F|G_F) \quad (2)$$

and they are provided as input to the program. The second term in Equation 1 represents the transmission probability 0.25, 0.5, or 1 for genotypes compatible with Mendelian inheritance, modulated by a generic germline mutation rate $\mu = 10^{-8}$ for each novel allele. Finally, the third term is the prior probability of obtaining the two parental genotypes G_M and G_F from the population under the neutral coalescent model (Supplement S1).

The Equation 1 is evaluated for all possible combinations of genotypes and the most likely combination that is incompatible with Mendelian inheritance is selected. The probability of a variant being a *de novo* mutation is approximated by the posterior probability of the most likely genotype combination that is incompatible with Mendelian inheritance, as follows:

$$P(\text{DNM}) = \frac{L(G_C, G_M, G_F|D)}{\sum_{c,m,f} L(c, m, f|D)}. \quad (3)$$

The summation in the denominator is over all possible genotype combinations, including combinations compatible with Mendelian inheritance.

This model has been reimplemented in the BCFtools/trio-dnm3 plugin and is accessible via the `--use-DNG` command line option (Supplements S1 and S2).

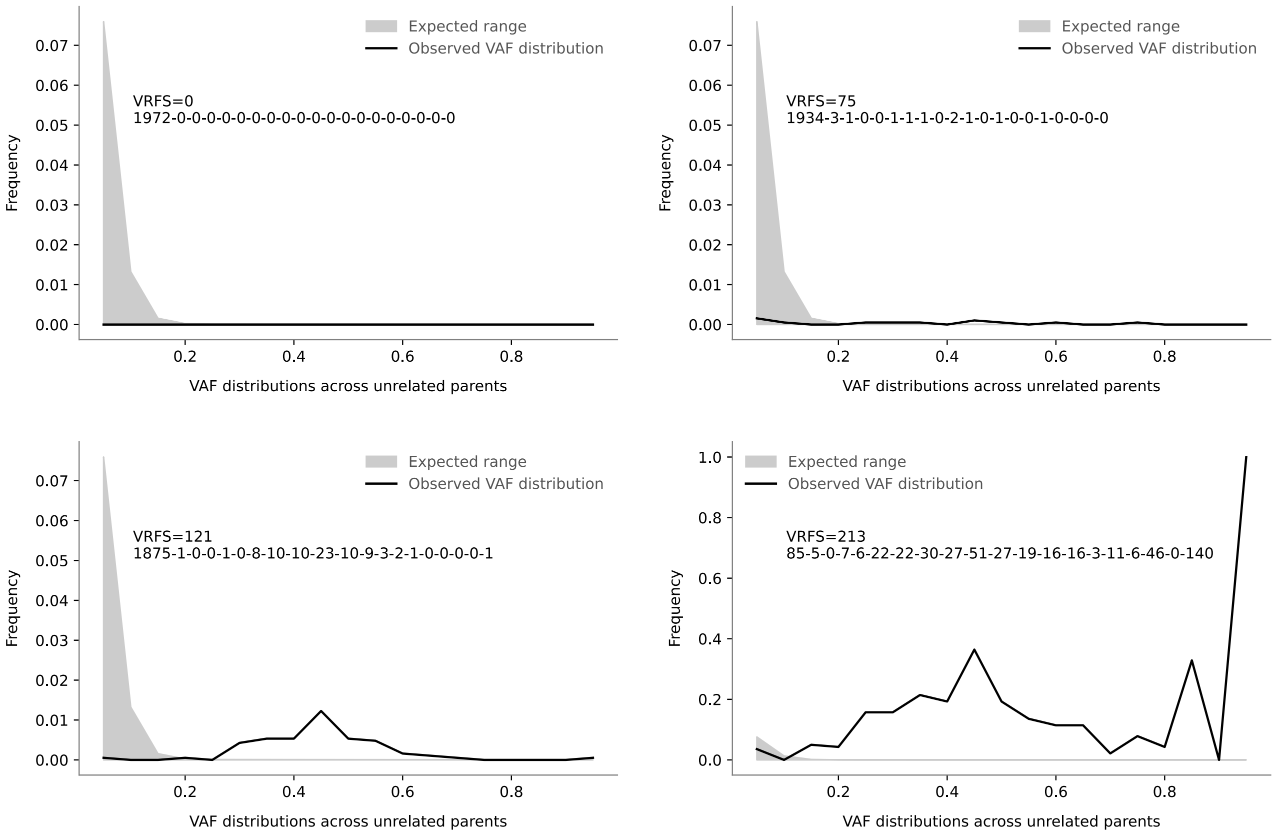

Figure 2. Examples of parental VAF distributions at four sites and the corresponding variant read frequency scores across a range of values, from VRFS=0 (alternate allele not present in the samples) to VRFS=213 (alternate allele is prevalent in the samples, with 140 having alternate homozgyous genotype).

ALM: the allele–likelihood model

The first TrioDNM method, the ALM model, extends DeNovoGear by increasing sensitivity to low-level alternate reads in the parents. This is motivated by the observation that when the proportion of alternate reads deviates substantially from 50%, a heterozygous genotype may appear less likely than a homozygous genotype, even though alternate alleles are clearly present. This occurs because genotype likelihoods are, by design, robust to independent sequencing errors for germline calling, but are less suitable for *de novo* mutation detection, which requires sensitivity to systematic artifacts and low-level mosaicism.

Thus in order to increase the sensitivity to alternate reads, we replace the parental genotype likelihoods in Equation 2 with parental allelic likelihoods:

$$P_{\mathrm{ALM}}(D|G_C, G_M, G_F) = P(D_C|G_C) \cdot A(D_M|G_M) \cdot A(D_F|G_F). \qquad (4)$$

For simplicity of notation, we limit the description to single nucleotide changes, but insertions and deletions are handled identically. We introduce a new variable Q_x which represents the probability that the allele $x \in \{A, C, G, T\}$ observed in a given sample reflects a true signal, rather than a sequencing or mapping artefact. Let $\epsilon_{x,i}$ denote the error probability for the i-th read carrying allele x, defined as the maximum of the corresponding base-calling and mapping error probabilities. Then if the base x is present in reads covering a genomic position k_x times, the probability Q_x can be expressed as

$$Q_x = \begin{cases} 1 - \prod_{i=1}^{k_x} \epsilon_{x,i} & \text{if } k_x > 0, \\ 0 & \text{otherwise.} \end{cases} \qquad (5)$$

The product term corresponds to the probability that all reads carrying allele x can be explained as errors. Thus, Q_x is the complementary probability that the observed support for allele x is not entirely attributable to error, and approaches one when x is supported by high-quality reads. For a true *de novo* allele x, parental Q_x values should be low; elevated values indicate parental support for the allele, consistent with inheritance or systematic mapping bias.

The values Q_x are computed during the BCFtools/mpileup step when the option `--annotate QS` is enabled (Supplement S2). In practice, the calculation is performed in log space to prevent numerical underflow, although this implementation detail is not reflected in the simplified formulation presented here for clarity.

The parental allelic likelihoods $A(D|G)$ in Equation 4 are then calculated separately for the mother and the father in the context of the parental genotype G with alleles a and b as follows:

$$A(D|G = ab) = \prod_x \begin{cases} Q_x & \text{if } x \in \{a, b\}, \\ 1 - Q_x & \text{otherwise,} \end{cases} \qquad (6)$$

where the multiplication is over all bases x observed at the position in the trio. This gives high likelihood when the parental reads strongly support alleles compatible with genotype ab. Conversely, a high-confidence allele inconsistent with ab yields a large Q_x, making $1 - Q_x$ small and penalizing the likelihood. In this sense, the model shifts the focus from genotype inference to evidence evaluation, asking whether allele x is supported by the data rather than how it was generated.

Although ALM is formulated within a principled statistical framework, it combines child genotype likelihoods with parental allele-level likelihoods, resulting in a hybrid model that departs from a fully joint generative formulation of the trio. This model has practical limitations. In particular, the parental emission model for

the candidate *de novo* allele is overly stringent in the presence of random sequencing errors. To mitigate this, we introduce a tolerance parameter allowing a user-controlled fraction of unexpected reads in the parental data. The default tolerance (4.5% when the signal is present in one parent; 1.1% when present in both) were selected empirically from inspection of random set of *de novo* candidates and typical background artefact levels in parental samples. We apply a more stringent threshold when both parents show evidence of the alternate allele, because independent sequencing errors are unlikely to recur at the same site in two separate samples; thus, concordant parental signal more plausibly reflects a systematic site-specific noise than a true *de novo* event.

Another limitation of this approach is that uncertainty arising from low parental sequencing depth is not propagated into the final probability. As a result, sites with limited parental coverage may receive excessively confident *de novo* score despite weak supporting evidence.

Motivated by these considerations, we investigated an alternative model in which parental evidence is modeled directly using a Dirichlet–multinomial distribution over allele counts, replacing the parental allelic likelihood terms $A(D|G)$.

DMM: the Dirichlet–multinomial model

The second TrioDNM method, DMM, was motivated by the observation that although ALM markedly improves upon DNG, it exhibits overconfidence at low parental depth and reduced discriminatory resolution among high-scoring variants, limiting the ability to stratify calls at the upper end of the score distribution. Sensitivity to alternate reads observed in the parents can also be modelled using count-based statistical models. In this approach, we model parental evidence using a three-component Dirichlet–multinomial distribution.

Specifically, for a given parental genotype hypothesis $G = ab$, we summarise the sequencing reads as a count vector (k_a, k_b, k_x), where k_a and k_b are the numbers of reads supporting alleles a and b, and k_x counts all other bases. The corresponding probability vector (p_a, p_b, p_x) is computed from mean per-read base-error probabilities, or from user-specified maximum quality threshold, and subsequently normalized to sum to one. Parental allele counts are then modeled using a Dirichlet–multinomial distribution with concentration parameter ϕ, allowing additional variability in allele counts beyond that expected under multinomial sampling (i.e., overdispersion). The parental allelic likelihood is therefore replaced by

$$A'(D|G = ab) = DM(k_a, k_b, k_x \mid p_a, p_b, p_x; \phi). \tag{7}$$

By default, this replacement is limited to the parental likelihoods. For the child, the original BCFtools/mpileup model is used as before, because it accounts for correlated read errors. In contrast, the Dirichlet–multinomial model summarizes the data as allele counts and does not explicitly model read-level error dependencies. The latter can also be applied to the child likelihood using the `-with-cAD` option.

Like the original DNG model, this approach assumes a complete generative error model in which base and mapping quality scores fully capture sequencing and alignment uncertainty. In practice, this assumption is only partially met: mapping quality reflects how uniquely a read can be placed, but it does not account for sample contamination or mismatches caused by structural variation or alignment ambiguity. Moreover, the framework evaluates only the maximum-likelihood combination of *diploid* genotypes and therefore cannot flag cases in which *none* of the genotype configurations adequately explains the observed data. To address this, we extend Equation 3 by introducing additional penalty terms for read patterns that are unlikely under the selected genotypes. This gives the following augmented probability score:

$$S_{\text{DMM}} = \frac{P_{\text{DMM}}(G_C, G_M, G_F|D)}{\sum_{c,m,f} P_{\text{DMM}}(c, m, f|D)} \cdot P_{\text{noise}} \cdot P_{\text{p\_alt}} \cdot P_{\text{mosaic}}. \tag{8}$$

The first factor is the normalized DMM probability for the selected child–mother–father genotype configuration. The remaining factors penalize specific classes of observations that would be unexpected if this genotype configuration were correct. The noise term

$$P_{\text{noise}} = \prod_{i \in \{C,M,F\}} P_{\text{noise}}(i)$$

is calculated for the child, mother, and father as an upper binomial tail probability

$$P(K \geq k), \quad K \sim \text{Binomial}(n, \epsilon),$$

where k is the number of reads supporting alleles incompatible with the selected genotype in sample i, n is the total number of reads in that sample, and ϵ is the mean per-read error rate. This term downscales sites with read support for more than two alleles, as such patterns cannot be represented by a diploid genotype.

The parental alternate-allele support term

$$P_{\text{p\_alt}} = \prod_{i \in \{M,F\}} P_{\text{p\_alt}}(i)$$

is evaluated for both parents as an upper binomial tail probability, where k is the number of parental reads supporting the candidate *de novo* allele. To account for random sequencing and mapping errors, we introduce a user-controlled tolerance on parental alternate read counts. Up to a specified absolute or fractional number of alternate reads is treated as background noise and excluded from the test; the binomial tail probability is then evaluated on the remaining excess reads only. This treatment and the default threshold values are identical to those used in the ALM method.

Finally, the mosaicity term

$$P_{\text{mosaic}} = P(m \geq m_0 \mid D)$$

is evaluated for the child only. It is the posterior probability that the underlying variant allele fraction m exceeds the user-defined mosaicity threshold m_0, given the observed allele counts. We use a uniform Beta(1, 1) prior on m, giving

$$m \mid D \sim \text{Beta}(1 + k, 1 + n - k),$$

where k is the effective number of reads supporting the candidate *de novo* allele and n is the effective read depth. The effective depth is capped at $50\times$ to prevent overconfidence at very high coverage.

VRFS: the variant read frequency score

Studies of DNMs in large cohorts reveal that some candidate mutations, which initially appear genuine, are also found frequently in unrelated, healthy parents. Such recurrence is unlikely under a true *de novo* model and instead points to systematic technical effects, including dataset-specific artefacts, misalignment, common polymorphisms, or genomic complexity (e.g., paralogous regions). Identifying and downweighting these sites is therefore essential for controlling false discovery rates.

To detect recurrent loci, we developed a method that compares each candidate site against an expected noise profile. This expected profile is generated by collecting the individual-level proportions of alternate reads across a reference set of individuals (parental

samples in our study) and across a set of high-confidence calls, as described below and in Supplementary Figure S3. True DNMs are expected to have very few individuals in the reference set that have a high proportion of variant reads.

For a tested site, we calculate the variant allele fraction (VAF) in each reference individual, defined as the proportion of reads supporting the alternate allele at that site. We then summarize the distribution of these individual-level VAF values across all reference individuals using a histogram with k bins (by default $k = 20$; see Supplementary Figure S4). Let $(f_1, \ldots, f_k)$ denote the resulting empirical VAF distribution, where $f_i = n_i/n$ is the relative frequency of reference individuals in bin i: n_i denotes the number of reference individuals whose VAF falls in bin i, and n denotes the total number of reference individuals.

To estimate the expected variability of these VAF distributions at clean sites, we used a set of m high-confidence calibration sites $\{s_1, \ldots, s_m\}$, in which only a small number of individuals exhibit high VAF values (Supplementary Figures S3C and S5). If $f_{i,s}$ denotes the relative frequency of reference individuals whose VAF falls in bin i at calibration site s, we calculate the variance parameter σ_i^2 as follows:

$$\sigma_i^2 = \frac{1}{m} \sum_{s=1}^{m} \left(f_{i,s} - \bar{f}_i \right)^2 ,$$

where

$$\bar{f}_i = \frac{1}{m} \sum_{s=1}^{m} f_{i,s}.$$

Note that the calibration sites are used to estimate the bin-specific variability of the relative frequencies, rather than to define the null mean used in the score introduced below. Under a true *de novo* model, unrelated reference individuals are expected to have no recurrent alternate-read signal. Therefore, for bins above the lowest-VAF bin, the null expectation at a true *de novo* site is $f_i = 0$ for $i > 1$.

Using this VAF profile, we define the variant read frequency score (VRFS) for a tested site with relative frequencies $(f_1, \ldots, f_k)$ as

$$\mathrm{VRFS}(f_1, \ldots, f_k) = 10 \log \left(1 + \sum_{i=2}^{k} \frac{f_i^2}{\tilde{\sigma}_i^2} \right) , \qquad (9)$$

where

$$\tilde{\sigma}_i^2 = \max(\sigma_i^2, \epsilon^2)$$

for a small constant ϵ, preventing division by zero.

This formulation is motivated by a simple approximate noise model in which the proportion of samples in each VAF bin is treated as an independent normally distributed variable centered at zero, with bin-specific variance estimated from high-confidence sites. The first bin (VAF $\approx$ 0) is excluded because it is dominated by invariant individuals and provides no information about recurrent sequencing noise, given that relative frequencies are normalized. Applying a logarithmic transformation compresses extreme values, stabilizes the statistic's range and prevents outliers from dominating, while adding a constant ensures that sites with no reference individuals outside the first VAF bin receive a score of zero (Figure 2 and Supplementary Figure S6).

Putative DNMs with multiple alternate alleles pose a problem – while multiple alternate alleles observed frequently in unrelated samples is often a hallmark of mapping artefacts in difficult regions, there are also rare cases of genuine DNMs at polymorphic sites. We address this issue differently for SNVs and indels. For multiallelic SNVs, we construct a VAF profile and compute the VRFS value for each alternate allele x separately. The scores are then shifted toward the noisiest allele m according to

$$\mathrm{VRFS}'_x = \mathrm{VRFS}_x + 0.75(\mathrm{VRFS}_m - \mathrm{VRFS}_x). \qquad (10)$$

The coefficient 0.75 was chosen heuristically to ensure that the score of a multiallelic site is largely driven by the noisiest allele while still retaining some contribution from the allele-specific signal. Because insertions and deletions are more difficult to genotype, prone to alignment errors, and often ambiguously aligned, we treat all indels as biallelic regardless of the number of alternate sequences.

A user-friendly, performative version of this model has been implemented in the BCFtools/vrfs plugin (Supplement S2).

Data description

To evaluate the performance of the methods, we used both simulated and exome sequencing data.

Simulated data for evaluation of filtering performance

The initial set comprised 5,000 simulated *de novo* variants; 11 overlapped variants present in parental genomes and were therefore excluded. Reads were then simulated from an artificially constructed trio with the remaining 4,989 variants using Mason [10], and simulated reads were aligned to GRCh38 using BWA-MEM [11]. Subsequent variant calling produced an initial candidate callset with 4,987 variants classified as true positives, 1,039 as false positives, and 2 as false negatives (Supplement S7).

Raw exome callset for evaluation of filtering performance

The wider exome sequencing data generated for 1,094 trios (1,094 children and 1,981 parents) from the Born in Bradford study (BiB). See [12] for details on sample collection and exome sequencing. The Illumina NovaSeq 100bp paired-end reads were aligned to GRCh38 using BWA-MEM. Initial variant calls were made with GATK HaplotypeCaller v4.3.0.0 following GATK best practices [3] and a raw candidate *de novo* callset was generated by selecting sites that do not conform to Mendelian inheritance using the naive function of the BCFtools/trio-dnm3 plugin (Supplement S2).

The initial callset comprised 279,410 candidate variants. To reduce its size prior to downstream analyses, we applied a lenient prefiltering procedure. To avoid inherited variants misclassified as *de novo* due to stochastic binomial sampling of reads from the diploid genome during sequencing, we required a minimum sequencing depth of $10\times$ in all three samples, accounting for difference in inheritance patterns on the sex chromosomes. We further excluded calls located in dense variant clusters, with four or more variants within a 60 bp window. Finally, candidates were ranked separately by each quality score (DNG, ALM, and DMM) using the commands shown in the Supplement S2, and the callset was truncated to guarantee at least five times the expected number of *de novo* calls under any ranking. The expected number was calculated using a mutation rate of $4 \cdot 10^{-8}$ estimated from 2,132 Icelandic families (Supplementary Materials of [13]). The prefiltered candidate *de novo* callset consisted of 11,904 SNVs, 1,781 deletions, and 867 insertions.

Next, to evaluate methods performance, we established a labelled truth set by manual inspection in IGV [14]. Variants were selected by taking the top-ranked calls from each method, ensuring that the highest-scoring candidates under every method were included in the evaluation set. In total, we reviewed 5,958 variants and classified 3,037 (51%) as true positive, 1,562 (26%) as false positive, and 1,359 (23%) as uncertain.

Clean callset for VRFS evaluation

To evaluate the VRFS method, we refined the candidate callset by applying stringent thresholds to all three quality metrics (DNG, ALM, and DMM). Threshold values were determined empirically

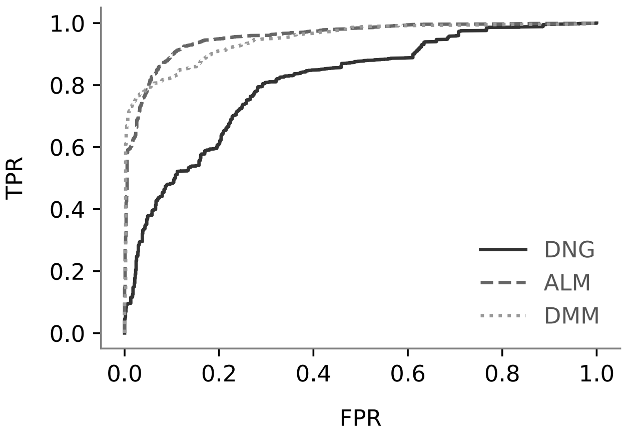

Figure 3. Performance of DNG, ALM, and DMM evaluated on exome sequencing data. Receiver operating characteristic (ROC) curves showing true positive rate (TPR) versus false positive rate (FPR) across varying score thresholds. Areas under the curve (AUC) were 0.808 for DNG, 0.957 for ALM, and 0.947 for DMM. See also Supplementary Figure S8.

using the manually curated sites by inspecting the distribution of true positives (Supplementary Figure S8) and selecting cutoffs that retained 95% of validated true calls. Variants exceeding all three thresholds were retained, subsequently ranked by the selected score, and truncated to a fixed number of top-scoring candidates corresponding to twice the number of expected *de novo* mutations for downstream evaluation.

The callset filtered in this manner contained a significant proportion of duplicate sites, with 19% of SNVs and 5% of indel sites appearing multiple times. These duplicates are likely artifacts rather than genuine DNMs; indeed, 95% of these calls were repeatedly observed among unrelated parents. To highlight the utility of the VRFS method after creating a clean callset using conventional approaches, we removed these duplicates as evident artifacts. This refinement resulted in a filtered callset comprising 2,940 SNVs and 287 indels, which we then used to further investigate the properties of VRFS.

It should be noted that filtering pipelines employed in real-world applications are typically more complex, incorporating additional criteria to optimize both specificity and sensitivity (Supplement S21).

Results

We evaluated DNM callsets generated by applying the proposed methods to both simulated data and exome sequencing data from 1,094 parent–offspring trios. We then investigated the properties and behaviour of the VRFS metric.

Modeling parental allele emission improves filtering performance

We evaluated method performance using three complementary metrics: Ti/Tv, VAF25, and classification performance summarized by the area under the receiver operating characteristic curve (AUC). Ti/Tv, the ratio of nucleotide transitions to transversions, serves as a proxy for biological plausibility, with higher values indicating a cleaner callset (Supplementary Figure S9). VAF25, defined as the proportion of calls with fewer than 25% of reads supporting the alternate allele in the child, captures enrichment of low-allelic-fraction events; lower values indicate a cleaner callset with fewer artefacts (Supplementary Figure S10). ROC curves plot true positive rate against false positive rate and directly quantify trade-offs between sensitivity and specificity (Figure 3).

All three metrics consistently indicate that the TrioDNM methods, ALM and DMM, achieve lower false discovery rates than DNG at comparable levels of sensitivity, suggesting that explicitly modeling parental emission of the *de novo* allele substantially improves filtering performance. Results on simulated trio data as well as manual review of outliers further support this conclusion (Supplementary Figures S7 and S11).

The impact of sequencing depth on performance

One concern when extending the DNG framework was a potential sensitivity to sequencing depth. Although the median minimum depth across trio members in the target regions was 38×, sequencing coverage varies substantially across loci. We therefore assessed how sequencing depth influences method performance by evaluating ROC curves at minimum depth thresholds of 10×, 20×, and 30× (Supplementary Figure S12).

Across all depth cutoffs, the relative ranking of methods remained consistent, with ALM and DMM outperforming DNG. For DNG, the AUC ranged from 0.808 to 0.837, whereas ALM achieved AUCs between 0.957 and 0.981. DMM performed comparably to ALM overall, although at the lowest depth (10×) its performance was slightly reduced relative to ALM (AUC 0.947 vs. 0.957). At 20× and 30×, however, DMM reached the highest accuracy (AUC 0.987) and showed earlier saturation of the ROC curve.

These results indicate that, as expected, sequencing depth influences performance. At low depth (10×), DMM is more conservative method than ALM, likely reflecting its explicit accommodation of potentially missed alternate reads in parents due to binomial sampling. However, DMM reaches ROC saturation earlier than the other methods, indicating more efficient discrimination once sufficient depth is available.

Confluence of TrioDNM and VRFS scores

Considering the size of the sequenced mutational target (~36.7 Mb across 1,094 individuals) and the expected *de novo* mutation rate (~10^{-8} per base per generation), true *de novo* mutations are unlikely to occur at common variant sites or to arise independently in multiple individuals. Genuine recurrent *de novo* events are therefore expected to be orders of magnitude rarer than false positive calls.

The Variant Read Frequency Score (VRFS; Methods) quantifies recurrence of the alternate allele across unrelated samples, enabling identification of loci that most likely reflect sequencing artefacts or, if truly recurrent, are unlikely to be clinically relevant. It is reasonable to expect that calls identified as low quality by TrioDNM methods will often be also identified as low quality by VRFS.

The rankings by DMM and VRFS showed a moderate negative monotonic association in both the broader exome callset (11,904 SNVs) and the refined callset (2,940 SNVs) (Kendall's τ_b = -0.37 and -0.42, respectively). ROC analysis further revealed that VRFS can also distinguish true from false positive calls, although less effectively than the TrioDNM scores, as expected (Figure 4).

The proportion of recurrent and highly recurrent sites (VRFS≥100 and VRFS≥150, respectively), which can be viewed as a proxy for artefactual burden, increases with callset size for both SNVs and indels. However, the magnitude of this effect differs markedly between variant classes. At the expected size of the true *de novo* callset, only 2.9% of SNVs are recurrent (VRFS≥100), whereas approximately 40% of indel sites are recurrent (Supplementary Figure S13). This confirms that indel calls are disproportionately affected by systematic recurrence, consistent with technical challenges associated with repetitive sequence contexts and alignment ambiguity.

Together, these results indicate that sites with large VRFS values and low TrioDNM scores are enriched for artefacts. Both capture different aspects of sequencing noise: VRFS reflects the recurrence of variant reads across unrelated samples, whereas TrioDNM evaluates the likelihood of the variant under a trio inheritance model. Because these metrics rely on distinct signals and data patterns, they provide complementary information. Consequently, filter-

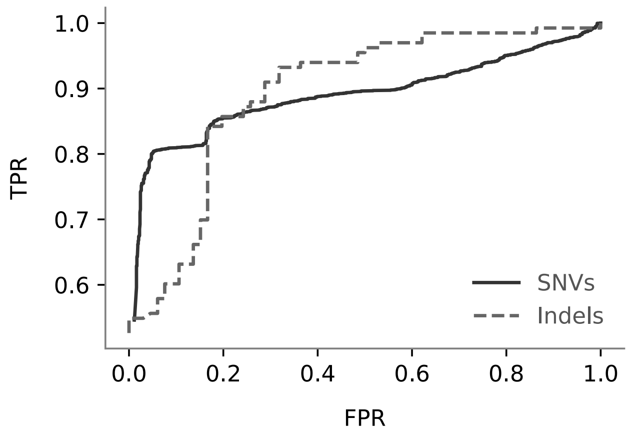

Figure 4. Performance of VRFS in exome sequencing data. ROC curve for VRFS, true positive rate (TPR) is plotted against false positive rate (FPR) across varying VRFS thresholds. Areas under the curve (AUC) were 0.884 for SNVs and 0.894 for indels.

ing candidate *de novo* mutations using both metrics is expected to improve overall callset quality.

Sources of recurrent candidate DNMs

We next examined the genomic correlates of recurrent DNMs. Apparent recurrence may arise from technical artefacts, such as misclassified inherited variants, or from genuine biological processes, including polymerase slippage, gene conversion, and clonal expansion of mutant blood cells. We evaluate both possibilities here.

We anticipate that many recurrent *de novo* sites are within repetitive regions of the genome, leading to reduced alignment quality in short-read sequencing. Indeed, 59% of the highly recurrent SNVs (VRFS$\geq$150) and 57% of the highly recurrent indels in the clean callset are found in paralogs, segmental duplications and repetitive regions (Supplementary Figure S14). The genomic context of recurrent calls differed markedly between SNVs and indels. Among non-recurrent sites (VRFS<50) SNVs were predominantly located in unique sequence, whereas indels already showed substantial enrichment in repetitive regions. With increasing VRFS, the proportion of calls in repetitive regions increased for both variant types, but the pattern of enrichment diverged. Recurrent and highly recurrent SNVs (VRFS$\geq$50 and VRFS$\geq$150) were preferentially located in segmental duplications and paralogous regions, consistent with mapping ambiguity in duplicated sequence. In contrast, recurrent indels were most strongly enriched in simple repeats, with comparatively smaller contributions from segmental duplications. These results suggest distinct mechanisms underlying recurrence: duplicated genomic architecture appears to drive recurrent SNV calls, whereas local sequence repetitiveness plays a dominant role for indels, likely reflecting a combination of polymerase slippage and alignment ambiguity in short-read sequencing.

We also investigated whether stochastic sampling bias in sequencing contributes to the misclassification of inherited variations as DNMs. We found that 0.3% of SNVs and 12% of indels in the clean callset have a significantly higher probability of being misclassified as *de novo* due to failure to sample the alternate allele in the parents at low sequencing depth ($p \geq 10^{-4}$, binomial probability). The prevalence of such sites strongly correlates with the VRFS value. Notably, this effect is dependent on the scoring method used, with ALM substantially over-scoring sites with low parental depth (Supplementary Figure S15).

Regardless of the mechanism, it is reasonable to expect that both true common variants miscalled as DNM and false variants miscalled due to high error rate sites, are also likely to be present in gnomAD [15]. Indeed, in the clean callset, 45% of sites with a *de novo* SNV allele and 68% of sites with a *de novo* indel allele were found also in gnomAD as a polymorphic site, of which 9% and 29%, respectively, had allele frequency (AF) greater than 0.1 (Supplementary Figure S16). The majority of these sites, with AF>0.1, were also highly recurrent in unrelated samples in our study, i.e. 82% of these SNVs and 91% of indels had VRFS$\geq$150.

While we observed that large VRFS values predominantly highlight misclassified inherited variation (such as in Supplementary Figure S17) and false positives in difficult-to-align regions (such as in Supplementary Figure S18), it is possible that genuine highly recurrent somatic mutations could also be present among the candidate DNMs with high VRFS scores. For completeness, we provide the list of recurrent sites identified in this study, although it was derived from a single cohort and its generalizability to other datasets has not been evaluated (Supplement S19).

The stability of the VRFS method

A set of reference samples is required to generate VAF profile distributions. The sensitivity of the method to common variation and artefacts increases with the number of samples. Reducing the number of samples by 75% (from 1,981 to 500) reduces the sensitivity by 0.2% (i.e. 0.2% of recurrent sites with VRFS$\geq$100 are newly reported as non-recurrent with VRFS<50) and limiting the number of samples by 95% (from 1,981 to 100) reduces the sensitivity by 1.7% (Supplementary Figure S20). This suggests the method can be used with relatively small cohorts – parental samples of as few as 50 trios can capture 98.7% of problematic sites.

Note that the method is also stable with respect to the bin size (by default $k = 20$) and the values remain highly correlated over a range of bins (Supplementary Figure S4).

Discussion

We developed three related methods to assist in the calling and filtering of *de novo* SNVs and short indels. Two of these approaches are complementary in their modeling assumptions, and one is implemented in two alternative formulations. All methods were implemented as standard BCFtools plugins with usability and computational efficiency in mind.

The first tool, TrioDNM, is designed for use in parent–offspring trios and addresses key limitations of existing approaches. Prior methods rely primarily on parental genotype likelihoods to evaluate the presence of the putative *de novo* allele. In contrast, we implemented two alternative strategies that leverage allelic likelihoods evaluated conditional on the child's genotype. We demonstrate that both ALM and DMM scores substantially improve the ranking of *de novo* candidates and yield a more refined callset compared to DNG, the previous formulation of the model. Overall, DMM provides the most accurate discrimination and, relative to ALM, appropriately accounts for uncertainty arising from low parental sequencing depth.

The second method, VRFS, was developed for DNM calling but can have broader utility for other applications, e.g. generating high quality rare SNV/indel callsets in duos (a child plus one biological parent) and other non-trio data. The method collects allelic frequency profiles from many samples, enabling the filtering of sites with uncertain clinical relevance, such as misclassified inherited variants or sites that are difficult to genotype. Using the VRFS method, we found that 3% of apparent *de novo* SNVs and 40% of indels are also present as variants in unrelated samples. This observed recurrence is likely attributable to several factors, with distinct patterns for SNVs and short indels. Recurrent candidate *de novo* SNVs were more prevalent in segmental duplications and paralogous regions, whereas recurrent indels occurred predominantly in simple repeats and SINE elements. These contrasting patterns likely reflect different underlying mechanisms. In duplicated and paralogous regions, recurrent SNV calls may arise from

mapping ambiguity. In contrast, the enrichment of indels in simple repeats is consistent with polymerase slippage, which may occur both biologically during DNA replication and technically during PCR amplification and sequencing. Furthermore, the degree of recurrence strongly correlates with stochastic sampling bias, where lower sequencing depth in the parents increases the likelihood of missing the alternate allele.

The recommended approach for utilizing these methods is to rank candidate calls by the DMM score, identifying variants that appear to be genuine DNMs within the parent-offspring trio. Subsequently, pooled information from multiple samples can be used to filter based on the VRFS value, effectively eliminating common artifacts and inherited variation. Note that a real-world filtering pipeline is typically more complex and incorporates additional filtering criteria (see an example in Supplement S21). The two methods presented here are specifically designed to address only a subset of the failure modes observed in sequencing data.

While public genomic resources like gnomAD could serve the same purpose for filtering out common variants, they cannot account for cohort-specific sequencing artifacts, or may not be available for the studied organism or reference build. Our findings show that relatively small sample sizes are sufficient to capture the majority variation—for instance, VRFS applied on 100 samples could identify 98.7% of recurrent sites. Also, while the calculation of VRFS values is sensitive to VAF profile variances $\{\sigma_i^2\}$, which are precomputed from a set of high-confidence sites, we show that these error profiles can be reused between studies (Supplementary Figure S5).

The accuracy of the TrioDNM method is contingent upon the quality of its input data, namely genotype and allelic likelihoods. These inputs are frequently subject to inaccuracies, particularly in the case of indels, which are notoriously challenging to genotype accurately. However, the model could be improved—in theory both DeNovoGear and TrioDNM models could include allele specific mutation rates to account for known effects such as 5-Methylcytosine at a CpG being more prone to transition than unmethylated cytosine due to spontaneous deamination. Furthermore, while the VRFS method takes into account multiallelic SNVs individually, its treatment of indels is limited to a site-level assessment.

In general, the methods are not suitable for all forms of genetic variation, such as copy number variation (CNVs). Even though the genotype and allelic likelihoods, which serve as input data to the model, could be in principle provided for any variant type, in practice their accuracy is not sufficient to produce reliable results. Furthermore, while TrioDNM can detect DNMs at common polymorphic sites in the population which modify the alternate allele to reference (i.e. 0/1 to 0/0), the VRFS method will recognize such sites as common polymorphism and a site that falls in this category will receive a high VRFS score.

Data availability

This study reuses whole-exome sequencing data from the Born in Bradford (BiB) birth cohort, generated as part of the UK birth-cohort exome sequencing resource described by Koko et al. [12]. The BiB data are available to approved researchers through the European Genome–phenome Archive under study accession EGAS00001006978 and dataset accession EGAD00001015370.

Access to BiB data is controlled to protect participant confidentiality. Researchers can apply by submitting an Expression of Interest to the BiB team; approved applications are subject to BiB governance review and completion of the required data access agreements.

The data underlying all figures are provided in Supplementary File `supporting-data.zip`. A description of the contents of this archive is provided in Supplement S22.

Availability of Source Code and Requirements

Project name: TrioDNM and VRFS
Project home page:
https://github.com/HurlesGroupSanger/trio-dnm-calling
https://github.com/samtools/bcftools
License: MIT license
Operating system(s): Platform independent
Programming language: C, perl
Software dependencies: BCFtools

See also Supplements S2 and S21.

Acknowledgements

We thank the reviewers for their comments and suggestions that helped improve the manuscript and motivated the development of additional methodological extensions presented in this work.

This work was supported by Wellcome Grant reference number 220540/Z/20/A, Wellcome Sanger Institute Quinquennial Review 2021-2026. For the purpose of Open Access, the author has applied a CC BY public copyright license to any Author Accepted Manuscript version arising from this submission

This study makes use of data from the Born in Bradford programme, which is only possible because of the enthusiasm and commitment of the children and parents in BiB. We are grateful to all the participants, health professionals, schools and researchers who have made Born in Bradford happen.

EJG is an employee of and holds shares in Insmed, Inc.

References

1. Kaplanis J, *et al*. Genetic and chemotherapeutic influences on germline hypermutation. Nature 2022;605:503–508. doi:10.1038/s41586-022-04712-2.
2. Wright C, *et al*. Genomic Diagnosis of Rare Pediatric Disease in the United Kingdom and Ireland. N Engl J Med 2023;388(17):1559–1571. doi:10.1056/NEJMoa2209046.
3. van der Auwera G, O'Connor BD. Genomics in the Cloud: Using Docker, GATK, and WDL in Terra. O'Reilly Media, Incorporated; 2020.
4. Ramu A, *et al*. DeNovoGear: de novo indel and point mutation discovery and phasing. Nat Methods 2013;10(10):985–7. doi:10.1038/nmeth.2611.
5. Wei Q, Zhan X, Zhong X, Liu Y, Han Y, Chen W, et al. A Bayesian framework for de novo mutation calling in parents-offspring trios. Bioinformatics 2014 12;31(9):1375–1381. doi:10.1093/bioinformatics/btu839.
6. Francioli LC, *et al*. A framework for the detection of *de novo* mutations in family-based sequencing data. Eur J Hum Genet 2017;25:227–233. doi:10.1038/ejhg.2016.147.
7. Kolesnikov A, Goel S, Nattestad M, Yun T, Baid G, Yang H, et al. DeepTrio: Variant Calling in Families Using Deep Learning. bioRxiv 2021;doi:10.1101/2021.04.05.438434.
8. Khazeeva G, Sablauskas K, van der Sanden B, Steyaert W, Kwint M, Rots D, et al. DeNovoCNN: a deep learning approach to de novo variant calling in next generation sequencing data. Nucleic Acids Research 2022 06;50(17):e97–e97. doi:10.1093/nar/gkac511.
9. Conrad DF, *et al*. Variation in genome-wide mutation rates within and between human families. Nat Genet 2011;43(7):712–714. doi:10.1038/ng.862.
10. Holtgrewe M. Mason – A Read Simulator for Second Generation Sequencing Data. Technical Report FU Berlin 2010 October;.
11. Li H. Aligning sequence reads, clone sequences and assembly contigs with BWA-MEM. ArXiv 2013;26 May.
12. Koko M, Fabian L, *et al*. Exome sequencing of

UK birth cohorts. Wellcome Open Res 2024;9:390. doi:10.12688/wellcomeopenres.22697.1.
13. Gunnar Palsson HJ*et al* Marteinn T Hardarson. Complete human recombination maps. Nature 2025;639:700–707. doi:10.1038/s41586-024-08450-5.
14. Thorvaldsdóttir H, *et al*. Integrative Genomics Viewer (IGV): high-performance genomics data visualization and exploration. Briefings in bioinformatics 2013;14,2:178–192. doi:10.1093/bib/bbs017.
15. Chen S, Francioli LC, Goodrich JK, *et al*. A genomic mutational constraint map using variation in 76,156 human genomes. Nature 2024;625:92–100. doi:10.1038/s41586-023-06045-0.

Click here to access/download
Supplementary Material
supplement.pdf
