# Supplementary material for: Improved discovery of de novo mutations using TrioDNM and VRFS
Source: Gigascience. 2026 Jun 9;15:giag068. doi: 10.1093/gigascience/giag068 (PMC13289757; doi:10.1093/gigascience/giag068)
Supplement: giag068_Authors_Response_To_Reviewer_Comments_original_submission [file giag068_authors_response_to_reviewer_comments_original_submission.pdf]

Author's Response To Reviewer Comments

We thank the reviewers for their helpful comments and suggestions. In response, we have revised and significantly extended the methods, analyses, and the manuscript. Detailed responses to each comment are provided below.

Reviewer #1: De novo mutations play a crucial role in genetic and trait evolution. Danecek et al. introduced two methods to enhance de novo mutation (DNM) calling and filtering. Generally, this paper is well-organized and written, presenting valuable methods for improving DNM calling.
I have a few suggestions or concerns that may help strengthen the work:

1.It was observed that the sequencing depth distribution in the BiB cohort exhibits two peaks (~13-fold, ~53-fold; Supplementary Fig. S7). Therefore, it is recommended that the authors evaluate the impact of sequencing depth on filtering performance.

We evaluated the impact of sequencing depth on filtering performance and added a new section, “The impact of sequencing depth on performance,” where this analysis is presented.

2.All analyses were based on exome sequencing data (~37-Mb target regions). However, whole-genome sequencing (WGS), typically using next-generation sequencing (NGS), has become a common approach for profiling de novo mutations. If high-quality public trio NGS data are available, it is suggested that the performance of TrioDNM and VRFS in non-coding regions be assessed using WGS data.

Evaluating performance using WGS data would indeed be valuable. While we don't expect the methods to perform differently in coding and non-coding regions, we extended our analysis to include simulated WGS data, where the status of each variant is known and method performance therefore can be assessed in a controlled manner across the genome.

3.There is confusion regarding the call sets evaluated in different figures and supplementary figures. In the methods section, an initial call set (72k SNVs and 38k InDels), a prefiltered call set (21k SNVs and 9k InDels), and a manually curated call set were mentioned. It remains unclear whether the call sets used in Fig. 3 and Fig. S9 are the initial call sets. Additionally, Fig. S10 displays 60k InDels, which is inconsistent with the initial call set containing only 38k InDels. Thus, the authors are advised to supplement more detailed legend information for the figures.

Thank you for noticing this inconsistency which was indeed confusing. To resolve this, we simplified the analysis and regenerated the callsets from scratch, which also enabled evaluation of an updated version of the method motivated by comments from Reviewer #2. All affected figures and legends have been revised to clearly indicate the call sets used.

4.This study evaluated recurrent de novo sites with high frequency, which may be misclassified. These site coordinates are of great significance for research on human de novo mutations in genetics and medical diagnosis. It is suggested that the authors deposit this dataset as a supplementary table or in a public repository.

We have included a subset of highly recurrent sites identified in this study as a supplementary table for reference.

5.It is recommended that the authors add usage examples, such as those shown in Supplementary S2, to the GitHub homepage.

We have added a link to the documentation, which includes usage examples, to the GitHub homepage.

minor:
1.Regarding the title: The core of this study lies in the development of two complementary approaches for de novo mutation filtering. I think current title can not reflect the key points Alternative titles could be considered for reference, such as "Quality control of de novo mutation discovery using TrioDNM and VRFS" or "Refining de novo mutations using TrioDNM and VRFS".

Thank you for your suggestion. We have changed the title to “Improved discovery of de novo mutations using TrioDNM and VRFS”, which we agree is more informative and accurately reflects the scope of the manuscript.

2.In the data description: the notation "38x" should be standardized to "38×" or "38-fold". This correction should also be applied to Supplementary Fig. S7 and other related statements.

This is corrected, thank you.

3.Maximum value displayed in Fig 2 is 213, while the caption indicates 215.

This is corrected, thank you.

Reviewer #2: Danecek et al. propose two new methods to improve de novo mutation calling. The first method, allelic likelihoods, makes calling more sensitive to alternative reads in the parents. The second method, VRFS, attempts to identifying sites that are challenging to genotype, reducing false positive calls.

While I think that both proposed methods are important developments for the field, I often found the paper hard to follow as there were many sections that were missing details and justifications. I often found myself struggling to understand how results were generated and how I should understand them. The paper would be improved if the authors spent a bit more time clarifying its technical aspects both in the text and in the figures and figure legends.

STRENGTHS

Calling denovo mutations (DNMs) is a genomic and bioinformatic challenge because many genomic error rates are often at the same or greater magnitude as the rate at which DNMs occur. The authors correctly explain that the biggest sources of false positive DNM calls originate from calling a heterozygous parent as homozygous due to low coverage and calling a homozygous child as heterozygous due to contamination or difficult to align locations.

In response to these challenges, the authors have developed two novel methods to identify denovo mutations and filter out potentially problematic sites. The new methods are statistical in nature which is important because statistical approaches for finding denovo mutations have long been superior to more commonly used ad hoc and heuristic approaches.

The new methods were tested on genomic data from the Born-in-Bradford study using both a manually curated truth dataset as well as biological signals to demonstrate the quality of their approaches. The results clearly show that the new methods are an important contribution to the field.

Since generating large, high-quality truth datasets using real data is difficult and time consuming, the authors also looked at whether the output of their methods made sense biologically. This is a powerful technique for validating methods that is often overlooked.

WEAKNESSES

As mentioned above, the biggest weakness in this paper is the lack of clarity about the technical aspects. I noticed several typos and omissions in the equations. Since this is a methods paper, the description of the methods should be well polished and unfortunately they are not at this time.

We have carefully revised and extended the methodological description throughout the manuscript to improve clarity and completeness. In particular, we corrected typographical errors in the equations and clarified several technical details.

The methods are validated on a small truth set of 374 manually curated variants. This seems to me to be a really small number and as the authors admit, potentially biased. For reasons such as these, most new methods are evaluated on both simulated and empirical datasets. While no simulated data is as messy as real data, simulations are still useful for evaluating methods. The paper would be improved if the authors tested their methods on a simulated dataset in which the truth is known. This could be accomplished by completely simulating data or introducing simulated mutations into their dataset. They could also down-sample high-quality validated datasets like Platinum Genomes to validate their methods.

We have extended the curated truth set to more than 5,000 sites, substantially increasing the number of manually evaluated variants. In addition, we now include analyses based on simulated data to further assess method performance when the ground truth is known. We also considered using Platinum Genomes; however, this dataset contains only a small number of trios and the variants were not called jointly within families, which limits its suitability for evaluating trio-based de novo calling methods.

TrioDNM model

The quantity Qx where `x` is {A, C, G, or T} estimates the probability that at **least one base call of `x` is not an error**. This is then used in various ways to calculate allelic likelihoods. This is a big change from using a multinomial or Dirichlet-multinomial distribution for genotype likelihoods, and I think it deserves more discussion and justification than is currently in the paper.

We have revised this section to clarify the description of the model and to provide additional intuition for its formulation. We also simplified the formulation of the model. Furthermore, inspired by the reviewer’s comment, in addition we implemented a Dirichlet–multinomial (DM) model and included it in the analyses. While this model required additional considerations in implementation, it addresses an important limitation of the original TrioDNM model—its overconfidence at low parental depth. In fact, it is now used as the default calling method.

Equation (6). This equation does not explain what happens when a != b and x is not in {a,b,c,d}. From looking at the source code it appears that it uses `1` for this final case. This should produce some weird likelihoods such that the probability of Dm = {A, A, A, A} given Gm = {C, G} and Gc = {G, G} is higher than Dm = {A, A, A, A} given Gm = {A, A} and Gc = {G, G}. While this may not matter when identifying the most likely mutation pattern, it should definitely affect how the calculation of equation (3). If the authors are using a trick to avoid this, they should clarify that in the paper.

Thank you for noticing. We have revised and fixed this issue.

VFRS

I have no statistical institution about what VRFS represents. I don't understand how the f_i's or sigma_i's were calculated or why they are squared. I also don't understand why there is a `1 +` in the calculation as well. As such, I have a hard time understanding how VRFS should work. The authors should include more

motivation and explanation for the VRFS statistic.

We have expanded the description of the VRFS statistic to provide additional motivation and intuition for the formulation. In particular, we clarified how the f_i and sigma_i values are computed and added an explanation of the rationale for the squared terms and the 1+ term in the formula.

Other Weaknesses

The DNG and TDNM scores are not defined, which makes interpreting the results difficult. They appear to be log-probabilities, but the paper also describes them as bins. The authors need to clarify how the scores are calculated and used.

The program allows selecting how these scores are reported using the --dnm-tag option: as probabilities (0–1; float), log-scaled values (−∞ to 0; float), or Phred-scaled values (0–255; integer). By default, log-scaled values are used, as they are the most practical representation for the typical range of scores.

The manuscript does not describe how DNG and TDNM models were parameterized. The authors should discuss whether changing the values of parameters used in DNG and TDNM models would change the results of the model.

We added description of parameters and their values where appropriate.

The paper claims that VRFS and TDNM scores are not correlated and provide complementary information. I am not sure how this conclusion was made. The authors could create a scatter plot of TDNM vs VRFS to justify this claim.

We agree that the original wording was unclear. Our intention was not to make a claim about statistical correlation of VRFS and TrioDNM, but rather that they capture different types of signal: VRFS reflects the recurrence of variant reads across unrelated samples, while TrioDNM evaluates the likelihood of a variant under a trio inheritance model. Because they rely on different information, they can flag artefacts through distinct mechanisms and therefore provide complementary information. We have revised the text to clarify this point.

OTHER COMMENTS

Equation (1). This equation contains a typo. It should be

`L(Gc, Gm, Gf | D) = P(D | Gc, Gm, Gf) x P(Gc | Gm, Gf) x P(Gm, Gf)`

where the second term on the right has the child's genotype in it.

The reviewer is correct; this was a typographical error. Thank you for noticing. The equation has now been corrected in the manuscript.

Equation (3). The authors describe this as the posterior probability of the variant being a denovo mutation. This is not accurate. Equation (3) is the probability of the most likely genotype combination that is incompatible with Mendelian inheritance (given observed data). The probability that there is a denovo mutation in the child given data is
P(DNM | D) = sum(P(DNM | D, Gc, Gm, Gf) * P(D, Gc, Gm, Gf)) / sum(P(D, Gc, Gm, Gf))
where the numerator is also calculated by summing over possible genotype combinations. For example,

DeNovoGear's `call` algorithm calculates this statistical accurately while it's `dnm` algorithm generates one like in this paper.
And finally, I think that the probability of a variant being a DNM (as used in the text) would be calculated by fixing the genotype of the child and summing over the parents.
P(DNM | D, Gc = g) = sum(P(DNM | D, Gc = g, Gm, Gf) * P(D, Gc = g, Gm, Gf)) / sum(P(D, Gc = g, Gm, Gf))
To be clear, there is nothing wrong about Equation (3); however, the text doesn't accurately describe it.

We agree that the original description was imprecise. The text has been revised to clarify that the probability of a variant being a de novo mutation is approximated by the posterior probability of the most likely genotype combination that is incompatible with Mendelian inheritance. The revised text now reads:
The probability of a variant being a de novo mutation is approximated by the posterior probability of the most likely genotype combination that is incompatible with Mendelian inheritance, as follows: [...]

Equation (5). The quantity Qx is described as the "sum of base/mapping qualities in log space". This is not correct. The relationship between Qx and the sum of base/mapping qualities (QS[x]) is Qx = 1 - 10^(-QS[x]/10). This should be corrected, or replaced by a statement that Qx can be calculated from QS.

We agree that our verbal description was confusing. We have revised the text to clarify the definition. In our formulation, ε_i denotes the probability of an error for the i-th read. In practice, we obtain ε_i from phred-scaled base or mapping quality as ε = 10^(-BQ/10). The Equation 5 now correctly states Qx = 1 - \prod ε_i. Intuitively, even a single high-quality supporting reads (small ε_i) is sufficient to make Qx large. We have clarified in the text that although the practical calculation is performed in log space to prevent numerical underflow, the formulation presented in the manuscript uses plain probabilities for clarity.

Equation (6). I believe that only the reads from a mother or father are used to calculate Qx. The authors should clarify this by using notation for individual specific datasets in the paper. For example, if D = {Dm, Df, Dc}, then equation (6) could be written as A(Dm | Gm = ab, Gc = cd) for the mother's allelic likelihood.

During the revision, we realized that the original formulation was unnecessarily complicated. We therefore simplified and clarified the text.

Editorial Manager®

Cookie Preference Center

We use cookies which are necessary to make our site work. We may also use additional cookies to analyze, improve and personalize our content and your digital experience. For more information, see our Cookie Policy.

You may choose not to allow some types of cookies. However, blocking some types may

impact your experience of our site and the services we are able to offer. See the different category headings below to find out more or change your settings.

Allow all

Manage Consent Preferences

Strictly Necessary Cookies

Always active

These cookies are necessary for the website to function and cannot be switched off in our systems. They are usually only set in response to actions made by you which amount to a request for services, such as setting your privacy preferences, logging in or filling in forms. You can set your browser to block or alert you about these cookies, but some parts of the site will not then work.

Functional Cookies

These cookies enable the website to provide enhanced functionality and personalisation. They may be set by us or by third party providers whose services we have added to our pages. If you do not allow these cookies then some or all of these services may not function properly.

Performance Cookies

These cookies allow us to count visits and traffic sources so we can measure and improve the performance of our site. They help us to know which pages are the most and least popular and see how visitors move around the site.

Targeting Cookies

These cookies may be set through our site by our advertising partners. They may be used by those companies to build a profile of your interests and show you relevant adverts on other sites. If you do not allow these cookies, you will experience less targeted advertising.

Cookie List

Clear

Apply Cancel

Consent Leg.Interest

Confirm my choices

Power
