# Supplementary material for: Improved discovery of de novo mutations using TrioDNM and VRFS
Source: Gigascience. 2026 Jun 9;15:giag068. doi: 10.1093/gigascience/giag068 (PMC13289757; doi:10.1093/gigascience/giag068)
Supplement: giag068_Reviewer_1_Report_original_submission — Reviewer 1 -- 8/19/2025 [file giag068_reviewer_1_report_original_submission.pdf]

Reviewer Report

Title: Improved discovery of de novo mutations using TrioDNM and VRFS

Version: Original Submission **Date:** 8/19/2025

Reviewer name: Reviewer 1

Reviewer Comments to Author:

De novo mutations play a crucial role in genetic and trait evolution. Danecek et al. introduced two methods to enhance de novo mutation (DNM) calling and filtering. Generally, this paper is well-organized and written, presenting valuable methods for improving DNM calling.
I have a few suggestions or concerns that may help strengthen the work:
1.It was observed that the sequencing depth distribution in the BiB cohort exhibits two peaks (~13-fold, ~53-fold; Supplementary Fig. S7). Therefore, it is recommended that the authors evaluate the impact of sequencing depth on filtering performance.
2.All analyses were based on exome sequencing data (~37-Mb target regions). However, whole-genome sequencing (WGS), typically using next-generation sequencing (NGS), has become a common approach for profiling de novo mutations. If high-quality public trio NGS data are available, it is suggested that the performance of TrioDNM and VRFS in non-coding regions be assessed using WGS data.
3.There is confusion regarding the call sets evaluated in different figures and supplementary figures. In the methods section, an initial call set (72k SNVs and 38k InDels), a prefiltered call set (21k SNVs and 9k InDels), and a manually curated call set were mentioned. It remains unclear whether the call sets used in Fig. 3 and Fig. S9 are the initial call sets. Additionally, Fig. S10 displays 60k InDels, which is inconsistent with the initial call set containing only 38k InDels. Thus, the authors are advised to supplement more detailed legend information for the figures.
4.This study evaluated recurrent de novo sites with high frequency, which may be misclassified. These site coordinates are of great significance for research on human de novo mutations in genetics and medical diagnosis. It is suggested that the authors deposit this dataset as a supplementary table or in a public repository.
5.It is recommended that the authors add usage examples, such as those shown in Supplementary S2, to the GitHub homepage (https://github.com/HurlesGroupSanger/trio-dnm-calling).
minor:
1.Regarding the title: The core of this study lies in the development of two complementary approaches for de novo mutation filtering. I think current title can not reflect the key points Alternative titles could be considered for reference, such as "Quality control of de novo mutation discovery using TrioDNM and VRFS" or "Refining de novo mutations using TrioDNM and VRFS".
2.In the data description: the notation "38x" should be standardized to "38Ã—" or "38-fold". This correction should also be applied to Supplementary Fig. S7 and other related statements.
3.Maximum value displayed in Fig 2 is 213, while the caption indicates 215.

Methods

Are the methods appropriate to the aims of the study, are they well described, and are necessary controls included? Choose an item.

Conclusions

Are the conclusions adequately supported by the data shown? Choose an item.

Reporting Standards

Does the manuscript adhere to the journal's guidelines on minimum standards of reporting? Choose an item.

Choose an item.

Statistics

Are you able to assess all statistics in the manuscript, including the appropriateness of statistical tests used? Choose an item.

Quality of Written English

Please indicate the quality of language in the manuscript: Choose an item.

Declaration of Competing Interests

Please complete a declaration of competing interests, considering the following questions:

- Have you in the past five years received reimbursements, fees, funding, or salary from an organisation that may in any way gain or lose financially from the publication of this manuscript, either now or in the future?
- Do you hold any stocks or shares in an organisation that may in any way gain or lose financially from the publication of this manuscript, either now or in the future?
- Do you hold or are you currently applying for any patents relating to the content of the manuscript?
- Have you received reimbursements, fees, funding, or salary from an organization that holds or has applied for patents relating to the content of the manuscript?
- Do you have any other financial competing interests?
- Do you have any non-financial competing interests in relation to this paper?

If you can answer no to all of the above, write 'I declare that I have no competing interests' below. If your reply is yes to any, please give details below.

I declare that I have no competing interests

I agree to the open peer review policy of the journal. I understand that my name will be included on my report to the authors and, if the manuscript is accepted for publication, my named report including any attachments I upload will be posted on the website along with the authors' responses. I agree for my report to be made available under an Open Access Creative Commons CC-BY license (http://creativecommons.org/licenses/by/4.0/). I understand that any comments which I do not wish to be included in my named report can be included as confidential comments to the editors, which will not be published.

Choose an item.

To further support our reviewers, we have joined with Publons, where you can gain additional credit to further highlight your hard work (see: https://publons.com/journal/530/gigascience). On publication of this paper, your review will be automatically added to Publons, you can then choose whether or not to claim your Publons credit. I understand this statement.

Yes Choose an item.
