# Supplementary material for: Improved discovery of de novo mutations using TrioDNM and VRFS
Source: Gigascience. 2026 Jun 9;15:giag068. doi: 10.1093/gigascience/giag068 (PMC13289757; doi:10.1093/gigascience/giag068)
Supplement: giag068_Reviewer_2_Report_original_submission — Reviewer 2 -- 9/6/2025 [file giag068_reviewer_2_report_original_submission.pdf]

Reviewer Report

Title: Improved discovery of de novo mutations using TrioDNM and VRFS

Version: Original Submission **Date:** 9/6/2025

Reviewer name: Reviewer 2

Reviewer Comments to Author:

Danecek et al. propose two new methods to improve de novo mutation calling. The first method, allelic likelihoods, makes calling more sensitive to alternative reads in the parents. The second method, VRFS, attempts to identifying sites that are challenging to genotype, reducing false positive calls.

While I think that both proposed methods are important developments for the field, I often found the paper hard to follow as there were many sections that were missing details and justifications. I often found myself struggling to understand how results were generated and how I should understand them. The paper would be improved if the authors spent a bit more time clarifying its technical aspects both in the text and in the figures and figure legends.

STRENGTHS

Calling denovo mutations (DNMs) is a genomic and bioinformatic challenge because many genomic error rates are often at the same or greater magnitude as the rate at which DNMs occur. The authors correctly explain that the biggest sources of false positive DNM calls originate from calling a heterozygous parent as homozygous due to low coverage and calling a homozygous child as heterozygous due to contamination or difficult to align locations.

In response to these challenges, the authors have developed two novel methods to identify denovo mutations and filter out potentially problematic sites. The new methods are statistical in nature which is important because statistical approaches for finding denovo mutations have long been superior to more commonly used ad hoc and heuristic approaches.

The new methods were tested on genomic data from the Born-in-Bradford study using both a manually curated truth dataset as well as biological signals to demonstrate the quality of their approaches. The results clearly show that the new methods are an important contribution to the field.

Since generating large, high-quality truth datasets using real data is difficult and time consuming, the authors also looked at whether the output of their methods made sense biologically. This is a powerful technique for validating methods that is often overlooked.

WEAKNESSES

As mentioned above, the biggest weakness in this paper is the lack of clarity about the technical aspects. I noticed several typos and omissions in the equations. Since this is a methods paper, the description of the methods should be well polished and unfortunately they are not at this time.

The methods are validated on a small truth set of 374 manually curated variants. This seems to me to be a really small number and as the authors admit, potentially biased. For reasons such as these, most new methods are evaluated on both simulated and empirical datasets. While no simulated data is as messy as real data, simulations are still useful for evaluating methods. The paper would be improved if the authors tested their methods on a simulated dataset in which the truth is known. This could be

accomplished by completely simulating data or introducing simulated mutations into their dataset. They could also down-sample high-quality validated datasets like Platinum Genomes to validate their methods.

TrioDNM model

The quantity Qx where `x` is {A, C, G, or T} estimates the probability that at **least one base call of `x` is not an error**. This is then used in various ways to calculate allelic likelihoods. This is a big change from using a multinomial or Dirichlet-multinomial distribution for genotype likelihoods, and think it deserves more discussion and justification than is currently in the paper.

Equation (6). This equation does not explain what happens when a != b and x is not in {a,b,c,d}. From looking at the source code it appears that it uses `1` for this final case. This should produce some weird likelihoods such that the probability of Dm = {A, A, A, A} given Gm = {C, G} and Gc = {G, G} is higher than Dm = {A, A, A, A} given Gm = {A, A} and Gc = {G, G}. While this may not matter when identifying the most likely mutation pattern, it should definitely affect how the calculation of equation (3). If the authors are using a trick to avoid this, they should clarify that in the paper.

VFRS

I have no statistical institution about what VRFS represents. I don't understand how the f_i's or sigma_i's were calculated or why they are squared. I also don't understand why there is a `1 +` in the calculation as well. As such, I have a hard time understanding how VRFS should work. The authors should include more motivation and explanation for the VRFS statistic.

Other Weaknesses

The DNG and TDNM scores are not defined, which makes interpreting the results difficult. They appear to be log-probabilities, but the paper also describes them as bins. The authors need to clarify how the scores are calculated and used.

The manuscript does not describe how DNG and TDNM models were parameterized. The authors should discuss whether changing the values of parameters used in DNG and TDNM models would change the results of the model.

The paper claims that VRFS and TDNM scores are not correlated and provide complementary information. I am not sure how this conclusion was made. The authors could create a scatter plot of TDNM vs VRFS to justify this claim.

OTHER COMMENTS

Equation (1). This equation contains a typo. It should be

`L(Gc, Gm, Gf | D) = P(D | Gc, Gm, Gf) x P(Gc | Gm, Gf) x P(Gm, Gf)`

where the second term on the right has the child's genotype in it.

Equation (3). The authors describe this as the posterior probability of the variant being a denovo mutation. This is not accurate. Equation (3) is the probability of the mostly likely genotype combination that is incompatible with Mendelian inheritance (given observed data). The probability that there is a denovo mutation in the child given data is

P(DNM | D) = sum(P(DNM | D, Gc, Gm, Gf) * P(D, Gc, Gm, Gf)) / sum(P(D, Gc, Gm, Gf))

where the numerator is also calculated by summing over possible genotype combinations. For example, DeNovoGear's `call` algorithm calculates this statistical accurately while it's `dnm` algorithm generates one like in this paper.

And finally, I think that the probability of a variant being a DNM (as used in the text) would be calculated

by fixing the genotype of the child and summing over the parents.

P(DNM | D, Gc = g) = sum(P(DNM | D, Gc = g, Gm, Gf) * P(D, Gc = g, Gm, Gf)) / sum(P(D, Gc = g, Gm, Gf))

To be clear, there is nothing wrong about Equation (3); however, the text doesn't accurately describe it.

Equation (5). The quantity Qx is described as the "sum of base/mapping qualities in log space". This is not correct. The relationship between Qx and the sum of base/mapping qualities (QS[x]) is Qx = 1 - 10^(-QS[x]/10). This should be corrected, or replaced by a statement that Qx can be calculated from QS.

Equation (6). I believe that only the reads from a mother or father are used to calculate Qx. The authors should clarify this by using notation for individual specific datasets in the paper. For example, if D = {Dm, Df, Dc}, then equation (6) could be written as A(Dm | Gm = ab, Gc = cd) for the mother's allelic likelihood.

Methods

Are the methods appropriate to the aims of the study, are they well described, and are necessary controls included? Choose an item.

Conclusions

Are the conclusions adequately supported by the data shown? Choose an item.

Reporting Standards

Does the manuscript adhere to the journal's guidelines on minimum standards of reporting? Choose an item.

Choose an item.

Statistics

Are you able to assess all statistics in the manuscript, including the appropriateness of statistical tests used? Choose an item.

Quality of Written English

Please indicate the quality of language in the manuscript: Choose an item.

Declaration of Competing Interests

Please complete a declaration of competing interests, considering the following questions:

- Have you in the past five years received reimbursements, fees, funding, or salary from an organisation that may in any way gain or lose financially from the publication of this manuscript, either now or in the future?
- Do you hold any stocks or shares in an organisation that may in any way gain or lose financially from the publication of this manuscript, either now or in the future?

- Do you hold or are you currently applying for any patents relating to the content of the manuscript?
- Have you received reimbursements, fees, funding, or salary from an organization that holds or has applied for patents relating to the content of the manuscript?
- Do you have any other financial competing interests?
- Do you have any non-financial competing interests in relation to this paper?

If you can answer no to all of the above, write 'I declare that I have no competing interests' below. If your reply is yes to any, please give details below.

I declare that I have no competing interests

I agree to the open peer review policy of the journal. I understand that my name will be included on my report to the authors and, if the manuscript is accepted for publication, my named report including any attachments I upload will be posted on the website along with the authors' responses. I agree for my report to be made available under an Open Access Creative Commons CC-BY license (http://creativecommons.org/licenses/by/4.0/). I understand that any comments which I do not wish to be included in my named report can be included as confidential comments to the editors, which will not be published.

Choose an item.

To further support our reviewers, we have joined with Publons, where you can gain additional credit to further highlight your hard work (see: https://publons.com/journal/530/gigascience). On publication of this paper, your review will be automatically added to Publons, you can then choose whether or not to claim your Publons credit. I understand this statement.

Yes Choose an item.
