# Supplementary material for: Improved discovery of de novo mutations using TrioDNM and VRFS
Source: Gigascience. 2026 Jun 9;15:giag068. doi: 10.1093/gigascience/giag068 (PMC13289757; doi:10.1093/gigascience/giag068)
Supplement: giag068_Reviewer_2_Report_revision_1 — Reviewer 2 -- 5/8/2026 [file giag068_reviewer_2_report_revision_1.pdf]

Reviewer Report

Title: Improved discovery of de novo mutations using TrioDNM and VRFS

Version: Revision 1 **Date:** 5/8/2026

Reviewer name: Reviewer 2

Reviewer Comments to Author:

I would like to thank the authors for revising their manuscript according to the our comments. This version is significantly improved. It is more clear and includes more details about the methods. They've also added some new methods which has given me new things to think about. I also appreciate the results now include more manually curated sites and simulated data as well.
Qx statistic and allelic likelihoods.
I think that I better understand the Qx statistic in this version. Roughly, it is a statistic whose value is governed by estimates of sequencing and mapping error when coverage is low but approaches 1 when coverage is high. In turn, allelic likelihoods are near 1 if there is a lot of coverage and no reads disagree with the genotype, and near 0 if some reads disagree with the genotype. If my understanding is correct, I recommend adding a few more sentences in the ALM description to make this intuition more clear to the reader.
Unlike genotype likelihoods which measure P(the set of base calls | genotype), allelic likelihoods seem to measure P(the alleles in the genotype are supported and no other alleles are supported | genotype). If the DNG model approximates the P(de novo mutation | data), would the ALM model approximate P(de novo mutation & parent data contains no systematic artifacts | data)?
The Dirichlet-multinomial model.
I appreciate the authors using a Dirichlet-multinomial model for representing genotype likelihoods as I have found it superior to standard multinomial models for calculating genotype likelihoods from allele depths. However, the authors appear to only use it for parental genotype-likelihoods, but I see no reason why this it should not also be used for child genotype-likelihoods. I would appreciate additional justification for this choice.
The authors do not use the Dirichlet-multinomial model directly to identify candidate sites for de novo mutations. Instead they augment their estimate of the probability that a site is a de novo mutation with three additional probability calculations: P_noise, P_parent, and P_mosaic. I am not completely clear how these three probabilities are calculated. How are the `k`s for P_noise and P_parent calculated? And is the `m` in P_mosaic the VAF (fraction of non-reference base calls?) for the site? More clarity would improve this part

of the paper.
In addition, I think the paper would benefit from an analysis justifying the use of these augmenting factors. My intuition is that some of these values may be double counting things already taken care of in the DMM model, and a comparison of unaugmented and augmented scores would help motivate the additional complexity.
VRFS.
My understanding of VRFS is that there is a reference histogram constructed from "good" sites that describes how often an individual's VAF falls into a frequency bin. If a de novo is called at site X, then the VRFS measures how well the VAF pattern of site X fits into the reference histogram.
VRFS is approximately a transformed log-likelihood of the observed bin proportions under the assumption that the proportions of empirical VAFs are independent normal variables with mean 0, and sd sigma_i. It took me a while to fully understand this formation. I think that this is because of the use of `f` to represent the fraction of VAFs in a bin, and I kept interpreting it as representing a VAF frequency. Maybe using `p` or `q` for proportion would be better here? The calculation is also a bit confusing because `bar(f)_i` is calculated for the reference and used when estimating sigma^2, but is also assumed to be zero when calculating VRFS. I recommend the authors tweak this description to improve clarity.
However, wouldn't using the reference bin proportions directly in a goodness-of-fit test be more straight forward than the current VRFS statistic? I think that a Pearson's chi-square test, a G-test, or similar test would be appropriate here to test the hypotheses that the observed frequency bins have the same distribution as a reference set. Is there any reason to suspect that VRFS would perform better or be easier to calculate than a more well known GOF tests?

Methods

Are the methods appropriate to the aims of the study, are they well described, and are necessary controls included? Choose an item.

Conclusions

Are the conclusions adequately supported by the data shown? Choose an item.

Reporting Standards

Does the manuscript adhere to the journal's guidelines on [minimum standards of reporting?]() Choose an item.

Choose an item.

Statistics

Are you able to assess all statistics in the manuscript, including the appropriateness of statistical tests used? Choose an item.

Quality of Written English

Please indicate the quality of language in the manuscript: Choose an item.

Declaration of Competing Interests

Please complete a declaration of competing interests, considering the following questions:

- Have you in the past five years received reimbursements, fees, funding, or salary from an organisation that may in any way gain or lose financially from the publication of this manuscript, either now or in the future?
- Do you hold any stocks or shares in an organisation that may in any way gain or lose financially from the publication of this manuscript, either now or in the future?
- Do you hold or are you currently applying for any patents relating to the content of the manuscript?
- Have you received reimbursements, fees, funding, or salary from an organization that holds or has applied for patents relating to the content of the manuscript?
- Do you have any other financial competing interests?
- Do you have any non-financial competing interests in relation to this paper?

If you can answer no to all of the above, write 'I declare that I have no competing interests' below. If your reply is yes to any, please give details below.

I declare that I have no competing interests

I agree to the open peer review policy of the journal. I understand that my name will be included on my report to the authors and, if the manuscript is accepted for publication, my named report including any attachments I upload will be posted on the website along with the authors' responses. I agree for my report to be made available under an Open Access Creative Commons CC-BY license (http://creativecommons.org/licenses/by/4.0/). I understand that any comments which I do not wish to be included in my named report can be included as confidential comments to the editors, which will not be published.

I agree to the open peer review policy of the journal

To further support our reviewers, we have joined with Publons, where you can gain additional credit to further highlight your hard work (see: https://publons.com/journal/530/gigascience). On publication of this paper, your review will be automatically added to Publons, you can then choose whether or not to claim your Publons credit. I understand this statement.

Yes Choose an item.
